# Hypoxia-induced PLOD3 activates the NEDD4/GOT2 axis to exacerbate renal cell carcinoma progression

**DOI:** 10.1038/s41419-026-08961-1

**Published:** 2026-06-10

**Authors:** Yan Xu, Yu Lun, Shiyue Wang, Yu Jiao, Shikai Shen, Yumeng Yan, Xiaoyu Sun

**Affiliations:** 1 Department of Urology, Guangdong Provincial People’s Hospital (Guangdong Academy of Medical Sciences), Southern Medical University, Guangzhou, Guangdong Province 510080 China; 2 The First Hospital of China Medical University, Shenyang, Liaoning Province 110001 China; 3 Department of Vascular and Thyroid Surgery, The First Hospital of China Medical University, Nanjing Bei Street No. 155, Heping District, Shenyang, Liaoning Province 110001 China; 4 Department of Hematology, The First Hospital of China Medical University, Nanjing Bei Street No. 155, Heping District, Shenyang, Liaoning Province 110001 China; 5 Department of Clinical Nutrition, Shengjing Hospital of China Medical University, Sanhao Street No. 36, Heping District, Shenyang, Liaoning Province 110004 China; 6 NHC Key Laboratory of Advanced Reproductive Medicine and Fertility, China Medical University, National Health Commission, Shenyang, China; 7 Center of Reproductive Medicine, Department of Obstetrics and Gynecology, Shengjing Hospital of China Medical Univercity, Shenyang, China

**Keywords:** Renal cell carcinoma, Cancer, Ubiquitylation, Mechanisms of disease

## Abstract

Treating renal cell carcinoma (RCC) is clinically challenging because the characteristic hypoxic microenvironment of RCC serves as a key driver of malignant progression. High-throughput sequencing analysis across multiple models was used in this study to identify PLOD3 as a major oncogene driving RCC development. PLOD3 promoted RCC cell growth and metastatic capacity in vivo and in vitro. Mechanistically, HIF-2α and histone lactylation modification jointly upregulate PLOD3 expression levels. PLOD3 specifically binds to the C2 domain of NEDD4, hindering NEDD4 autoinhibition and triggering NEDD4 to activate E3 ubiquitin ligase function. This process affects GOT2 expression via inducing K48-linked ubiquitination, leading to GOT2 degradation via the ubiquitin-proteasome pathway. Thus, PLOD3 participates in reprogramming glutamine metabolism through influencing the stability GOT2 protein. This study establishes PLOD3 as a key oncogenic driver of RCC, with HIF-2α/PLOD3/NEDD4/GOT2 pathway potentially being a therapeutic target and providing biomarkers for treating RCC.

## Introduction

Renal cell carcinoma (RCC) is a common malignant tumor that seriously threatens human health. RCC incidence is increasing, with epidemiological data indicating that this trend is occurring in younger patients [1]. Clear cell RCC (ccRCC) is a histological subtypes of RCC that accounts for over 70% of all RCC cases and more than 80% of metastatic cases [2]. Surgical resection remains the primary treatment for early stage RCC; however, approximately 30% of patients present with distant metastases at diagnosis [3]. RCC is broadly resistant to conventional chemotherapy and radiotherapy; as such, treatment options for metastatic RCC remain limited, with a five-year overall survival rate of approximately 12% [4, 5]. Therefore, identifying novel therapeutic targets for RCC would have theoretical and experimental implications for advancing diagnostic and treatment strategies.

The RCC tumor microenvironment is characterized by hypoxia, where the sustained HIF-2α expression levels regulate the transcriptional activity of multiple proto-oncogenes, promoting tumor progression [6, 7]. Additionally, hypoxia induces tumor metabolic reprogramming; lactate is a key end-product of glycolysis that plays a central role in this process [8]. Histone lactylation is an epigenetic modification that directly participates in transcriptionally regulating chromatin-associated genes, driving the acquisition of malignant phenotypes [9].

PLOD3 is a procollagen-lysine 2-oxoglutarate 5-dioxygenase (PLOD) family member that is overexpressed in various cancers [10, 11]. For example, hypomethylation of the PLOD3 promoter region leads to PLOD3 overexpression in colorectal cancer, disrupting TM9SF ubiquitination and degradation. This process promotes tumor progression through activating autophagy [10]. PLOD3 enhances glioblastoma migration and invasion [11]. Neural precursor cell expressed developmentally downregulated gene 4 (NEDD4) is a highly conserved E3 ubiquitin ligase with an HECT domain. NEDD4 is critical in disease progression [12, 13]. Glutamic-oxaloacetic transaminase 2 (GOT2) catalyzes the conversion of glutamate and oxaloacetate to α-ketoglutarate and aspartate in mitochondria, regulating cellular glutamine metabolic reprogramming [14]. However, the biological functions and specific mechanisms of action of PLOD3, NEDD4, and GOT2 in RCC remain unclear. Further study of their functions in RCC may lead to the identification of the pathophysiological mechanisms underlying this disease given their potential roles in various cancer processes, providing theoretical support for the development of targeted therapies.

We found that hypoxia-induced HIF-2α and the acidic tumor microenvironment substantially upregulate PLOD3 expression in RCC, where PLOD3 plays a pivotal role in glutamine metabolism and disease progression. Further mechanistic studies revealed that PLOD3 forms a protein complex with NEDD4 and GOT2, promoting the NEDD4-mediated degradation of GOT2 via the proteasome pathway. This process disrupts glutamine metabolism and drives RCC progression. Collectively, we confirmed that PLOD3 performs oncogenic functions in RCC and is a promising therapeutic target.

## Materials and methods

### Cell culture

Human normal renal tubular epithelial cells (HK-2) and RCC cell lines (786-O, ACHN, OSRC-2, Caki-1, and 769-P) were obtained from the Chinese Academy of Sciences Cell Bank (Shanghai, China) in 2023. 786-O (CVCL_1051) and ACHN (CVCL_1074) cells were cultured in RPMI 1640 (HyClone) and MEM (HyClone) cell culture media. The 786-O and ACHN cell lines have been authenticated by STR profiling. All lines were confirmed to be free of mycoplasma contamination. For lentiviral transfections, we followed the manufacturer’s instructions for transfection, followed by puromycin (Gibco) selection. The lentiviral target sequences used are listed in Table S3.

### CCK-8 assay

Take logarithmic growth phase 786-O and ACHN cells. Add 100 μL of cell suspension per well to a 96-well plate. Allow cells to settle uniformly for 30 min, then transfer to a CO₂ incubator for overnight culture. Add 10 μL of CCK-8 reagent per well. Incubate at 37 °C for 2 h. Detect and record results using a microplate reader.

### Plate clonogenic assay

The cells under investigation are to be taken from the logarithmic growth phase of 786-O and ACHN. The cell suspensions are then distributed uniformly across six-well plates. The 6-well plates should be placed in a 5% CO2 incubator set to 37 °C, with the medium being changed every 3 days. Cell growth should be observed. Following a 2-week period, the medium should be removed and the cells gently washed with PBS. The addition of 4% paraformaldehyde to each well is required for the purpose of fixation. The fixative must then be discarded and crystal violet staining solution must be added. The samples should then be subjected to incubation at room temperature, after which they should be photographed under a microscope.

### Transwell assay

The cells under investigation are 786-O and ACHN, which are in the logarithmic growth phase. The serum-free cell suspension is then added to the upper chamber of the Transwell, with the chemokine-containing medium being added to the lower chamber. The chamber should then be placed in a 24-well plate and incubated in a cell culture incubator. Following a 48-h period, the chamber should be removed. The upper chamber should then be washed with Phosphate Buffered Saline (PBS) in order to remove any non-migrated cells. The cells in the lower chamber were then fixed with 4% paraformaldehyde, washed with phosphate-buffered saline (PBS), and stained with 0.1% crystal violet. The non-migrated cells must then be wiped from the upper chamber membrane using a cotton swab. The observation and photographic documentation of migrated cells in the lower chamber is to be conducted under the microscope. The matrix gel must be diluted in serum-free medium prior to being added to the upper chamber of the Transwell apparatus. The gel should be distributed in a uniform manner and then subjected to an incubation process at a temperature of 37 °C, continuing until the matrix gel has undergone complete solidification. Subsequently, the cell invasion assay should be conducted in accordance with the aforementioned protocol.

### Wound-healing assay

First, mark horizontal lines on the bottom of a 6-well plate using a marker pen. Seed logarithmically growing 786-O and ACHN cells uniformly into the wells and incubate in a cell culture incubator. When cell confluence reaches approximately 90%, use a pipette tip perpendicular to the plate to create scratches along the marked lines. Wash away detached cells with PBS, add serum-free medium, and incubate in the cell culture incubator. Remove and photograph at specific time points (0 h and 48 h). Image J was used to calculate the area and the control group was compared to calculate the relative ratio change value.

### Clinical histology and immunohistochemistry analysis

Tumor tissue samples were obtained from the Department of Pathology, The First Affiliated Hospital of China Medical University. All tumor tissue samples were collected with informed consent from patients and in accordance with ethical guidelines (No.: 2022-48-2). Sample size was estimated using G*Power software (version 3.1) (F test, ANOVA: Fixed effects, omnibus, one-way). Sections were sequentially immersed in xylene to remove paraffin, followed by hydration with graded ethanol (100%, 95%, 75%), and high-pressure repair. Sections were then incubated with 3% hydrogen peroxide at room temperature to block endogenous peroxidase activity and reduce background staining. Sections were blocked with PBS containing goat serum. Primary antibodies diluted in PBS were applied dropwise to sections and incubated overnight at 4 °C. Sections were rinsed with PBS, followed by application of enzyme-labeled (HRP) secondary antibodies. Chromogenic substrate was added, and color development was observed under a microscope.

### Real-time PCR

Prepare target tissues and cells. Extract total RNA sequentially using Trizol reagent (Invitrogen), chloroform, isopropanol, and ethanol. Perform reverse transcription using PrimeScript RT Mix (Takara). Finally, conduct real-time PCR using SYBR Taq II Mix (Takara). Primer sequences are listed in Table S2.

### Western blot analysis

Protein samples were loaded into electrophoresis chambers. After electrophoresis, membranes were transferred and blocked overnight in TBST containing skim milk powder and primary antibody. Membranes were washed three times with TBST, incubated with secondary antibody at room temperature for 1 h, and subjected to chemiluminescence detection. Antibodies used are listed in Table S1.

### Cycloheximide (CHX) chase assay

The protein half-life was determined using a Cycloheximide (CHX) Chase Assay. Cell culture medium was replaced with complete medium containing the protein synthesis inhibitor CHX (100 µg mL⁻¹). Cells were harvested at 0,3,6, and 9 h post-treatment for protein extraction.

### ChIP assay

Using a ChIP extraction and detection kit (Millipore), follow the manufacturer’s instructions: first immerse target cells in formaldehyde, then sequentially incubate in glycine and protease inhibitors before sonicating to form genomic DNA fragments. The target antibody was then added to the mixture using protein-agarose beads, incubated at 4 °C, washed in buffered solutions, and purified using a DNA Spin column after heating. Finally, detection was performed via real-time PCR.

### Luciferase reporter assay

PLOD3-wt and PLOD3 truncated regulatory elements were cloned upstream of the firefly luciferase gene in a reporter vector. A control plasmid carrying the sea squirt luciferase gene served as a reference. After transfecting 293 T cells with these vectors, cells were lysed and luciferase activity was measured.

### Immunofluorescence staining

Discard the culture medium. After washing with PBS, fix with paraformaldehyde. Following fixation, wash with PBS again. Then, perform membrane lysis using Triton X-100 on a shaking platform. After membrane disruption, wash with PBS and block with BSA on a shaking platform. Incubate the cells with the corresponding primary antibody overnight at 4 °C, then add the secondary antibody and incubate at room temperature. Incubate with DAPI at room temperature for nuclear staining. Wash with PBS and observe using a confocal microscope.

### mtROS flow cytometry detection

Digest and centrifuge collected cells, then count them. Resuspend 1 × 10⁶ cells in MitoSOX™ dye diluted 1:1000 in serum-free medium. Mix thoroughly and incubate at 37 °C in the dark for 30 min. After incubation, wash twice with PBS, resuspend in PBS, filter through a mesh filter, and transfer to flow cytometry tubes for analysis.

### GSH/GSSG and NADP + /NADPH ratio assays

Collected cells were lysed and centrifuged. The GSH/GSSG ratio was determined using the GSH/GSSG Ratio Assay Kit (Beyotime; S0053), and the NADP + /NADPH ratio was measured using the NADP + /NADPH Ratio Assay Kit (Beyotime; S0179) according to manufacturer instructions. Convert sample absorbance values to GSH/GSSG and NADPH/NADP+ concentrations using the standard curve, then calculate the ratios.

### Animal studies

For subcutaneous tumor formation experiments, 4-week-old female BALB/c-nu nude mice were selected. A mixture of cells in logarithmic growth phase and matrix gel was injected subcutaneously into the dorsal region of the nude mice. Sample size was estimated using G*Power software (version 3.1) (F test, ANOVA: Fixed effects, omnibus, one-way). After disinfection with alcohol swabs, the needle was inserted obliquely into the subcutaneous tissue to inject the cell suspension. Tumor volume was calculated using the formula: V = 0.5 × a × b² (mm³). At the experimental endpoint, nude mice were euthanized, and tumor tissues were harvested. All BALB/c nude mice were housed at the Animal Experiment Center of China Medical University.

For the in vivo lung metastasis model, select 6-week-old female BALB/c-nu nude mice and tumor cells in the logarithmic growth phase, then prepare a single-cell suspension. Secure the nude mouse in a restraint device, wipe the tail with alcohol, and slowly inject 100 μl of the cell suspension at a suitable location. At the experimental endpoint, nude mice were euthanized, and intact lungs were removed. These were sequentially fixed in formalin, paraffin-embedded, sectioned, and subjected to HE staining for microscopic observation of tumor cell aggregation. We employed a completely randomized design as the randomization strategy for experimental mice. A double-blind procedure was implemented to ensure blinding. Euthanasia was performed when any of the following criteria were met: tumor volume reached a predetermined size, tumor weight exceeded 10% of body weight, or the mouse exhibited significant mobility impairment, 20% weight loss, inability to consume food and water normally, or lethargy. All BALB/c nude mice were housed at the Animal Experiment Center of China Medical University. All animal procedures were conducted according to institutional guidelines approved by the Animal Care and Use Ethics Committee of the China Medical University Animal Research Center (No.: CMU20251934).

### Bioinformatics analysis

Bioinformatics analysis was performed using the TCGA-KIRC and TCGA-KIRP datasets (https://portal.gdc.cancer.gov/), GEO datasets (GSE15641, GSE36895 and GSE53757) (https://www.ncbi.nlm.nih.gov/gds/) and the UALCAN database (http://ualcan.path.uab.edu/analysis-prot.html), we analyzed PLOD3 expression and its clinical relevance in RCC patients. Genome-wide enrichment analysis (Gene Set Enrichment Analysis, GSEA) was performed using R software to investigate the enrichment relationship between PLOD3 and hypoxia and EMT.

### Statistical analysis

Statistical analysis was performed using GraphPad Prism software (9.0.1). Differences between groups were evaluated using Student’s *t* test in GraphPad Prism. Differences in variance between groups were assessed using Levene’s test to confirm that no extreme differences in variability were present. Data are presented as the mean ± SD of at least three independent experiments. Values of *P < 0.05, **P < 0.01 or ***P < 0.001were considered statistically significant.

## Results

### Identification of PLOD3 as a key regulator of RCC progression induced by hypoxia

**Fig. 1:**
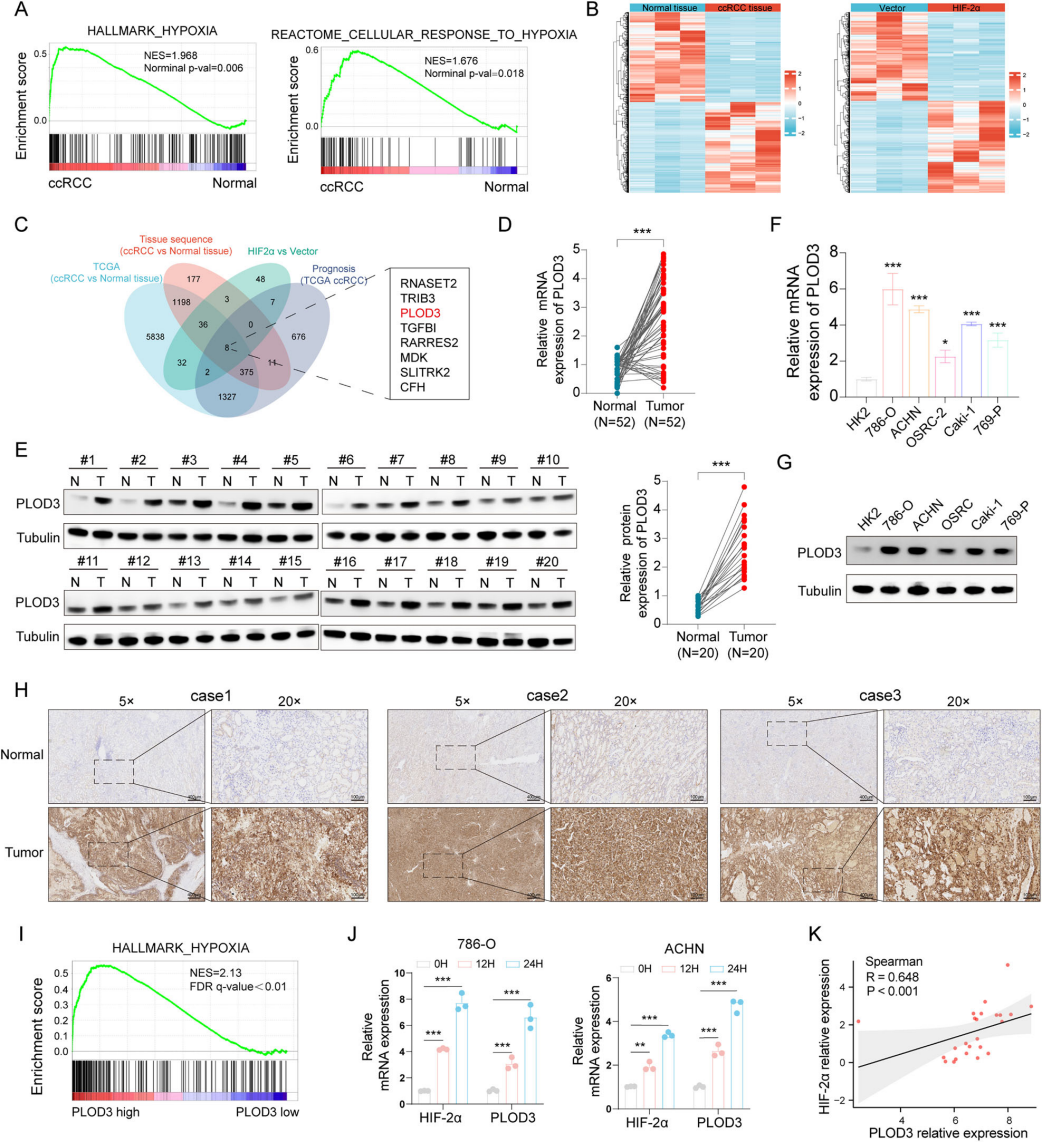
PLOD3 as hypoxia-induced factor involved in ccRCC progression. **A** GSEA enrichment analysis of ccRCC tumor tissue and adjacent normal tissue. **B** Heatmap based on three pairs of ccRCC tumor and adjacent normal tissue samples, and differences between 786-O cells overexpressing HIF-2α and untreated controls. **C** Venn diagram showing intersections of ccRCC tumors and adjacent normal tissues from the TCGA database, differences between ccRCC tumors and adjacent normal tissues, differences between 786-O cells overexpressing HIF-2α and untreated controls, and genes with prognostic significance in ccRCC from the TCGA database. **D** mRNA expression differences in RCC tumor tissues and matched adjacent normal tissues (*n* = 52). **E** Protein expression differences in RCC tumor tissues and matched adjacent normal tissues (*n* = 20). **F**, **G** mRNA and protein expression differences of PLOD3 in normal renal epithelial cells and tumor cells. **H** Immunohistochemical staining of PLOD3 in ccRCC tumor tissues and matched adjacent normal tissues. **I** GSEA enrichment analysis of PLOD3 high-expression and low-expression groups. **J** Changes in mRNA expression levels of PLOD3 and HIF-2α in 786-O and ACHN cells under hypoxic conditions. **K** Correlation between mRNA expression levels of PLOD3 and HIF-2α in ccRCC tumor tissues. *P < 0.05, **P < 0.01, ***P < 0.001.

Hypoxia-HIF2α signaling is a hallmark of RCC. Our enrichment analysis using TCGA-KIRC tumor and normal tissue sequencing data confirmed this finding, consistent with previous reports (Fig. 1A) [15, 16]. To find out potential genes might be involved in the development of RCC, we looked at how some genes were expressed differently in samples of RCC clinical tissue and in RCC cells that had overexpressing HIF2α via RNA-seq (Fig. 1B). These were then compared with lists of genes that were either more or less active in TCGA-KIRC and associated with a poor prognosis (Fig. 1C). Among the 8 candidate genes screened, we found that a certain gene (PLOD3) was much more active in RCC tissue than in normal tissue nearby (Fig. S1A). A previous study had identified PLOD3 as associated with poor prognosis in RCC through data analysis [17], yet its specific function and contribution to tumor progression mechanisms remained to be elucidated. Next, we examined PLOD3 expression in RCC tissues and matched normal tissues. Compared to normal tissues, PLOD3 was elevated at both mRNA and protein levels in tumor tissues, as demonstrated by real-time quantitative polymerase chain reaction (qRT-PCR), Western blotting, and immunohistochemistry (Fig. 1D, E, and Fig. 1H). Additionally, we examined PLOD3 expression in various RCC cell lines. Compared to the normal human renal epithelial cell line HK2, both PLOD3 mRNA and protein expression were significantly elevated in RCC cell lines (Fig. 1F, G). Big data analysis results aligned with our experimental findings. Analyses of the TCGA-KIRC and TCGA-KIRP combined GTE-X dataset, GEO datasets, and CPTAC database all demonstrated significantly elevated PLOD3 mRNA and protein expression in tumor tissues relative to normal controls (Fig. S1B, C). Similarly, elevated PLOD3 levels correlated with higher clinical grades, sunitinib resistance, and shorter OS, DSS, and PFI in RCC (Fig. S1D–F). Although previous studies reported PLOD3 hypoxia dependence [11], the relationship between PLOD3 and HIF2α required further evaluation. We therefore first performed single-gene GSEA pathway enrichment analysis for PLOD3 in the TCGA-KIRC dataset, revealing a positive correlation between PLOD3 expression and hypoxia (Fig. 1I). Subsequently, RCC cells were cultured under hypoxic conditions, revealing elevated mRNA expression of PLOD3 and HIF2α in both 786-O and ACHN cell lines (Fig. 1J). Furthermore, analysis of RCC tissue samples and the TCGA database revealed a positive correlation between HIF2α and PLOD3 expression (Fig. 1K and S1G). These findings establish PLOD3 as a key factor mediating poor prognosis in RCC through hypoxia-induced HIF2α signaling.

### Hypoxia drives PLOD3 expression in RCC

**Fig. 2:**
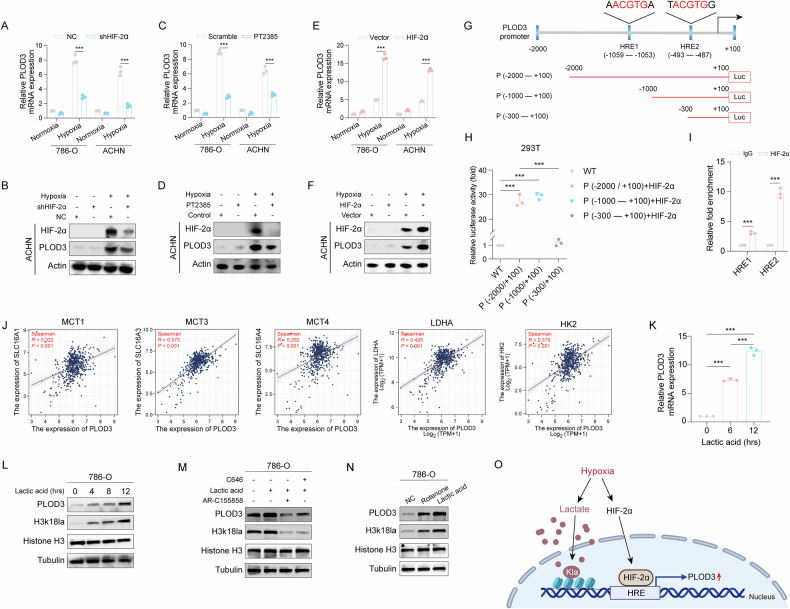
Hypoxia drives PLOD3 expression in RCC. **A**, **B** qRT-PCR and Western blot analysis validated PLOD3 and HIF-2α expression levels in ACHN cells with HIF-2α knockdown (shHIF-2α) or no treatment (NC) under normoxia or hypoxia. **C**, **D** qRT-PCR analysis and Western blot analysis validated the expression levels of PLOD3 and HIF-2α in PT2385-treated and Control ACHN cells under normoxia or hypoxia. **E**, **F** qRT-PCR analysis and Western blot analysis validated the expression levels of PLOD3 and HIF-2α in ACHN cells overexpressing HIF-2α under normoxic or hypoxic conditions. **G** Schematic of the luciferase reporter vector containing the potential hypoxia response element (HRE) for HIF-2α within the PLOD3 gene promoter and PLOD3 promoter with varying lengths. **H** Luciferase reporter assay in 293 T cells overexpressing HIF-2α under hypoxia conditions, using PLOD3 promoters of different lengths. **I** ChIP assay analysis of HIF-2α binding to the PLOD3 promoter via HRE1 and HRE2 in hypoxic 786-O cells. **J** Correlation of PLOD3 mRNA expression levels with LDHA, MCT1, MCT3, and MCT4 in the TCGA database. **K** mRNA expression levels of PLOD3 after lactate treatment. **L** Protein expression levels of PLOD3 and H3K18la after lactate treatment. **M** Western blot analysis of specific protein expression levels in RCC cells after treatment with lactate, histone acetyltransferase p300 inhibitor (C646), and lactate transporter inhibitor (AR-C155858). **N** Western blot analysis of RCC cell protein expression levels after lactate or rotenone treatment. *P < 0.05, **P < 0.01, ***P < 0.001. **O** Hypoxia induces transcriptional activation of PLOD3.

Next, we further explored the regulatory mechanism of HIF2α on PLOD3 in RCC cells. As HIF2α primarily functions as a transcription factor, we first silenced HIF2α and treated with the HIF2α inhibitor PT2385. The results demonstrated that HIF2α silencing and PT2385 treatment both significantly reduced PLOD3 mRNA and protein levels (Fig. 2A–D). Under hypoxic and untreated conditions, we found that HIF2α overexpression significantly increased PLOD3 mRNA and protein levels (Fig. 2E, F). Notably, hypoxia amplified the regulatory effect of HIF2α on PLOD3 expression. Contrasting with the silenced results, we overexpressed HIF2α. To validate HIF2α‘s transcriptional regulation of PLOD3, we first identified two hypoxia response elements (HREs) within the PLOD3 promoter region using the JASPAR database. We generated luciferase reporter plasmids by truncating PLOD3 promoter sequences of varying lengths (Fig. 2G and S2A). Luciferase assays revealed that truncating HRE2 alone caused a significant decrease in luciferase activity (Fig. 2H). Next, ChIP-qPCR analysis of HIF2α binding at predicted HRE sites revealed enrichment at both HRE1 and HRE2, particularly at HRE2, compared to the igG control, indicating PLOD3 is a direct target of HIF2α (Fig. 2I). Collectively, these data demonstrate that HIF2α transcritally upregulates PLOD3 expression under hypoxic conditions.

Given the well-established link between hypoxia and glycolysis-derived lactate, elevated lactate levels are a hallmark of RCC [18]. Further bioinformatics analysis from the TCGA database also revealed that PLOD3 positively correlates with lactate-related genes (HK2, LDHA, LDHB, and PKM2) and the MCT family (Fig. 2J and S2B). We therefore hypothesized that HIF2α-mediated transcriptional activation of PLOD3 is closely linked to lactate. To validate this hypothesis, we prioritized examining PLOD3 mRNA and protein expression levels following lactate treatment. qRT-PCR and Western blot analysis confirmed that lactate significantly upregulates both PLOD3 mRNA and protein expression (Fig. 2K, L). Histone lactylation, a process derived from lactate, has been identified as a novel form of protein modification. In consideration of the critical role of histone lactylation in the context of transcriptional regulation, a hypothesis was formulated proposing that histone lactylation modulates HIF2α‘s capacity to activate PLOD3 transcription. To validate this hypothesis, we treated RCC cells with 25 mM lactate and assessed H3 histone lactylation levels at different time points (0, 4, 8, 12 h). The results of the study indicated that lactate may regulate PLOD3 expression via H3K18la (Fig. 2L). Furthermore, treating RCC cells with the histone acetyltransferase inhibitor C646 and the MCT inhibitor AR-C155858, respectively, led to a significant reduction in H3K18la and PLOD3 expression levels compared to controls and lactate alone (Fig. 2M). It is noteworthy that treatment of 786-O cells with rotenone or lactate separately demonstrated that rotenone, as a mitochondrial inhibitor, effectively augmented endogenous lactate production. In accordance with the findings observed with lactate alone, rotenone significantly elevated H3K18la and PLOD3 expression levels in comparison with the control group (Fig. 2N). Next, we constructed lentiviral plasmids expressing wild-type (WT) and an H3 mutant with a point mutation resulting in the substitution of K18 with arginine (H3-K18R). Notably, the expression of the H3-K18R mutant significantly reduced PLOD3 expression after lactate treatment compared to that in the WT group (Fig. S2C). As demonstrated collectively in Fig. 2O, these findings suggest that H3K18la promotes the dynamic hypoxia-mediated transcription of PLOD3.

### PLOD3 promotes RCC cell growth and metastasis in vivo and in vitro

**Fig. 3:**
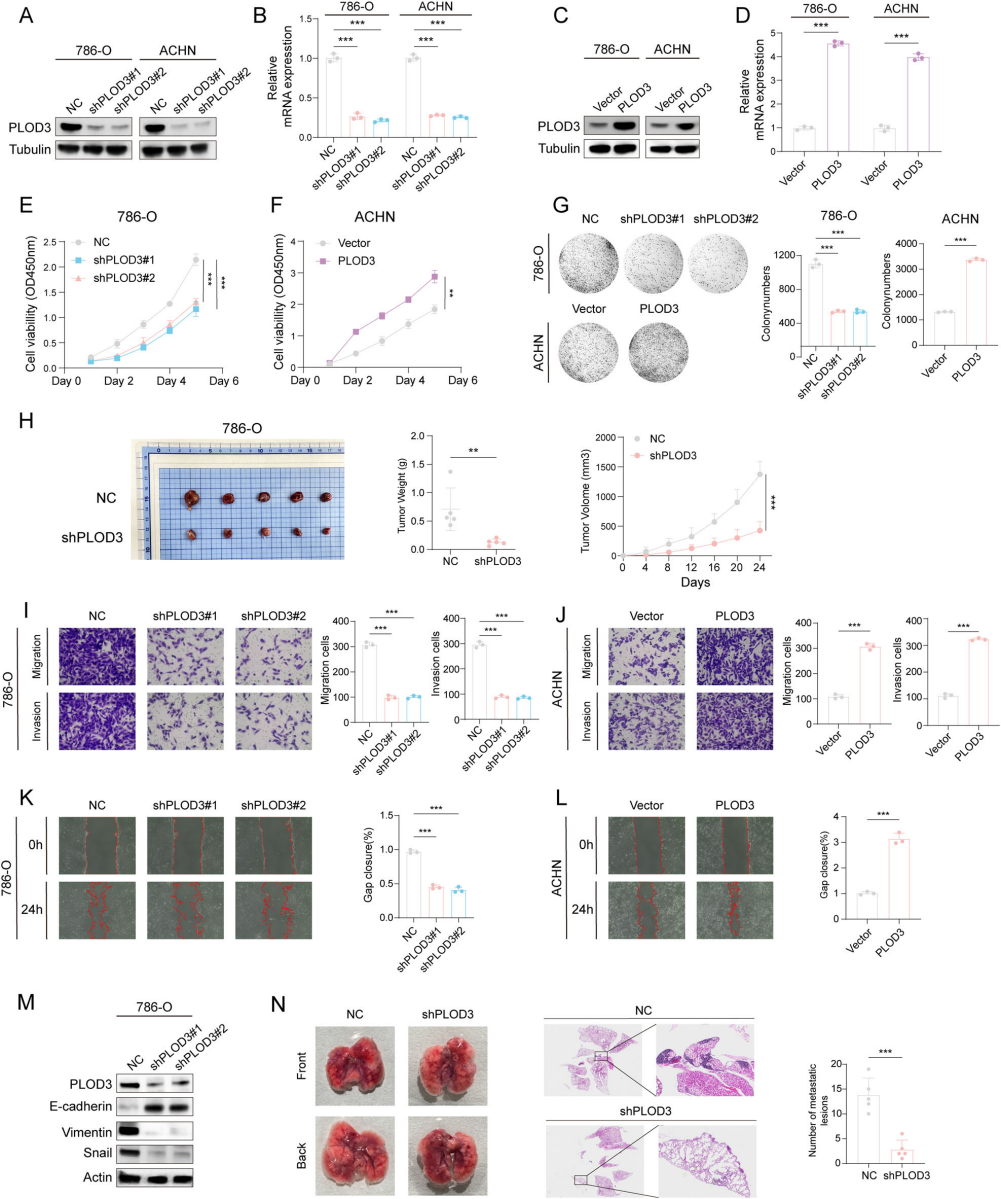
PLOD3 promotes RCC cell growth and metastasis in vivo and in vitro. **A**–**D** Immunoblotting and qRT-PCR analyses validated the efficiency of PLOD3 knockdown and overexpression in 786-O and ACHN cells. **E**, **F** CCK-8 assays in 786-O and ACHN cells following PLOD3 knockdown and overexpression. **G** Plate clonation assays and statistical charts for PLOD3 knockdown and overexpression. **H** Tumor images and statistical charts of tumor growth rate and weight in xenograft mouse models after PLOD3 knockdown in 786-O cells (*n* = 6). **I**, **J** Transwell assays and statistical charts for PLOD3 knockdown and overexpression. **K**, **L** Wound healing assays and statistical charts for PLOD3 knockdown and overexpression. **M** Western blot analysis of EMT marker protein expression levels after PLOD3 knockdown. **N** Lung metastasis images and metastasis site statistical charts of RCC cells after PLOD3 knockdown (*n* = 5). *P < 0.05, **P < 0.01, ***P < 0.001.

To evaluate the oncogenic role of PLOD3 in RCC, we established stable transfected cell lines (786-O and ACHN) with PLOD3 knockdown and overexpression, and validated PLOD3 expression at both protein and mRNA levels (Fig. 3A–D). Our findings indicate that the silencing of PLOD3 led to a significant inhibition in proliferation in RCC cell types when compared to their respective controls. Overexpression of PLOD3 significantly increased the proliferation rate of RCC cells relative to controls (Fig. 3E, F). Plate-cloning assays yielded consistent results (Fig. 3G and Fig. S3A). Subsequently, we investigated the oncogenic function of PLOD3 in vivo by implanting PLOD3-knockout 786-O cells into the lateral abdomen of BALB/c mice. These data demonstrated that PLOD3 silencing significantly inhibited 786-O cell tumor growth, including tumor weight and volume (Fig. 3H).

Consequently, we expanded our investigation to explore the function of PLOD3 in the context of EMT within RCC. Both transwell and scratch assays demonstrated that PLOD3 knockout significantly impaired migration and invasion in RCC cell lines, while PLOD3 overexpression enhanced these capabilities (Fig. 3I–L). Subsequent investigation revealed that the silencing of PLOD3 resulted in substantial inactivation of EMT. This phenomenon was further substantiated by a conspicuous augmentation in the epithelial marker E-cadherin and a substantial diminution in mesenchymal markers (including Vimentin and Snail) in both 786-O cells (Fig. 3M). The activation of the PI3K/AKT/mTOR signaling pathway in RCC is an important mechanism of carcinogenesis, closely related to EMT [19]. Meanwhile, the PLOD3 single-gene pathway enrichment analysis also enriched the PI3K/AKT/mTOR signaling pathway (Fig. S3B). Finally, we established a renal cell carcinoma lung metastasis model in nude mice via tail vein injection. Imaging and HE staining revealed a significant reduction in the number of metastatic nodules in the lungs of PLOD3-knockdown nude mice compared to controls (Fig. 3N), suggesting that PLOD3 knockdown promotes post-seeding outgrowth of renal cell carcinoma cells. Collectively, these data establish the key oncogenic role of PLOD3 in RCC.

### Formation of the PLOD3-NEDD4-GOT2 ternary complex

**Fig. 4:**
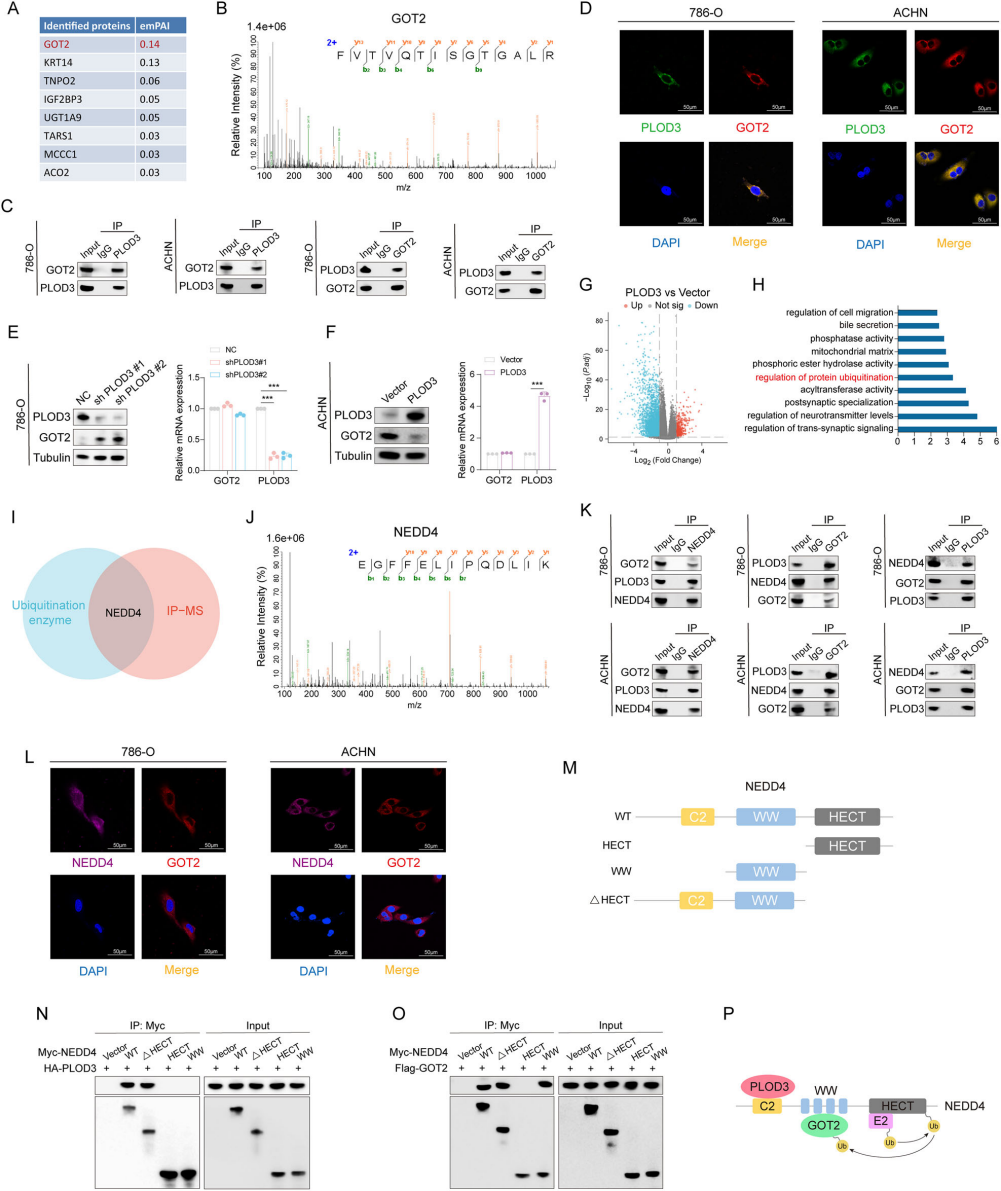
PLOD3 forms protein complexes with NEDD4 and GOT2. **A** Proteins identified by LC-MS as binding to PLOD3. **B** Fragmentation spectrum of GOT2 peptides identified by LC-MS. **C** Co-IP experiments validate the interaction between PLOD3 and GOT2 in 786-O and ACHN cells. **D** Immunofluorescence colocalization images of PLOD3 and GOT2 in 786-O and ACHN cells. **E** Protein and mRNA expression levels of PLOD3 and GOT2 after PLOD3 knockdown. **F** Protein and mRNA expression levels of PLOD3 and GOT2 after PLOD3 overexpression. **G**, **H** Volcano plot and gene set enrichment analysis of differentially expressed genes between PLOD3-overexpressing and untreated 786-O cells. **I** Venn diagram showing the intersection of predicted E3 ligases and PLOD3 from LC-MS. **J** Fragmentation spectrum of NEDD4 peptides identified by LC-MS. **K** Co-IP experiments validate interactions between PLOD3, NEDD4, and GOT2 in 786-O and ACHN cells. **L** Immunofluorescence colocalization images of NEDD4 and GOT2 in 786-O and ACHN cells. **M** Schematic diagram of full-length and truncated mutant variants of the NEDD4 protein. **N**–**P** Co-IP assay of PLOD3 detected with Myc antibody after transfection of full-length and truncated Myc-tagged NEDD4 with HA-tagged PLOD3 into 293 T cells. Co-IP assay of GOT2 detected with a Flag antibody after co-transfection of full-length and truncated Myc-tagged NEDD4 with Flag-tagged GOT2 into 293 T cells.

To investigate the potential regulatory mechanisms of PLOD3 in RCC cell lines, we performed IP-mass spectrometry analysis to identify proteins potentially interacting with PLOD3. The top 8 high-confidence proteins identified via exponentially modified protein abundance index (emPAI), with GOT2 exhibiting the highest score (Fig. 4A). Specific amino acid residue sequences and mass spectrometry data (Fig. 4B). Further validation via Co-IP and immunofluorescence colocalization experiments confirmed that PLOD3 and GOT2 co-localize and exhibit endogenous interaction in cells (Fig. 4C, D). Moonlighting enzymes refer to single polypeptide chains that perform two or more distinct functions through conformational changes or subcellular localization shifts under different microenvironmental conditions. GOT2 exhibits particularly significant moonlighting activity during malignant tumor progression [20]. To investigate this phenomenon, we first conducted data analysis, which revealed that the expression of GOT2 varied across different tumors. Specifically, in KIRC, GOT2 was expressed at a lower level compared to the control group (Fig. S4A). Subsequently, PCR and WB assays were performed. The results indicated that PLOD3 interference did not alter GOT2 mRNA levels but did affect GOT2 protein expression (Fig. 4E, F and Fig. S4B, C), suggesting that PLOD3 may influence GOT2 at the post-translational level rather than the transcriptional level. Furthermore, RNA-seq analysis of PLOD3 revealed functional enrichment in ubiquitin-mediated degradation pathways (Fig. 4G, H). In light of these findings, we hypothesize that PLOD3 may play a role in GOT2 ubiquitin-mediated degradation. In order to test this hypothesis, we first identified potential E3 ubiquitin ligases in the PLOD3-GOT2 complex. we identified NEDD4 as a candidate E3 ubiquitin ligase for PLOD3-GOT2 (Fig. 4I–J). Subsequently, we performed co-immunoprecipitation (CoIP) experiments using NEDD4, PLOD3, and GOT2 to enrich for the other two proteins. Results demonstrated endogenous interactions among PLOD3, NEDD4, and GOT2 (Fig. 4K). Additionally, immunofluorescence colocalization studies yielded similar results (Fig. 4L).

As a classical E3 ligase, NEDD4 primarily comprises an N-terminal C2 domain, an intermediate WW domain for substrate binding, and a C-terminal HECT domain for ubiquitin conjugation (Fig. 4M). In its resting state, the N-terminal C2 domain and C-terminal HECT domain of NEDD4 typically interact, inducing an autoinhibitory conformation. Only upon release from this autoinhibitory state, through binding to the C2 domain of NEDD4, is NEDD4 E3 ligase activity triggered, thereby activating NEDD4 [21, 22]. We sought to analyze the specific binding regions within the PLOD3-NEDD4-GOT2 ternary complex. Notably, by constructing partially truncated NEDD4 plasmids, we found that PLOD3 binds to NEDD4 only when its C2 domain is present, whereas GOT2 specifically binds to the WW domain of NEDD4 (Fig. 4N, O). Sara et al. reported that the WW domain is the key structural domain regulating NEDD4 subfamily recognition of substrates [23]. Collectively, these findings indicate that PLOD3 triggers NEDD4 activation through specific binding to its C2 domain. Subsequently, GOT2 is recruited as a substrate and specifically binds to NEDD4’s WW domain, forming a ternary complex among the three components.

### PLOD3 promotes the interaction between NEDD4 and GOT2 to facilitate GOT2 protein degradation

**Fig. 5:**
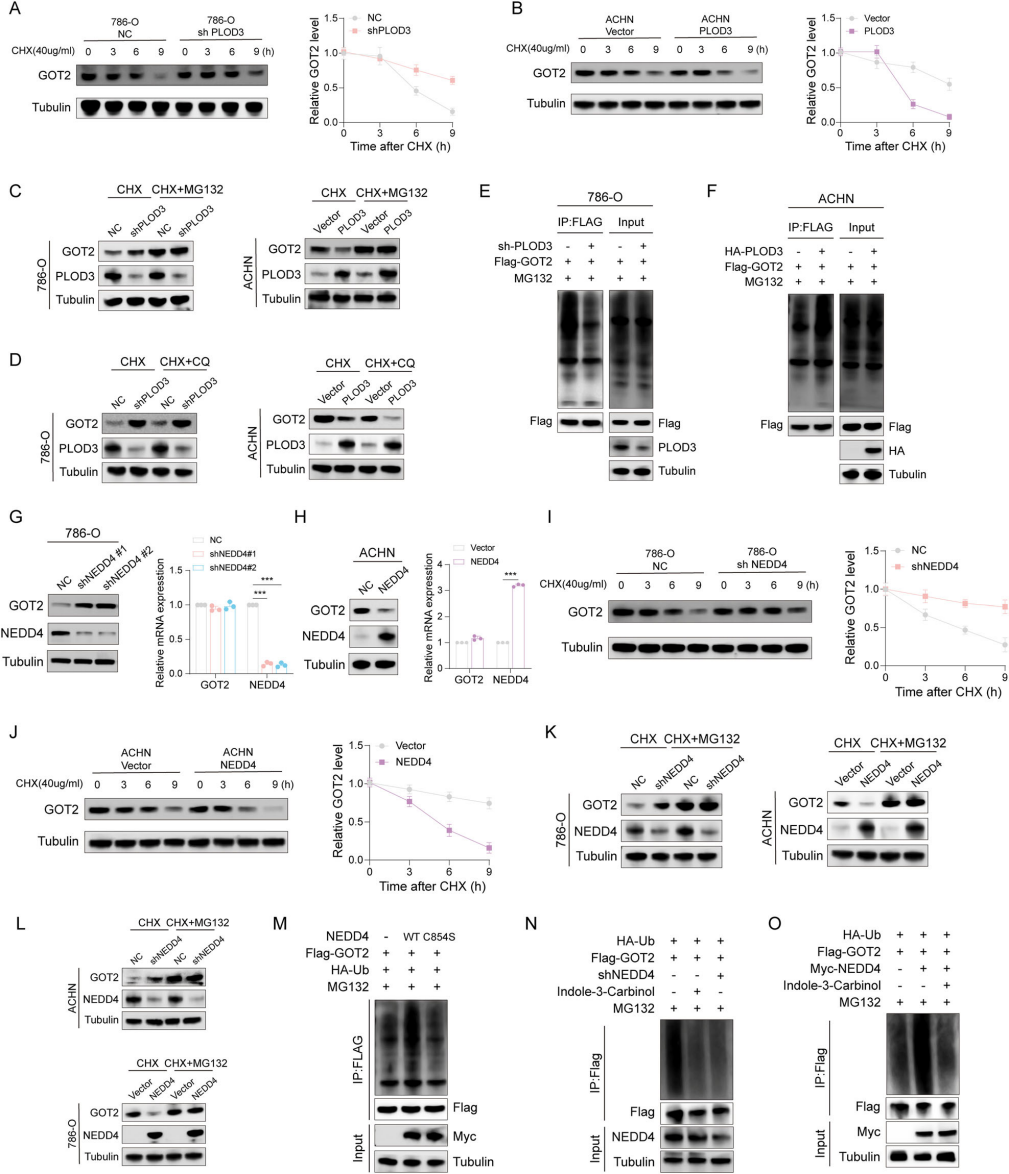
PLOD3 and NEDD4 influence GOT2 stability. **A**, **B** GOT2 protein expression following CHX treatment at different time points after PLOD3 knockdown and overexpression. **C** GOT2 protein expression after CHX or MG132 treatment following PLOD3 knockdown and overexpression. **D** GOT2 protein expression following CHX or CQ treatment after PLOD3 knockdown and overexpression. **E**, **F** GOT2 ubiquitination levels after PLOD3 knockdown and overexpression. **G** Protein and mRNA expression levels of target proteins in 786-O cells after NEDD4 knockdown. **H** Protein and mRNA expression levels of target proteins in ACHN cells after NEDD4 overexpression. **I**, **J** GOT2 protein expression at different time points after CHX treatment following NEDD4 knockdown and overexpression. **K** GOT2 protein expression after CHX or MG132 treatment following NEDD4 knockdown and overexpression. **L** GOT2 protein expression after CHX or MG132 treatment following NEDD4 knockdown and overexpression. **M** Ubiquitination levels of GOT2 following NEDD4 overexpression and mutation at a critical amino acid site (C854S). **N** GOT2 ubiquitination levels after NEDD4 knockdown and treatment with Indole-3-Carbinol. **O** GOT2 ubiquitination levels after NEDD4 overexpression and treatment with Indole-3-Carbinol. *P < 0.05, **P < 0.01, ***P < 0.001.

To further validate the hypothesis that PLOD3 influences GOT2 protein ubiquitination and degradation via the E3 ubiquitin ligase NEDD4, we treated cells with the protein synthesis inhibitor chloramphenicol (CHX) in both PLOD3-knockdown and PLOD3-overexpressing cell lines to assess GOT2 protein stability. Results showed that GOT2 protein stability increased in PLOD3-knockdown cells compared to controls; while GOT2 half-life was significantly shortened in PLOD3-overexpressing cells, indicating PLOD3 modulates GOT2 stability (Fig. 5A, B). Generally, protein degradation pathways are categorized into two types: the ubiquitin-proteasome pathway and the autophagy-lysosomal pathway. Although previous results suggested PLOD3 may participate in the ubiquitin-proteasome pathway (Fig. 4H), to better define the specific degradation pathway mediated by PLOD3 for GOT2, we treated RCC cells with either PLOD3 knockdown or overexpression with the proteasome inhibitor MG132 and the autophagy-lysosomal inhibitor CQ. Western blot results showed: MG132 reversed the reduction in GOT2 protein expression caused by PLOD3 overexpression, whereas CQ failed to produce a similar effect. This indicates that PLOD3 influences GOT2 stability and expression by affecting its ubiquitin-proteasome pathway (Fig. 5C, D and Fig. S5A-B). Furthermore, to determine whether PLOD3 ubiquitinates GOT2, we performed co-immunoprecipitation (COIP). As anticipated, knocking down PLOD3 significantly reduced the accumulation of ubiquitinated GOT2. Conversely, PLOD3 overexpression increased the accumulation of ubiquitinated PLOD3 (Fig. 5E, F). The above results indicate that HIF2α mediates the effect of PLOD3 on the ubiquitin-proteasome pathway of GOT2.

Next, we further elucidated the role of NEDD4 in the PLOD3-GOT2 interaction. We first established RCC cell lines with NEDD4 knockdown and overexpression, respectively. WB results indicated that NEDD4 interference significantly altered endogenous GOT2 protein levels without affecting mRNA expression (Fig. 5G, H and S5C-D). Similarly, CHX treatment of RCC cells with NEDD4 knockdown or overexpression revealed that NEDD4 knockdown increased GOT2 protein stability compared to controls, whereas NEDD4 overexpression produced the opposite effect (Fig. 5I–J). Subsequently, MG132 treatment of these cells yielded WB results consistent with previous PLOD3 knockdown/overexpression studies: NEDD4 knockdown elevated GOT2 protein expression, while MG132 reversed the GOT2 protein reduction induced by NEDD4 overexpression (Fig. 5K, L). Furthermore, IP analysis confirmed that wild-type NEDD4, but not the catalytically inactive C854S mutant (NEDD4-CS), promoted GOT2 ubiquitination (Fig. 5M).

Similarly, ubiquitinylation assays demonstrated that indole-3-carbinol treatment and NEDD4 knockout significantly reduced GOT2 ubiquitinylation (Fig. 5N, O). These findings indicate that NEDD4 serves as the E3 ligase responsible for destabilizing GOT2 in RCC cells.

**Fig. 6:**
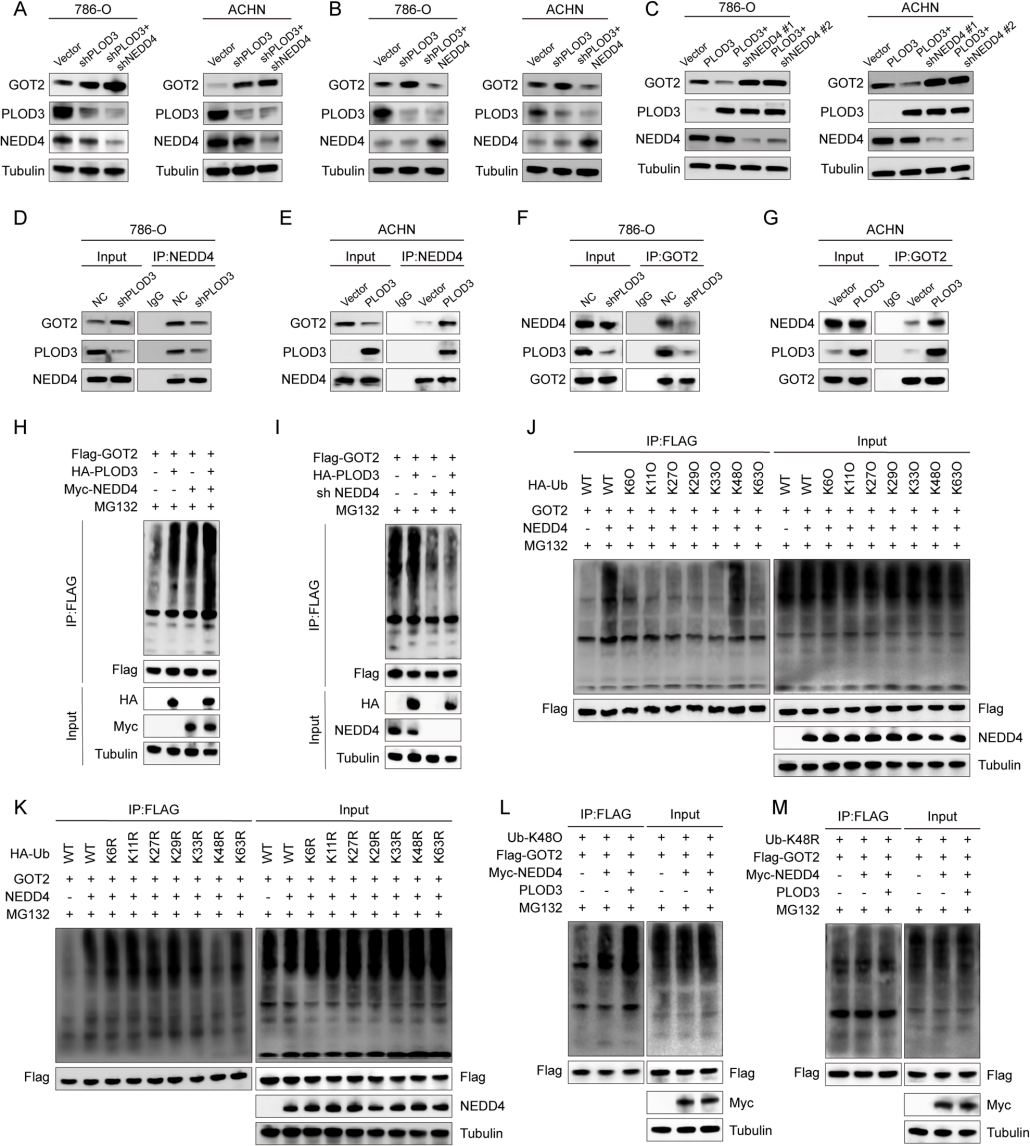
PLOD3 promotes NEDD4-GOT2 interaction to degrade GOT2 protein. **A** GOT2 protein expression levels after co-knockdown of PLOD3 and NEDD4. **B** GOT2 protein expression levels after co-treatment with PLOD3 knockdown and NEDD4 overexpression. **C** GOT2 protein expression levels after co-treatment with PLOD3 overexpression, NEDD4 knockdown. **D** Changes in NEDD4-GOT2 binding after PLOD3 knockdown, validated using NEDD4 as the IP group compared to the Input control. **E** Changes in NEDD4-GOT2 interaction following PLOD3 overexpression, validated by NEDD4 as the IP group control compared to Input. **F** Changes in NEDD4-GOT2 interaction following PLOD3 knockdown, validated by GOT2 as the IP group control compared to Input. **G** Changes in NEDD4-GOT2 interaction following PLOD3 overexpression, validated by GOT2 IP. **H** Ubiquitination levels of GOT2 upon co-overexpression of PLOD3 and NEDD4. **I** Ubiquitination levels of GOT2 following co-treatment with PLOD3 overexpression and NEDD4 knockdown. **J**, **K** Screening of Flag-GOT2 for potential lysine ubiquitination sites after transfection with wild-type (WT) or different mutant HA-Ub plasmids following NEDD4 overexpression. **L**, **M** GOT2 ubiquitination levels after NEDD4 overexpression in the presence of Ub with K48-only mutations (K48O) and Ub with K48-specific mutations (K48R).

To better understand how PLOD3-NEDD4 causes GOT2 to break down and find the most important site for ubiquitination, we constructed two cell models. In PLOD3-knockdown cells, we simultaneously knocked down or overexpressed NEDD4 to detect changes in GOT2 protein levels. Western blot (WB) analysis revealed that RCC cells transfected with PLOD3 shRNA exhibited a slight increase in GOT2 levels due to PLOD3 depletion, with this effect becoming more pronounced upon further Nedd4 knockdown (Fig. 6A). On the other hand, overexpressing Nedd4 undid the increase in GOT2 expression caused by the knockdown of PLOD3 (Fig. 6B). We also subjected the RCC cells that overexpressed PLOD3 to two different treatments. WB results showed that either NEDD4 knockdown or indole-3-carbinol treatment could restore the lower GOT2 protein levels caused by PLOD3 overexpression (Fig. 6C).

To further validate the PLOD3-NEDD4 relationship, we performed COIP on cells with PLOD3 knockdown and overexpression. Notably, results showed that PLOD3 knockdown did not affect NEDD4 expression but reduced the binding of the PLOD3-NEDD4-GOT2 ternary complex, while PLOD3 overexpression enhanced this ternary complex assembly (Fig. 6D–G and Fig. S6A–C). Consequently, ubiquitination assays consistently demonstrated that the over-expression of either PLOD3 or NEDD4 increased GOT2 ubiquitination levels, with the effect being more pronounced when both PLOD3 and NEDD4 were co-overexpressed. Knockdown of NEDD4 in the overexpression context restored GOT2 ubiquitination levels (Fig. 6H, I). These data corroborate our initial hypothesis that PLOD3 initially triggers NEDD4 activation, which subsequently recruits GOT2 for ubiquitination-mediated degradation. Furthermore, screening assays for potential lysine ubiquitination sites on Ub molecules revealed that NEDD4 significantly suppressed K48-linked polyubiquitination on GOT2 (Fig. 6J). Functional validation using lysine-to-arginine (K/R) mutants yielded identical results (Fig. 6K, M). Collectively, these findings demonstrate that PLOD3 binds to NEDD4, releasing its inhibitory state, and thereby regulates GOT2 proteasomal degradation by influencing K48-linked ubiquitination.

### The PLOD3-NEDD4-GOT2 axis mediates RCC malignant progression via glutamine metabolism reprogramming

**Fig. 7:**
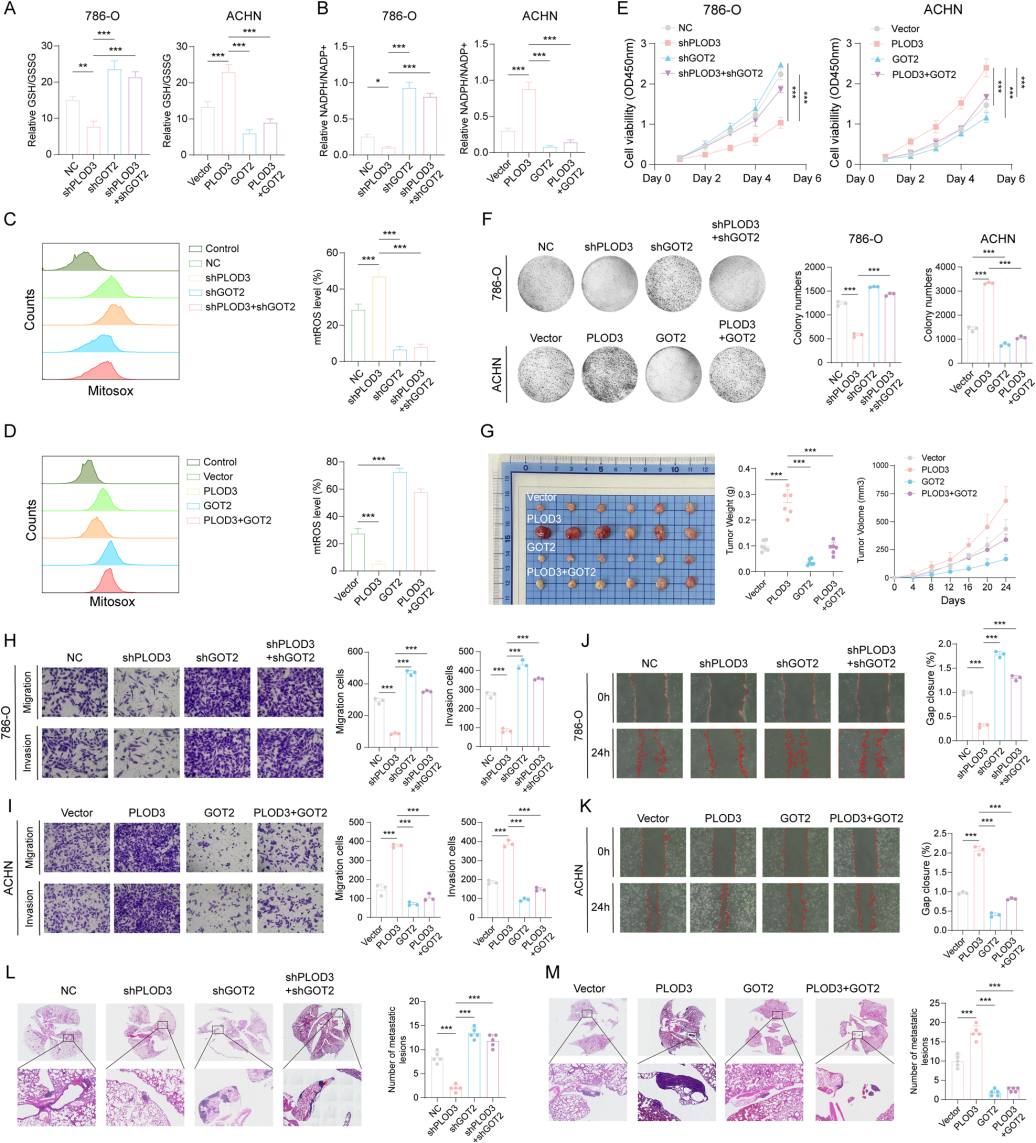
PLOD3 influences glutamine metabolism and RCC progression via a GOT2-dependent pathway. **A**, **B** GSH/GSSG and NADPH/NADP⁺ ratios in RCC cells following co-knockdown of PLOD3 and GOT2 and co-overexpression of PLOD3 and GOT2, along with statistical charts. **C**, **D** Mitochondrial ROS (Mitosox) levels in RCC cells following co-knockdown of PLOD3 and GOT2 and co-overexpression of PLOD3 and GOT2, along with statistical charts. CCK8 assay (**E**), plate clonogenic assay (**F**), tumor images and growth rate/weight statistics (**G**), Transwell assay (**H**, **I**), wound healing assay (**J**, **K**), and lung metastasis images (**L**, **M**) following co-knockdown of PLOD3 and GOT2 or co-overexpression of PLOD3 and GOT2 in RCC cells, along with statistical charts. *P < 0.05, **P < 0.01, ***P < 0.001.

GOT2, an important enzyme, has been shown to control the balance of a molecule called mtROS and play a role in tumor growth by changing how glutamine is processed [14]. We measured the amounts of GSH (reduced glutathione)/GSSG (oxidized glutathione) and NADPH/NADP+ in the cells. We also measured the activity of mtROS. This helped us understand how glutamine was being used in the cells and how mtROS levels were being controlled. Our data show that compared to the control group, the PLOD3 knockdown group had lower GSH/GSSG and NADPH/NADP+ ratios and higher mtROS activity. The PLOD3 and GOT2 double knockdown group restored the GOT2 knockdown-induced increases in GSH/GSSG and NADPH/NADP+ ratios while decreasing mtROS activity (Fig. 7A–D). Furthermore, we also measured the ratio of GSH/GSSG under hypoxic or after knockdown of HIF2α. The results showed that the ratio of GSH/GSSG significantly increased after hypoxic treatment compared to the control group, and silencing HIF2α could restore (Fig. S6D). Similarly, PLOD3 overexpression significantly elevated GSH/GSSG and NADPH/NADP+ ratios and markedly reduced α-KG and mtROS activity compared to controls (Fig. 7A–D). We further investigated whether the PLOD3-NEDD4-GOT2 axis influences RCC progression. CCK-8 and colony formation assays revealed that PLOD3 knockdown suppressed RCC cell proliferation and colony formation capacity, which was partially restored by shGOT2 transfection. Conversely, PLOD3 overexpression significantly enhanced RCC cell proliferation and colony formation, partially reversed by co-overexpression of GOT2 (Fig. 7E, F). Furthermore, in vivo validation using xenograft mouse models demonstrated that GOT2 knockdown significantly reduced subcutaneous tumor burden in nude mice, while dual overexpression of PLOD3 and GOT2 reversed the PLOD3-induced increase in subcutaneous tumor burden (Fig. 7G) In addition, we also measured the level of α-ketoglutarate (α-KG) in the tumor metabolites in vivo (Fig. S6E).

Notably, we further investigated the role of the PLOD3-NEDD4-GOT2 axis in lung metastasis. Wound healing and transwell assays demonstrated that PLOD3 knockdown reduced RCC cell migration and invasion, while dual knockdown of GOT2 and PLOD3 reversed the enhanced migration and invasion of RCC cells caused by GOT2 knockdown. Similarly, overexpression of GOT2 inhibited RCC cell migration and invasion, while dual overexpression of GOT2 and PLOD3 restored the migration and invasion-promoting effects mediated by PLOD3 overexpression (Fig. 7H–K). Furthermore, in a lung metastasis model established by tail vein injection of 786-O cells, GOT2 knockdown and PLOD3 overexpression significantly enhanced the ability of RCC cells to form metastatic lung lesions (Fig. 7L, M). Conversely, GOT2 overexpression and PLOD3 knockdown markedly reduced the number of lung metastatic foci formed by RCC cells, an effect that was restored by dual intervention.

**Fig. 8:**
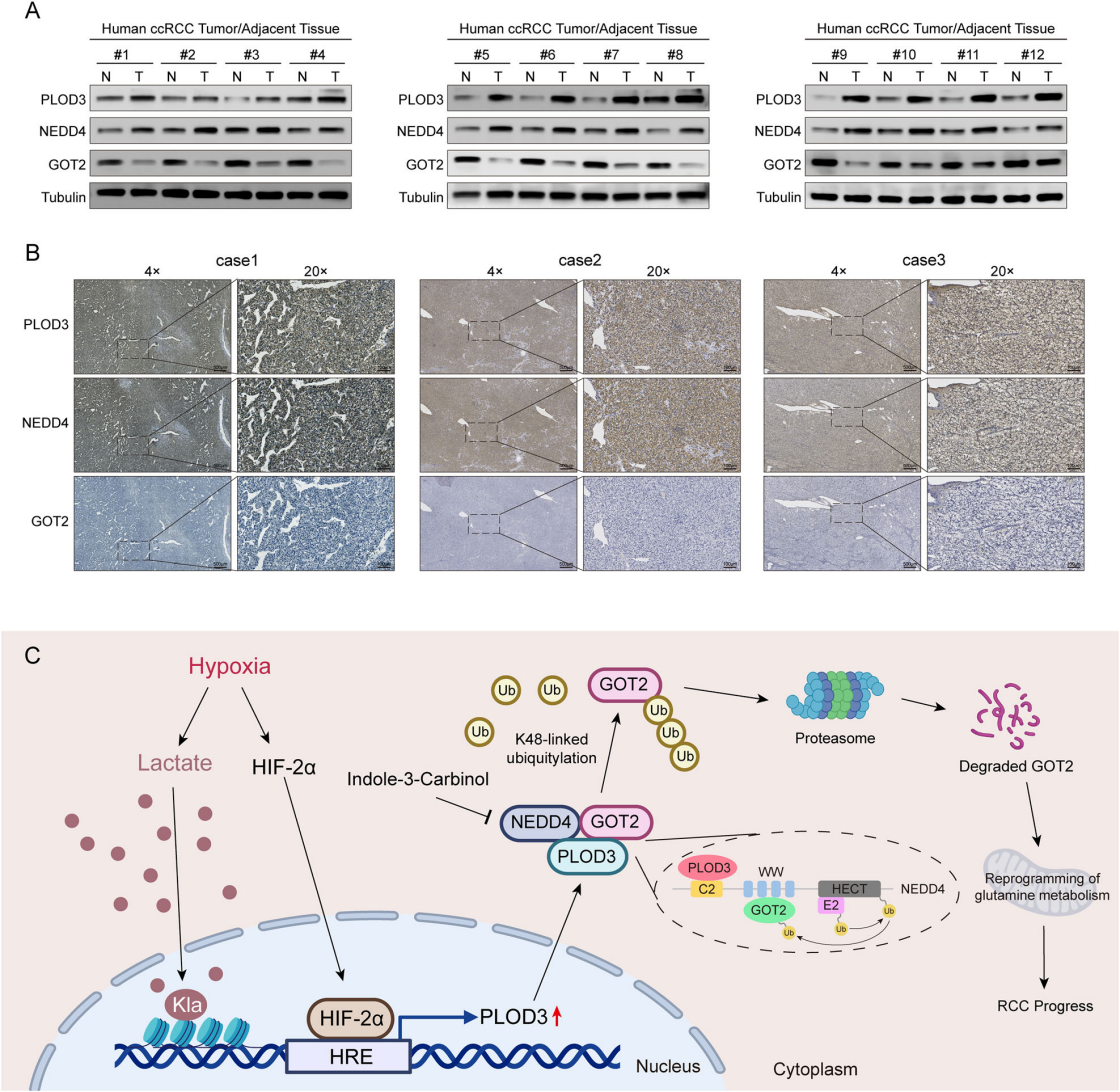
PLOD3/NEDD4/GOT2 ternary complex correlation and schematic diagram. **A** Immunoblotting analysis of relevant protein expression in ccRCC tumor tissue and adjacent normal tissue. **B** Representative IHC staining images and correlation line plots of PLOD3, NEDD4, and GOT2 expression in ccRCC tissue. **C** Schematic diagram of this study. Created with BioRender.com.

Finally, we validated the expression of the PLOD3-NEDD4-GOT2 axis in clinical tissue samples. As anticipated, PLOD3 and NEDD4 were specifically overexpressed in RCC patients compared to adjacent non-cancerous tissue, while GOT2 was specifically underexpressed in RCC patients. Furthermore, PLOD3 protein expression positively correlated with NEDD4 protein levels and both significantly negatively correlated with GOT2 expression (Fig. 8A, B). Collectively, these findings support the critical role of the PLOD3-NEDD4-GOT2 axis in mediating RCC progression, particularly proliferation and metastasis.

## Discussion

RCC accounts for approximately 90% of all renal cancer cases [24]. Approximately one-third of patients with RCC are diagnosed with advanced metastatic renal cell carcinoma at the initial presentation. Among the early stage patients who receive surgical treatment, 30–40% experience metastasis or postoperative recurrence [3]. Hypoxia is a key driver of malignant progression in RCC due to the VHL mutations in some ccRCCs. The rapid proliferation of renal cancer cells, combined with the uneven vascular distribution within tumors, leads to insufficient oxygen supply, creating a hypoxic microenvironment [25]. In this study, we obtained in vitro and in vivo evidence that PLOD3 functions as a hypoxic regulator, with oncogenic roles in RCC progression. Elevated PLOD3 expression levels predict poor prognosis in patients with RCC. Mechanistically, PLOD3 is abnormally overexpressed in RCC through HIF2α-mediated transcriptional activation, which is driven by the hypoxic tumor microenvironment and histone lactylation. We then determined the pathway through which PLOD3 produces these effects: PLOD3 mediates glutamine metabolic reprogramming through NEDD4 inhibition via GOT2 ubiquitination, influencing RCC proliferation and metastasis.

PLOD3 is a membrane-bound homodimeric enzyme that is primarily involved in collagen biosynthesis, which enhances tumor cell migration and invasion via increasing collagen deposition and crosslinking [11]. PLOD3 is highly expressed in certain solid tumors [26, 27] ; however, the role of PLOD3 in RCC malignant progression is not fully understood. We confirmed that PLOD3 is hypoxically regulated as a target gene of HIF2α. Furthermore, our multilevel analysis integrated big data, in *vitro* and in *vivo* experiments, and clinical tissue samples. We found that PLOD3 acts as an oncogene in RCC progression, promoting proliferation and metastasis. However, the mechanism of the tumorigenic role of PLOD3 remains unknown. PLOD3 interferes with the progression of colorectal cancer by affecting NF-κB nuclear translocation [26]. We identified the molecules downstream of PLOD3 via mass spectrometry and found that PLOD3, NEDD4, and GOT2 formed a ternary complex. We observed that PLOD3 specifically binds to the C2 domain of NEDD4, triggering NEDD4 to function as an E3 ubiquitin ligase. This modulates K48-linked ubiquitination, regulating GOT2 proteasomal degradation. We identified a pathway of the role of PLOD3 in tumors.

GOT2 is a prototypical moonlight enzyme that differs in function under different microenvironmental conditions through conformational changes or alterations in its subcellular localization. GOT2 is a key enzyme in the glutamine-aspartate shuttle pathway and participates in regulating the nitrogen balance and urea cycle metabolism. Li et al. demonstrated that GOT2 knockdown reprogrammed glutamine metabolism in hepatocellular carcinoma via enhancing glutamine degradation, nucleotide biosynthesis, and glutathione production. GOT2 thus maintained the redox balance and promoted tumor progression via the PI3K/AKT/mTOR signaling pathway [14]. Yang et al. reported that GOT2 participates in regulating pancreatic ductal adenocarcinoma cell senescence by controlling ROS levels and promoting tumor growth [28]. However, the expression and function of GOT2 in RCC remain unknown. We confirmed the specific downregulation of GOT2 expression in RCC. Furthermore, contrary to the findings of previous studies on inhibition, we found that GOT2 maintains mitochondrial ROS homeostasis through regulating glutamine metabolic reprogramming in RCC, promoting RCC proliferation and metastasis. This study fills a gap in the understanding of the role of GOT2 in RCC.

This study still has certain limitations. Although glutamine metabolism in renal cell carcinoma is gradually gaining attention, it is unlikely to function independently [19]. While this paper describes the role of the GOT2-glutamine metabolic axis in renal cell carcinoma, the specific mechanisms involved require further investigation. Further studies are required to fully elucidate how lactate-dependent pathways integrate with HIF-2α signaling. The present study has not determined whether these pathways act independently, additively, or synergistically, and this remains an important direction for future research. Additionally, we evaluated the biological behavior of renal cell carcinoma’s malignant progression by examining cellular proliferation and migration/invasion capabilities, without investigating PLOD3’s role in drug resistance to standard treatments (such as sunitinib). Moreover, this study focused on how PLOD3 affects mitochondrial reprogramming by regulating GOT2 stability, without directly examining the impact of PLOD3 on mitochondrial reprogramming itself. Therefore, more work is needed to explore novel mechanisms by which PLOD3 regulates mitochondrial reprogramming, as well as to identify inhibitors targeting PLOD3 and to develop new therapeutic strategies in combination with current RCC treatments. Moving forward, we will delve deeper into uncovering additional mechanisms by which PLOD3 functions as a potential therapeutic target for renal cell carcinoma.

In summary, our study provides evidence that PLOD3 is a target gene of HIF2α in hypoxic microenvironments and is a driver in NEDD4-mediated GOT2 ubiquitination and degradation. PLOD3 specifically binds to NEDD4, reverses NEDD4 autoinhibition, and influences GOT2 protein stability through K48-linked ubiquitination. PLOD3 thus participates in glutamine metabolic reprogramming and promotes RCC proliferation and metastasis. Our findings on the PLOD3/NEDD4/GOT2 regulatory axis in RCC provides insights into the mechanisms of RCC malignancy, identifying potential therapeutic targets for RCC.

## Conclusion

We found that PLOD3 is a target gene of HIF2α that regulates RCC growth and metastasis under hypoxic conditions via H3K18La induction. Furthermore, we identified the mechanism and specific domain through which PLOD3 triggers NEDD4 activation, promotes NEDD4 recruitment to GOT2, and interferes with GOT2 ubiquitination at K48, accelerating GOT2 degradation. Our findings collectively underscore the critical role of the PLOD3/NEDD4/GOT2 axis in RCC progression.

## Supplementary information

Supplementary Figures

Supplementary Tables

Original Data

## Data Availability

All data utilized in this study are included in this article and all data supporting the findings of this study are available on reasonable request from the corresponding author.
