# Supplementary material for: Hypoxia-induced PLOD3 activates the NEDD4/GOT2 axis to exacerbate renal cell carcinoma progression
Source: Cell Death Dis. 2026 Jun 10;17(1):735. doi: 10.1038/s41419-026-08961-1 (PMC13478339; doi:10.1038/s41419-026-08961-1)
Supplement: Supplementary file 1 [file 41419_2026_8961_MOESM1_ESM.pdf]

Supplementary Information for:

Hypoxia-induced PLOD3 activates the NEDD4/GOT2 axis to exacerbate renal cell carcinoma progression

Yan Xu[1,2#], Yu Lun[3#], Shiyue Wang[3#], Yu Jiao[4], Shikai Shen[3*], Yumeng Yan[5*], Xiaoyu Sun[6*]

Materials and methods

RNA sequencing and analysis

Total RNA was extracted using the Trizol reagent kit (Invitrogen, Carlsbad, CA, USA) according to the manufacturer's protocol. RNA quality was assessed using an Agilent 2100 Bioanalyzer (Agilent Technologies, Palo Alto, CA, USA) and verified by RNase-free agarose gel electrophoresis. Enriched mRNA was fragmented into short fragments using fragmentation buffer, then reverse transcribed into cDNA using random primers. Second-strand cDNA was synthesized using DNA polymerase I, RNase H, dNTPs (dUTP substituted for dTTP), and buffer. The cDNA fragments were then purified using the QiaQuick PCR Purification Kit (Qiagen, Venlo, The Netherlands), end-capped, poly(A)-tailed, and ligated to Illumina sequencing adapters. The second-strand cDNA was then digested with UNG (uracil-N-glycosylase). Digested products underwent size selection via agarose gel electrophoresis, PCR amplification, and sequencing by Genedino Biotechnology Co., Ltd. (Guangzhou, China) using an Illumina HiSeqTM 4000.

UALCAN database anaiysis

UALCAN is a comprehensive, user-friendly, and interactive web resource for analyzing cancer OMICS data. It is built on PERL-CGI with high quality graphics using javascript and CSS. UALCAN is designed to, a) provide easy access to publicly available cancer OMICS data (TCGA, MET500, CPTAC and CBTTC), b) allow users to identify biomarkers or to perform in silico validation of potential genes of interest, c) provide graphs and plots depicting expression profile and patient survival information for protein-coding, miRNA-coding and lincRNA-coding genes, d) evaluate epigenetic regulation of gene expression by promoter methylation, e) perform pan-cancer gene expression analysis, f) Provide additional information about the selected genes/targets by linking to GeneCards, Pubmed, TargetScan, The human protein atlas, DRUGBANK, Open Targets and the GTEx. These resources allow researchers to gather valuable information and data about the genes/targets of interest, g) provide clinical proteomic consortium data analysis including total/phospho-proteins and h) provide pediatric brain tumor gene expression and protein expression analysis.

Z-values represent standard deviations from the median across samples for the given cancer type. Log2 Spectral count ratio values from CPTAC were first normalized within each sample profile, then normalized across samples. **Statistics:** Unpaired two sample T-test was employed to evaluate levels of significance in expression level between any two sample groups

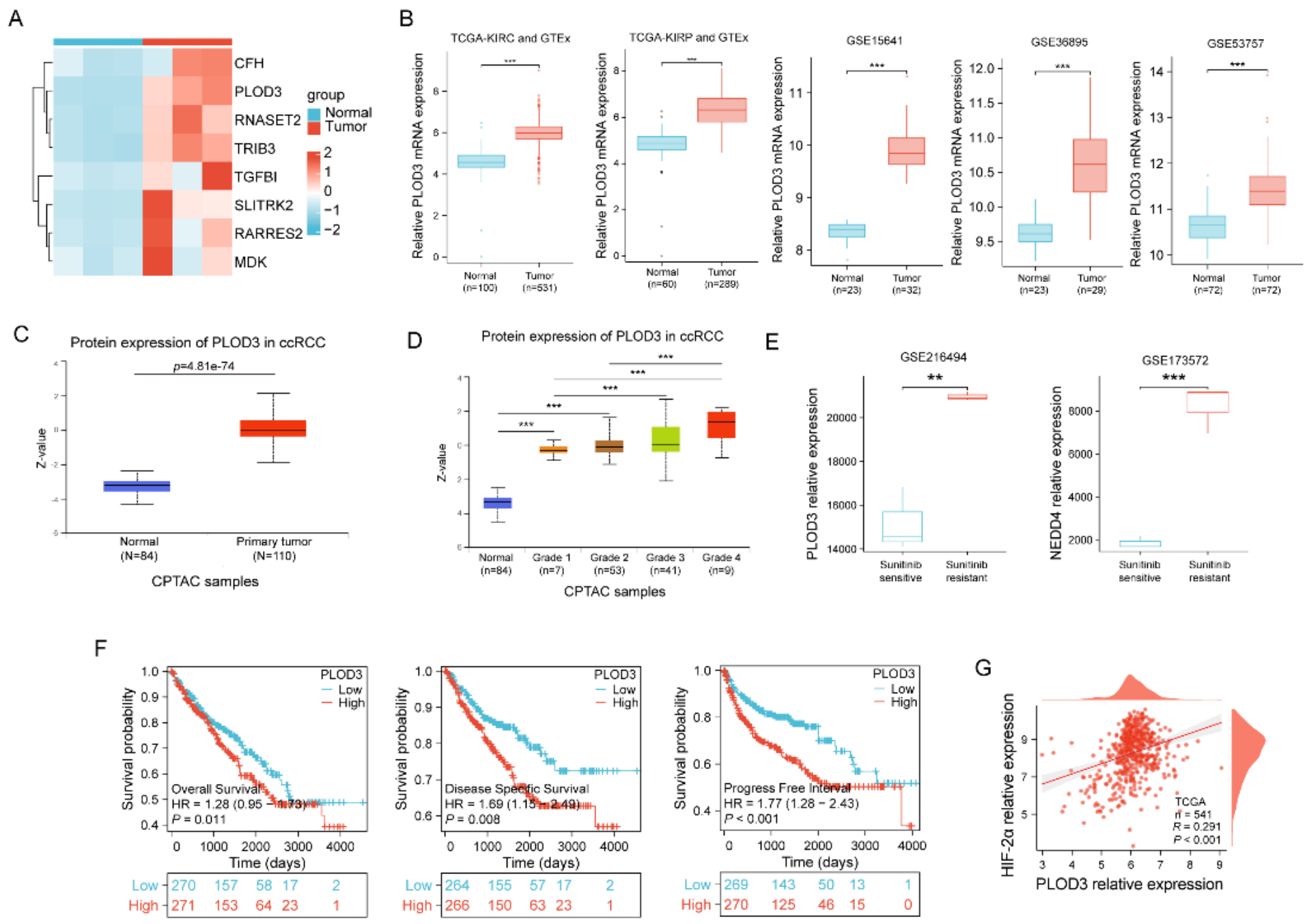

Figure S1 PLOD3 as a hypoxia-induced driver in ccRCC progression. (A) Heatmap of gene expression correlations in three pairs of ccRCC tumors and adjacent normal tissue samples. (B) Differences in PLOD3 mRNA expression levels between RCC and adjacent normal tissue in the TCGA and GEO databases. (C) Differences in PLOD3 protein expression levels between ccRCC and adjacent normal tissue in the CPTAC database from UALCAN database anaiysis. (D) Differences in PLOD3 protein expression across different grades of ccRCC in the CPTAC database. (E) Bioinformatics analysis of PLOD3 and NEDD4 differences in sunitinib sensitivity and resistance. (F) Survival curves for Overall Survival (OS),Disease-Specific Survival (DSS), and Progression-Free Interval (PFI) in ccRCC patients with high/low PLOD3 expression, analyzed using the TCGA database. (G) Correlation between PLOD3 and HIF-2α mRNA expression levels in the TCGA database.

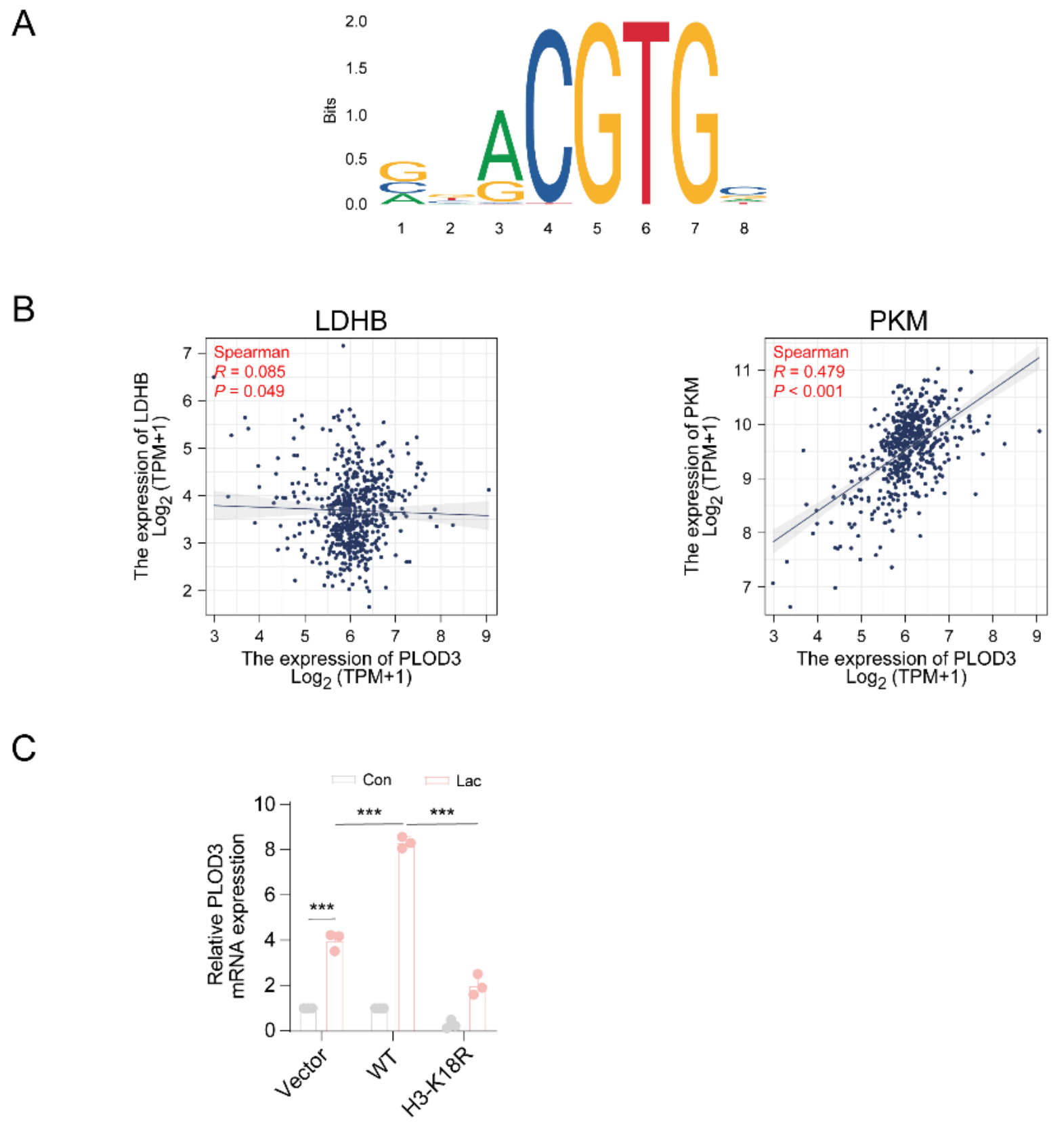

Figure S2 Hypoxia drives PLOD3 expression in RCC. (A) HIF-2α binding sites identified from the JASPAR database. (B) Correlation between PLOD3 and HK2, LDHB, and PKM2 mRNA expression levels in the TCGA database. (C) Expression levels of PLOD3 after histone methylation site mutations.

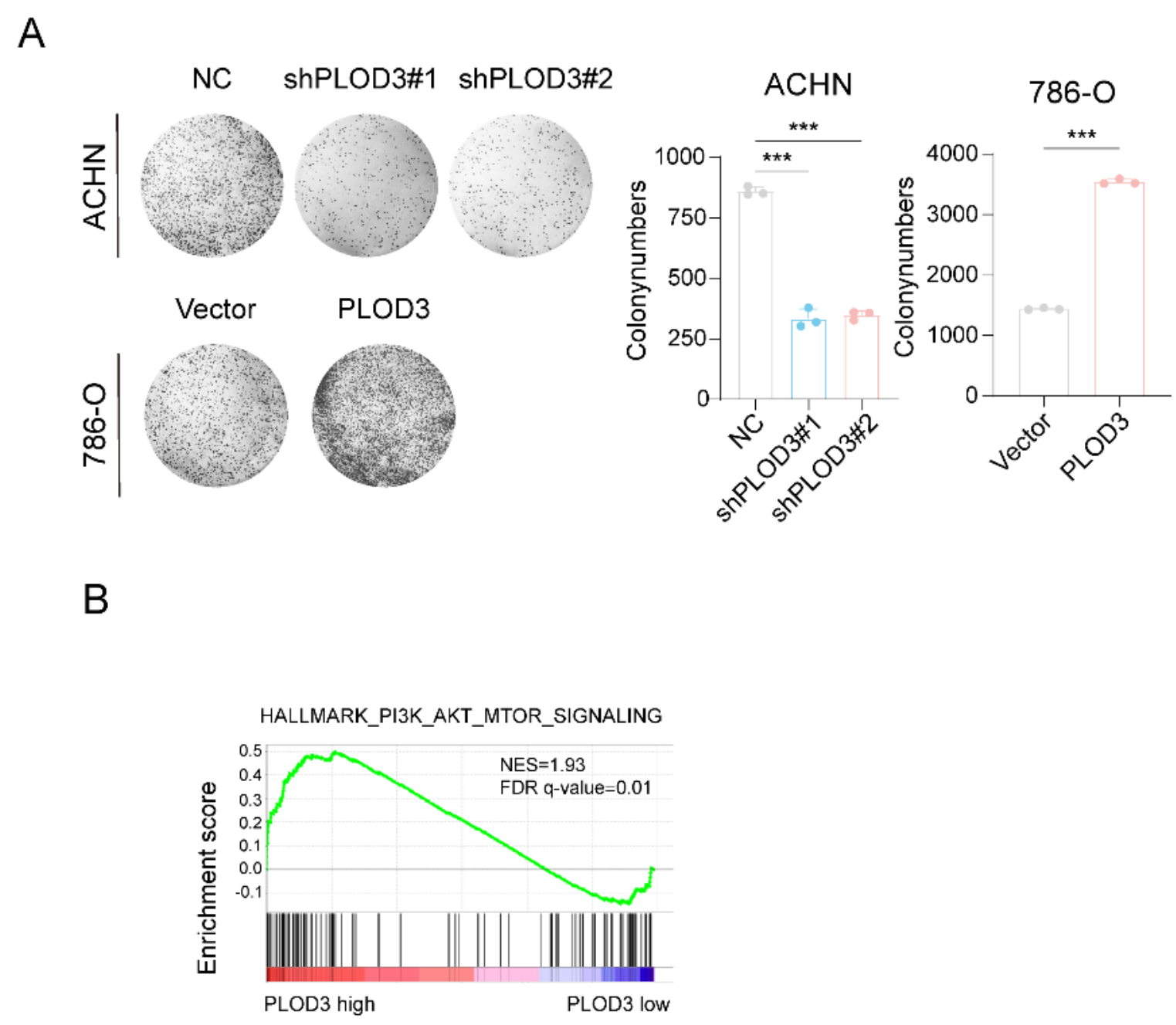

Figure S3 PLOD3 promotes RCC cell growth and metastasis in vivo and in vitro. (A) Plate-clone assays and statistical charts for PLOD3 knockdown and overexpression in ACHN and 786-O cells. (B) PI3K/AKT/mTOR signaling pathway enrichment analysis of PLOD3 high- and low-expression groups in the TCGA database.

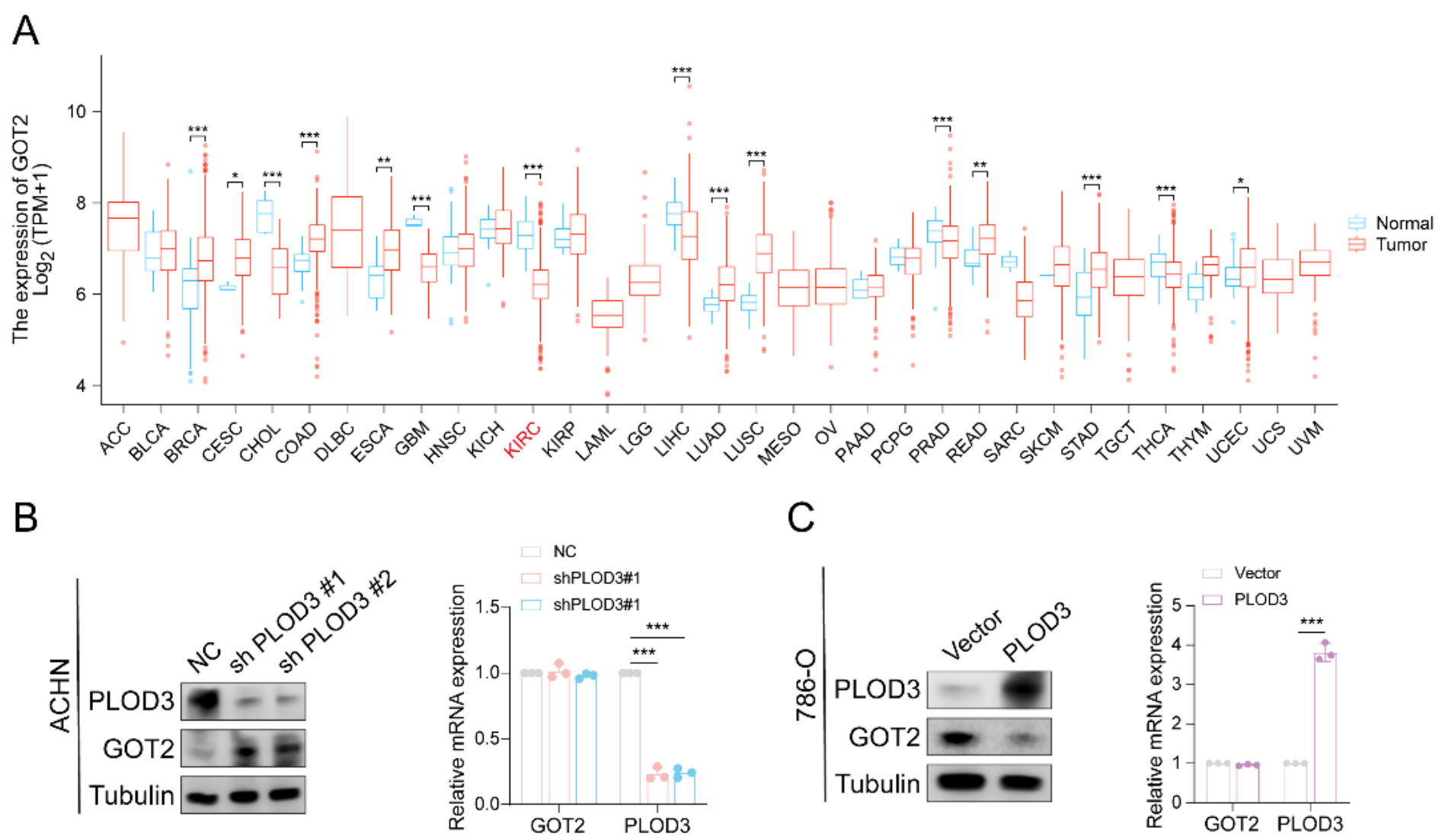

Figure S4 PLOD3 forms protein complexes with NEDD4 and GOT2. (A) GOT2 mRNA

expression level in pan-cancer. (B) Protein expression levels of PLOD3 and GOT2 in ACHN cells after PLOD3 knockdown. (C) Protein expression levels of PLOD3 and GOT2 in 786-O cells after PLOD3 overexpression.

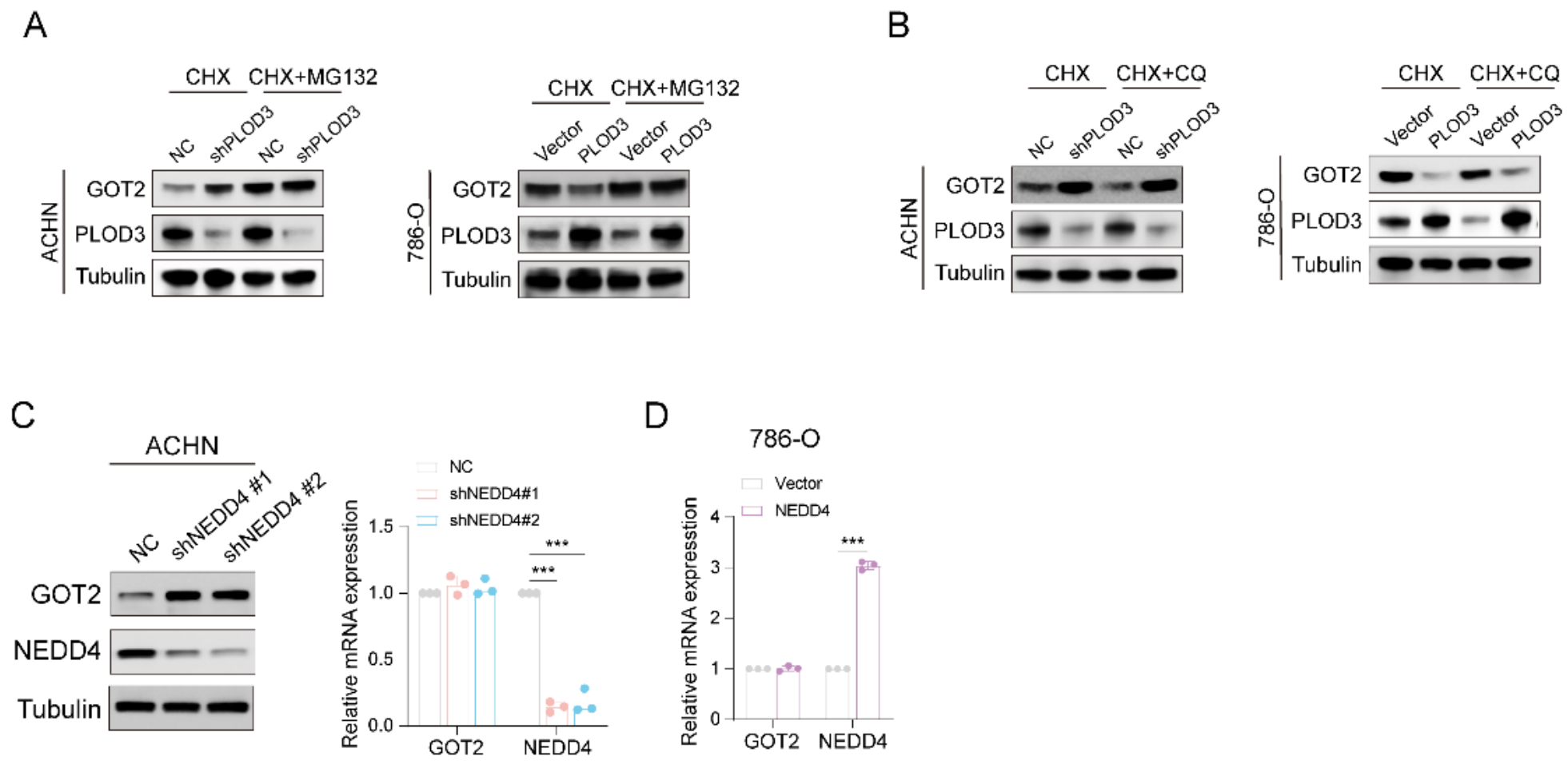

Figure S5 PLOD3 and NEDD4 influence GOT2 stability. (A) GOT2 protein expression after CHX or MG132 treatment following PLOD3 knockdown and overexpression. (B) GOT2 protein expression after CHX or CQ treatment following PLOD3 knockdown and overexpression. (C) Target protein expression levels in ACHN cells after NEDD4 knockdown. (D) mRNA expression levels of target proteins in 786-O cells after NEDD4 overexpression.

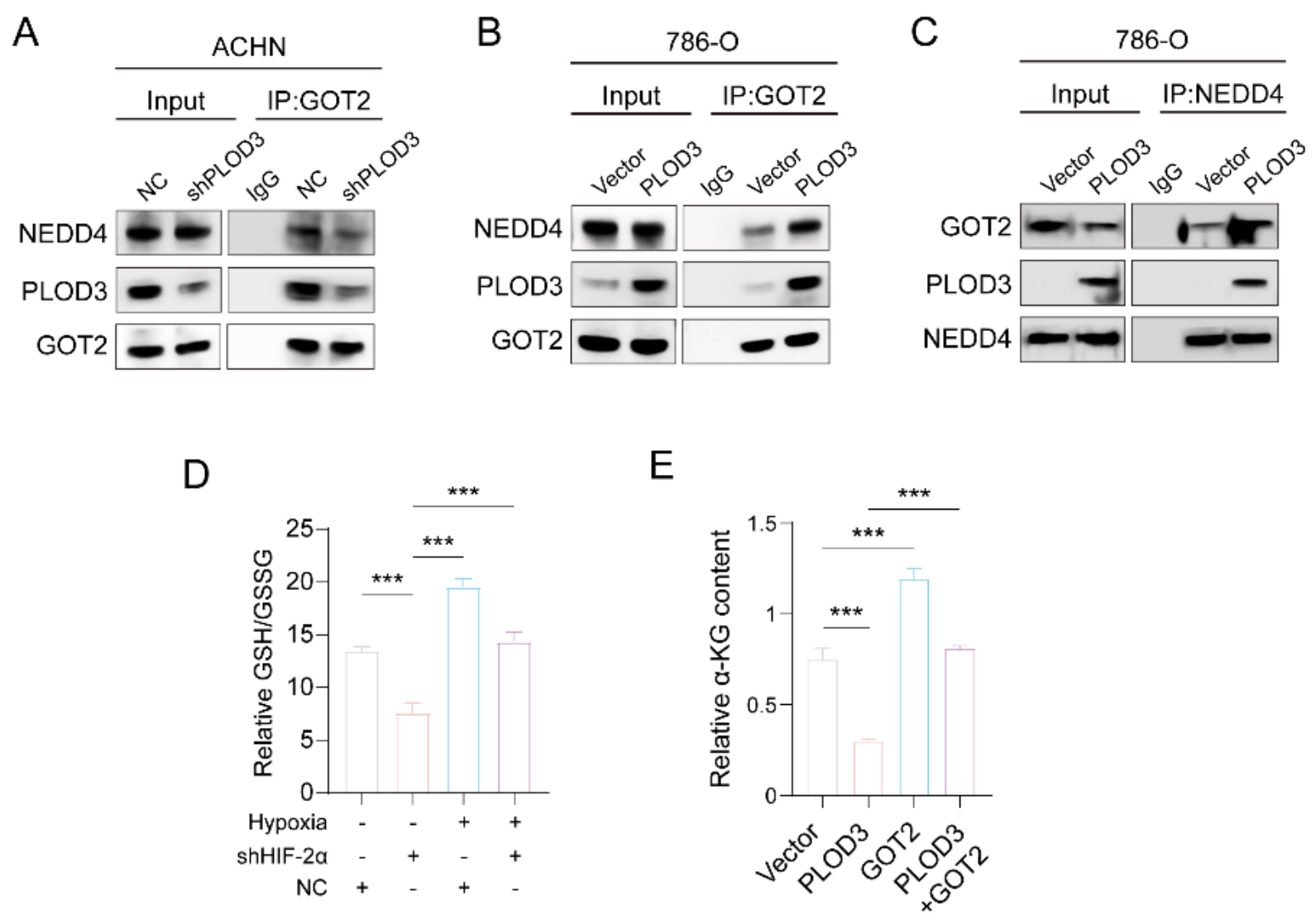

Figure S6 PLOD3 promotes NEDD4-GOT2 interaction to degrade GOT2 protein. (A) Changes in NEDD4-GOT2 binding following PLOD3 knockdown, validated by GOT2 as the IP group control. (B) Changes in NEDD4-GOT2 binding following PLOD3 overexpression, validated by GOT2 as the IP group control. (C) Changes in NEDD4-GOT2 interaction following PLOD3 overexpression, validated by NEDD4 as the IP co-precipitant, compared to Input control. (D) GSH/GSSG ratios in RCC cells following hypoxia and knockdown of HIF-2α. (E) The level of α-ketoglutarate (α-KG) in the tumor metabolites in vivo.
