# Supplementary material for: Hypoxia-induced PLOD3 activates the NEDD4/GOT2 axis to exacerbate renal cell carcinoma progression
Source: Cell Death Dis. 2026 Jun 10;17(1):735. doi: 10.1038/s41419-026-08961-1 (PMC13478339; doi:10.1038/s41419-026-08961-1)
Supplement: Supplementary file 2 [file 41419_2026_8961_MOESM2_ESM.pdf]

Table S1. The antibodies used in this study.

Antibodies	SOURCE	IDENTIFIER	Host	Dilution
PLOD3	Proteintech	Cat#11027-1-AP	Rabbit	1：10000
HIF-2α	Cell Signaling Technology	Cat#7096	Rabbit	1：1000
NEDD4	Proteintech	Cat#21698-1-AP	Rabbit	1：6000
GOT2	Proteintech	Cat#14800-1-AP	Rabbit	1：10000
Flag	Proteintech	Cat#66008-4-Ig	Mouse	1：30000
HA	Proteintech	Cat#51064-2-AP	Rabbit	1：10000
Myc	Cell Signaling Technology	Cat#2276	Mouse	1：1000
Histone H3	Cell Signaling Technology	Cat#14269	Rabbit	1：1000
Ub	Cell Signaling Technology	Cat#43124	Rabbit	1：1000
Actin	Proteintech	Cat#20536-1-AP	Mouse	1：8000
Tubulin	Proteintech	Cat#10094-1-AP	Mouse	1：10000

Table S2. The sequences of qRT-PCR primers

Target gene	Primer forward sequence (5'-3')	Primer reverse sequence (5'-3')
HIF-2α	CTACGCCACCCAGTACCAGG	GACACCTTGTGGGCTGACG
β-actin	TCCTCCCTGGAGAAGAGCTA	TCCTGCTTGCTGATCCACAT
PLOD3	CAGAGATGGAGCACTACGGC	CTTGGTGTGGTAACCGGGAA
GOT2	AAGAGGGACACCAATAGCAAAAA	GCAGAACGTAAGGCTTTCCAT
NEDD4	CACAAGCCTCCACCAGTGAT	TCCTCTCAGATGGGCTGGAA

Table S3. Targeted sequences of shRNAs used in this study.

RNAi	RNAi sequence
sh-HIF-2α	GTTCTGGTGACTCTTGGTC
shPLOD3-1	GGGAGGATATGATCATCATGT
shPLOD3-2	GGCCTTCTGTAAGAGCTTTCG
shNEDD4-1	CGCCTTGACTTACCTCCATAT
shNEDD4-2	GCGGATGAGAATAGAGAACTT
