# Supplementary material for: Hypoxia-induced PLOD3 activates the NEDD4/GOT2 axis to exacerbate renal cell carcinoma progression
Source: Cell Death Dis. 2026 Jun 10;17(1):735. doi: 10.1038/s41419-026-08961-1 (PMC13478339; doi:10.1038/s41419-026-08961-1)

Figure 1E

PLOD3

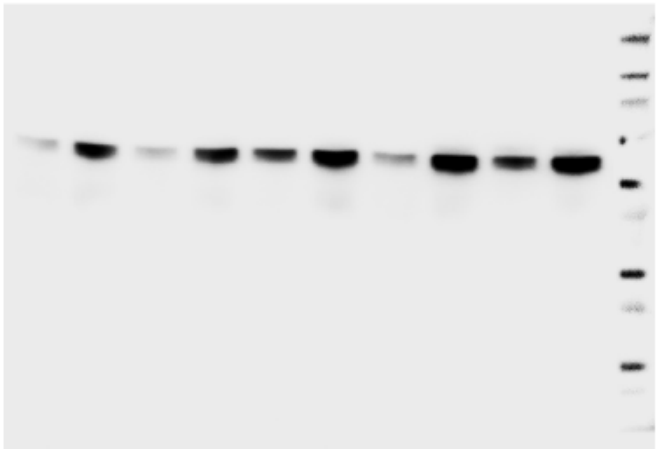

Tubulin

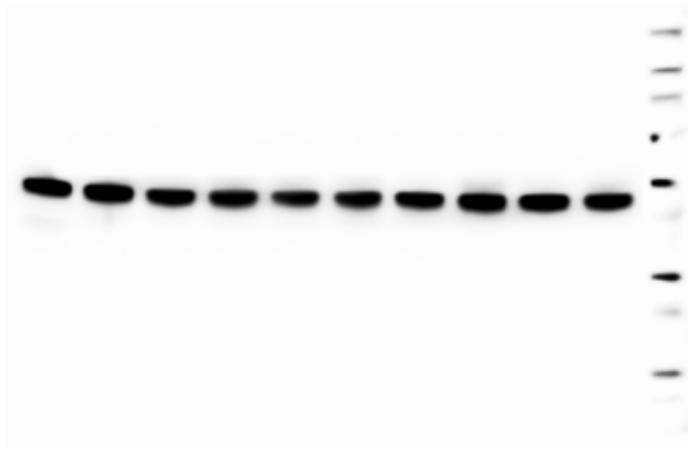

Figure 1E

PLOD3

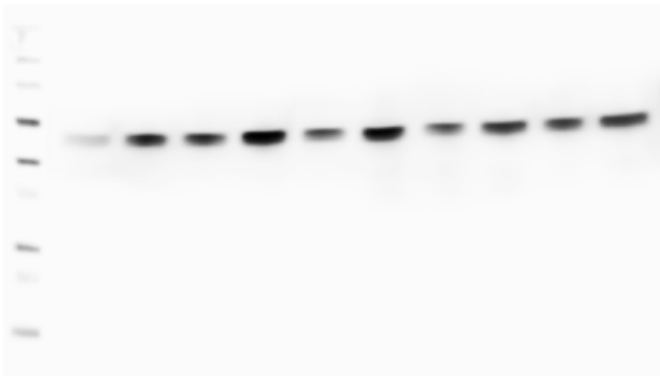

Tubulin

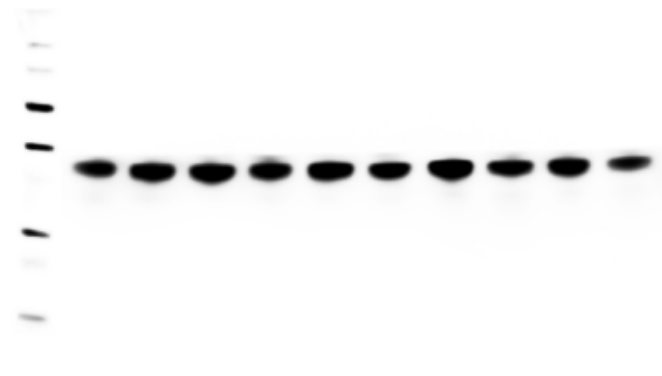

Figure 1E

PLOD3

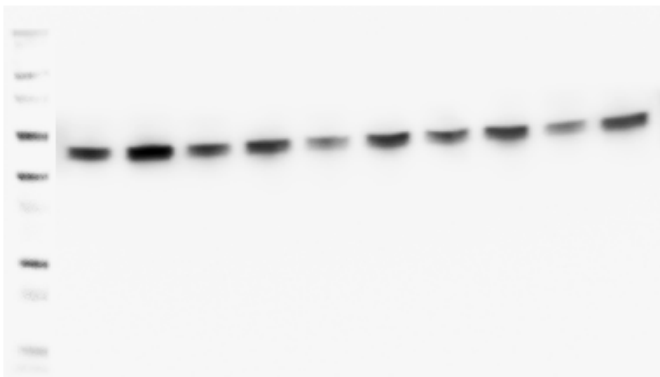

Tubulin

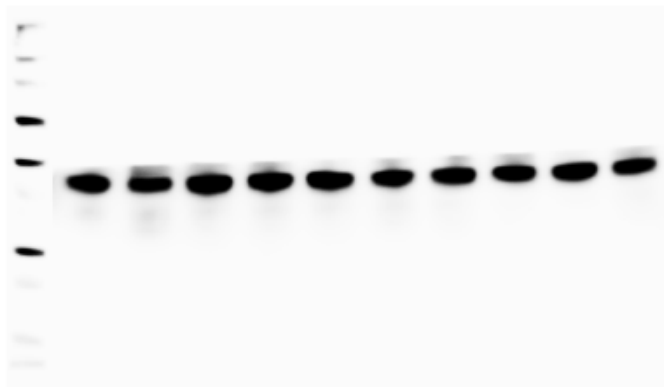

Figure 1E

PLOD3

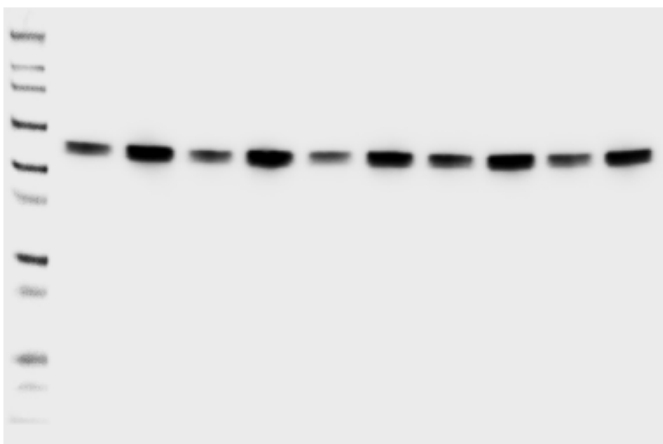

Tubulin

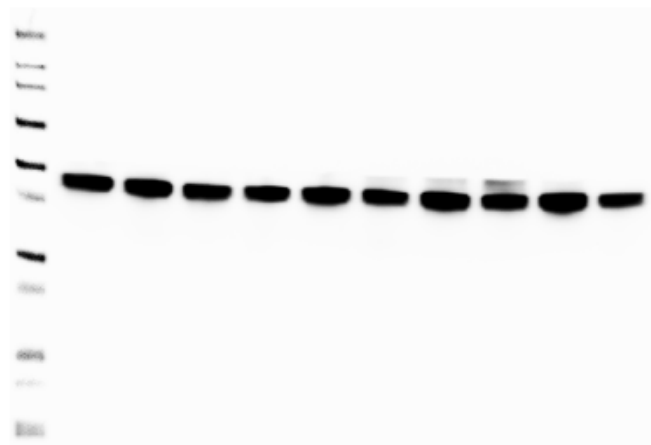

Figure 1G

PLOD3

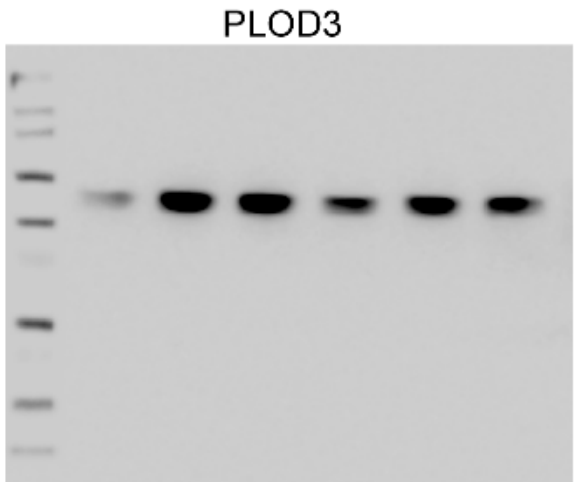

Tubulin

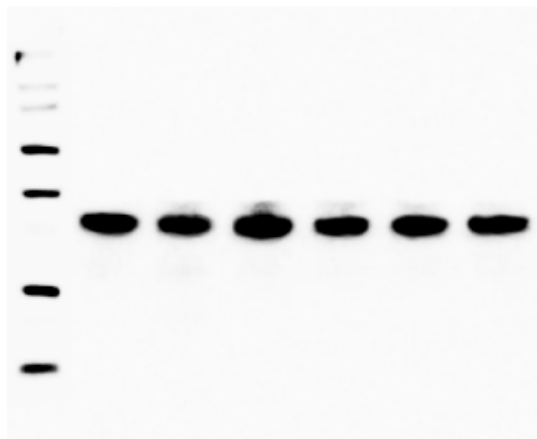

Figure 2B

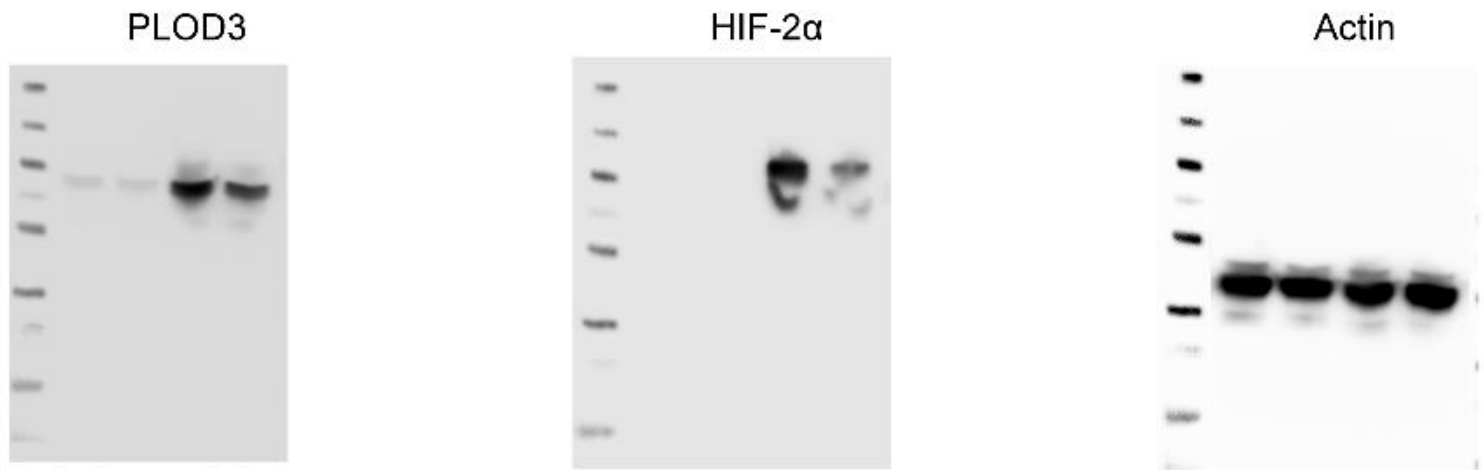

Figure 2D

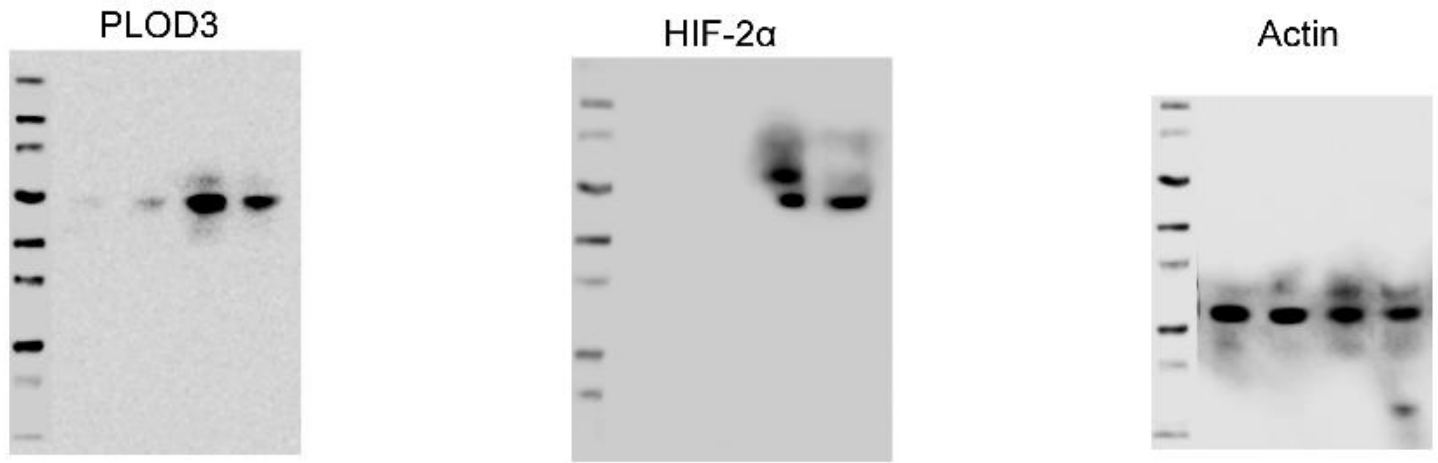

Figure 2F

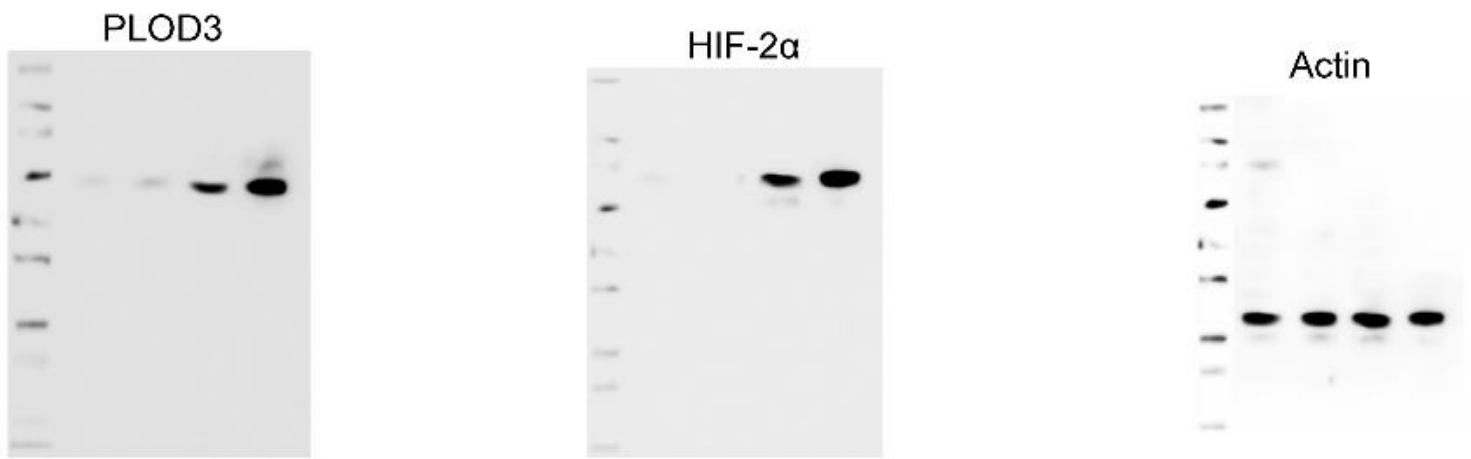

Figure 2L

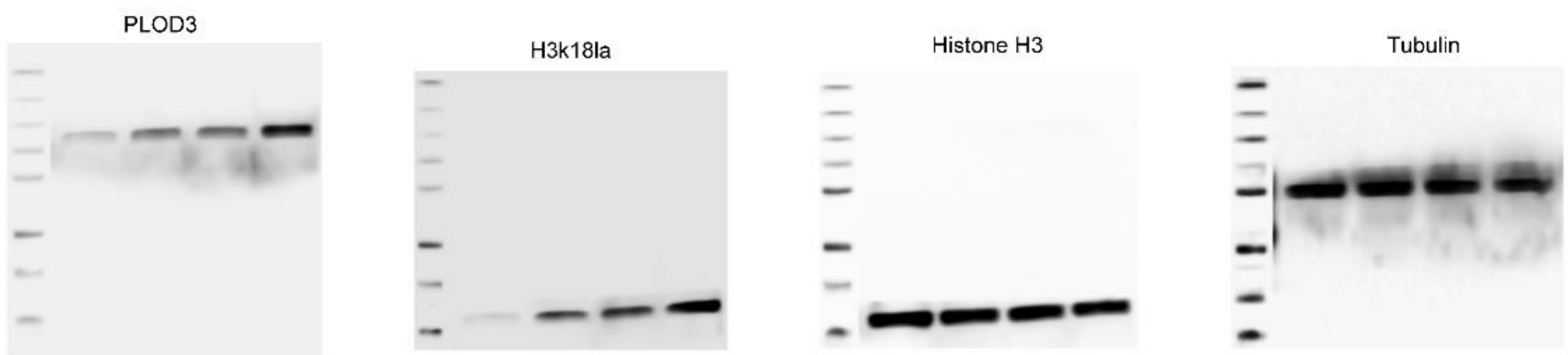

Figure 2M

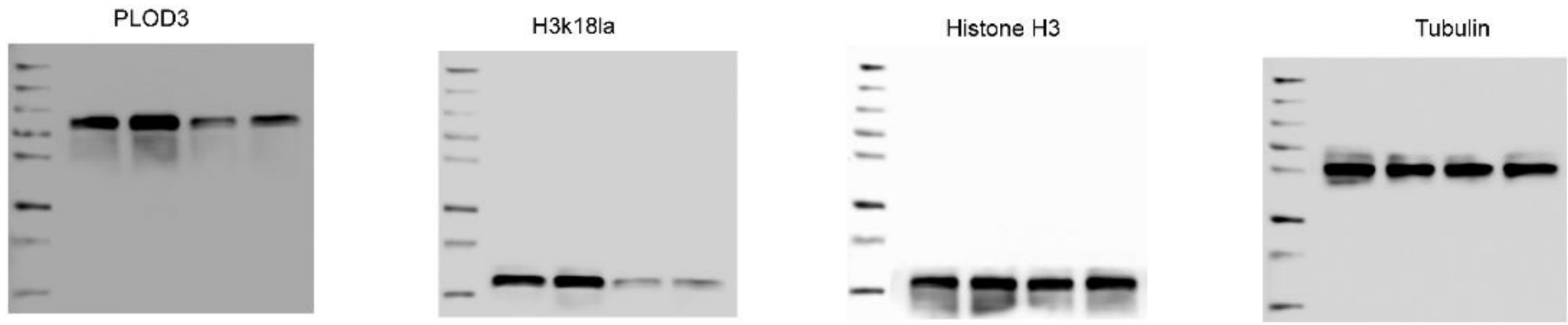

Figure 2N

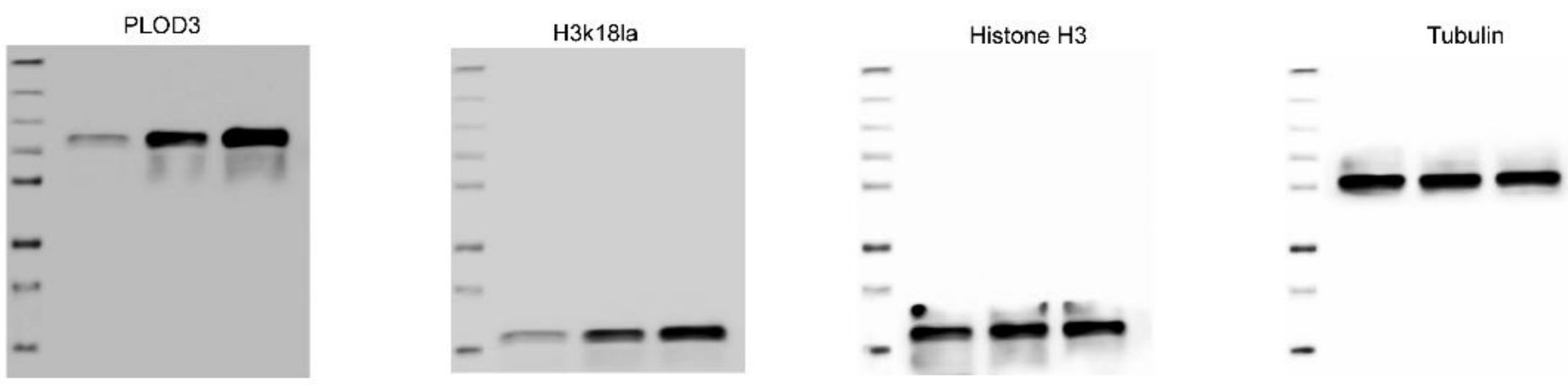

Figure 3A

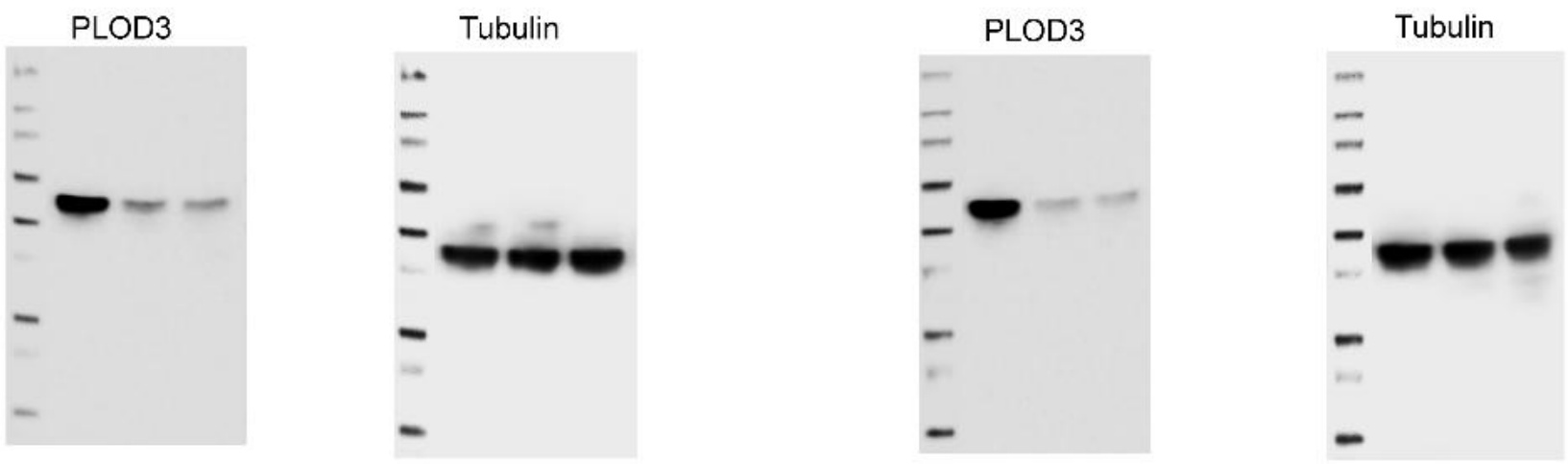

Figure 3C

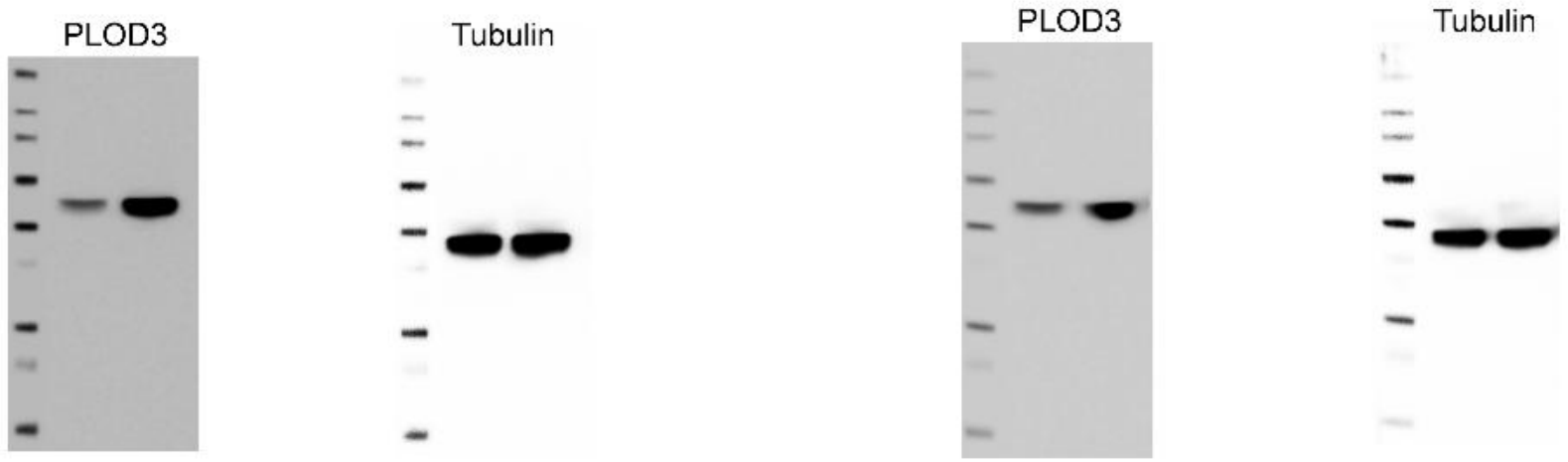

Figure 3N

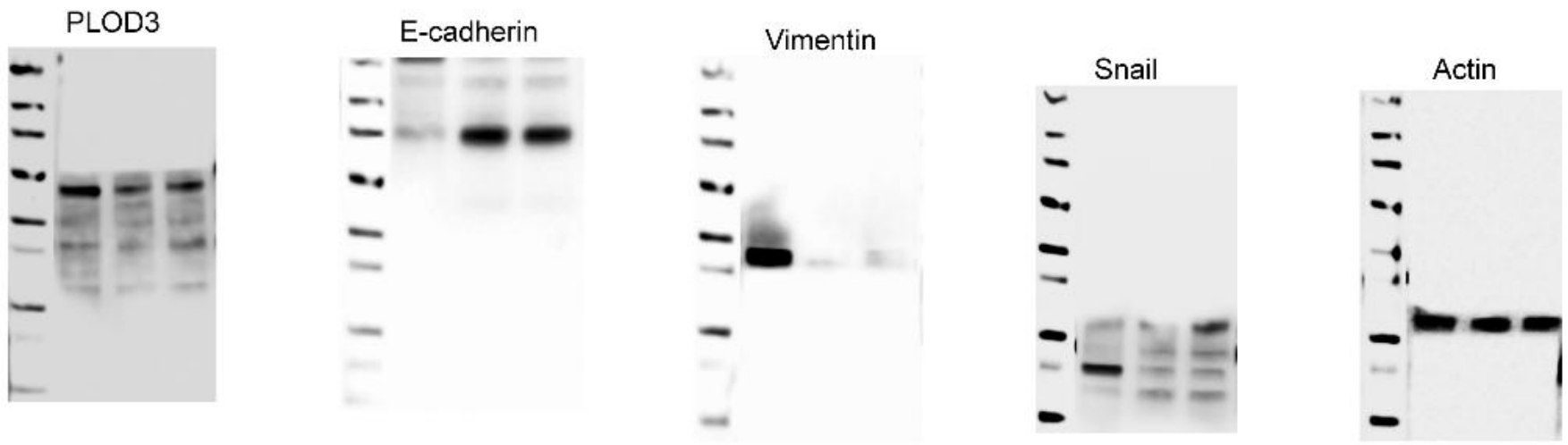

Figure 4C

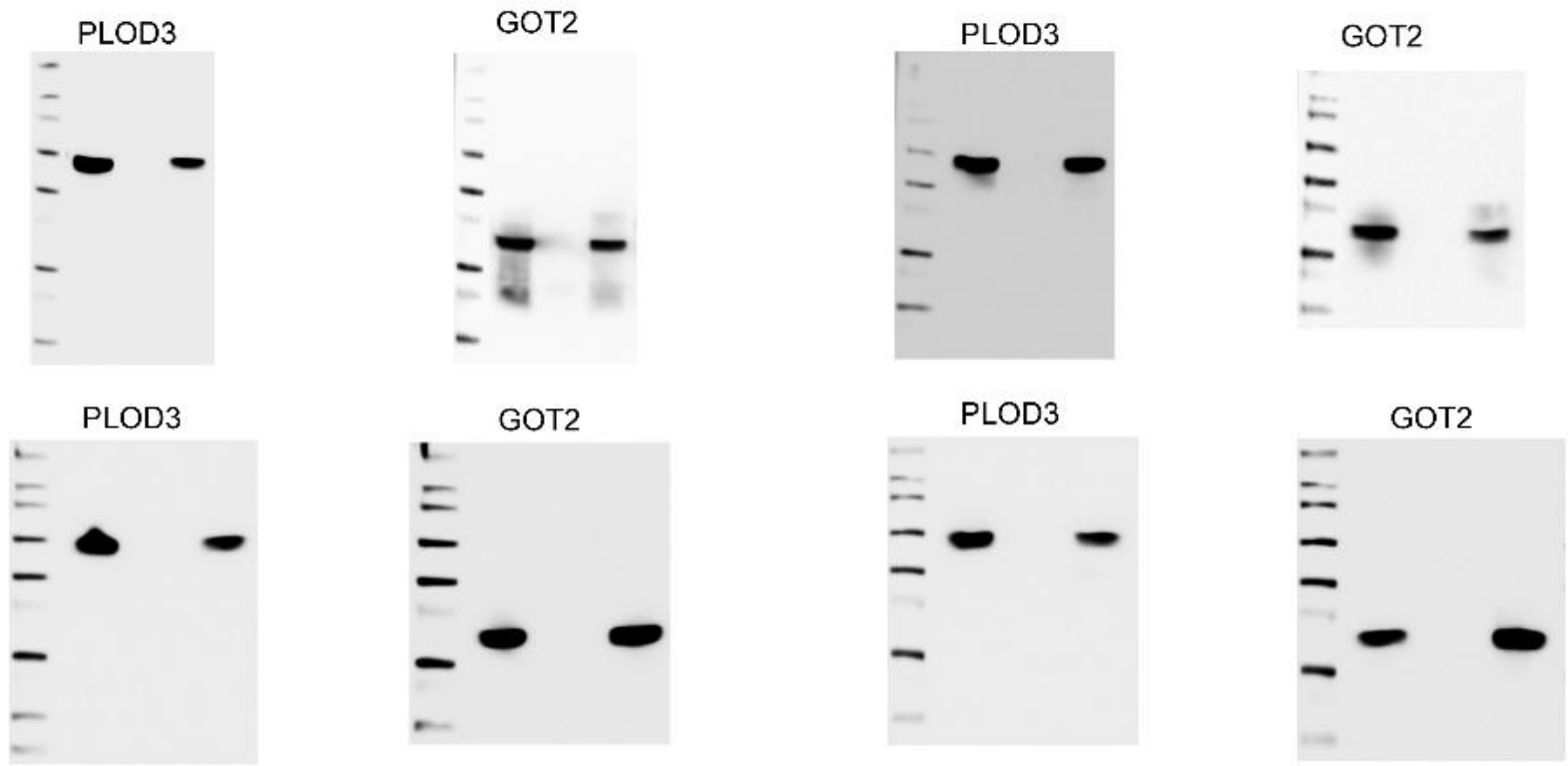

Figure 4E

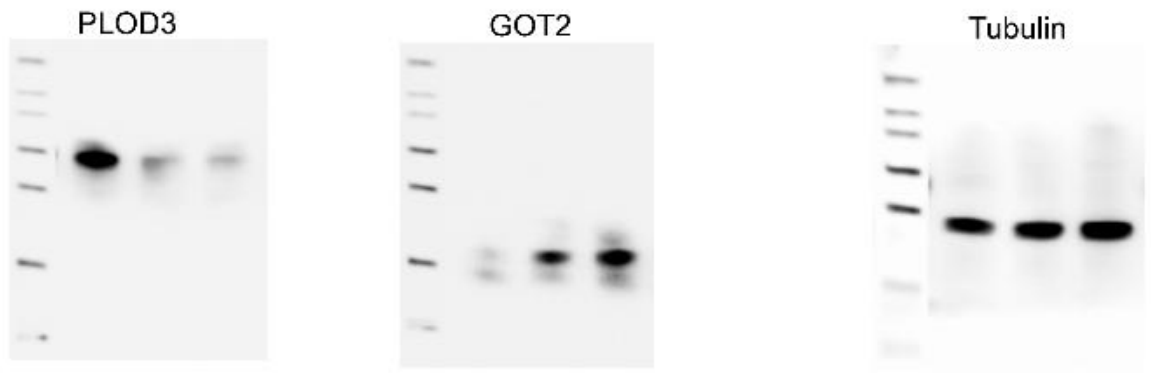

Figure 4F

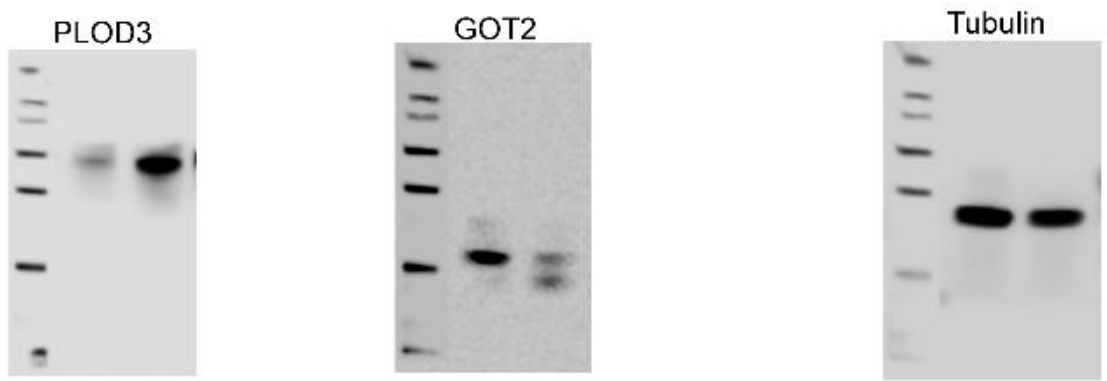

Figure 4K

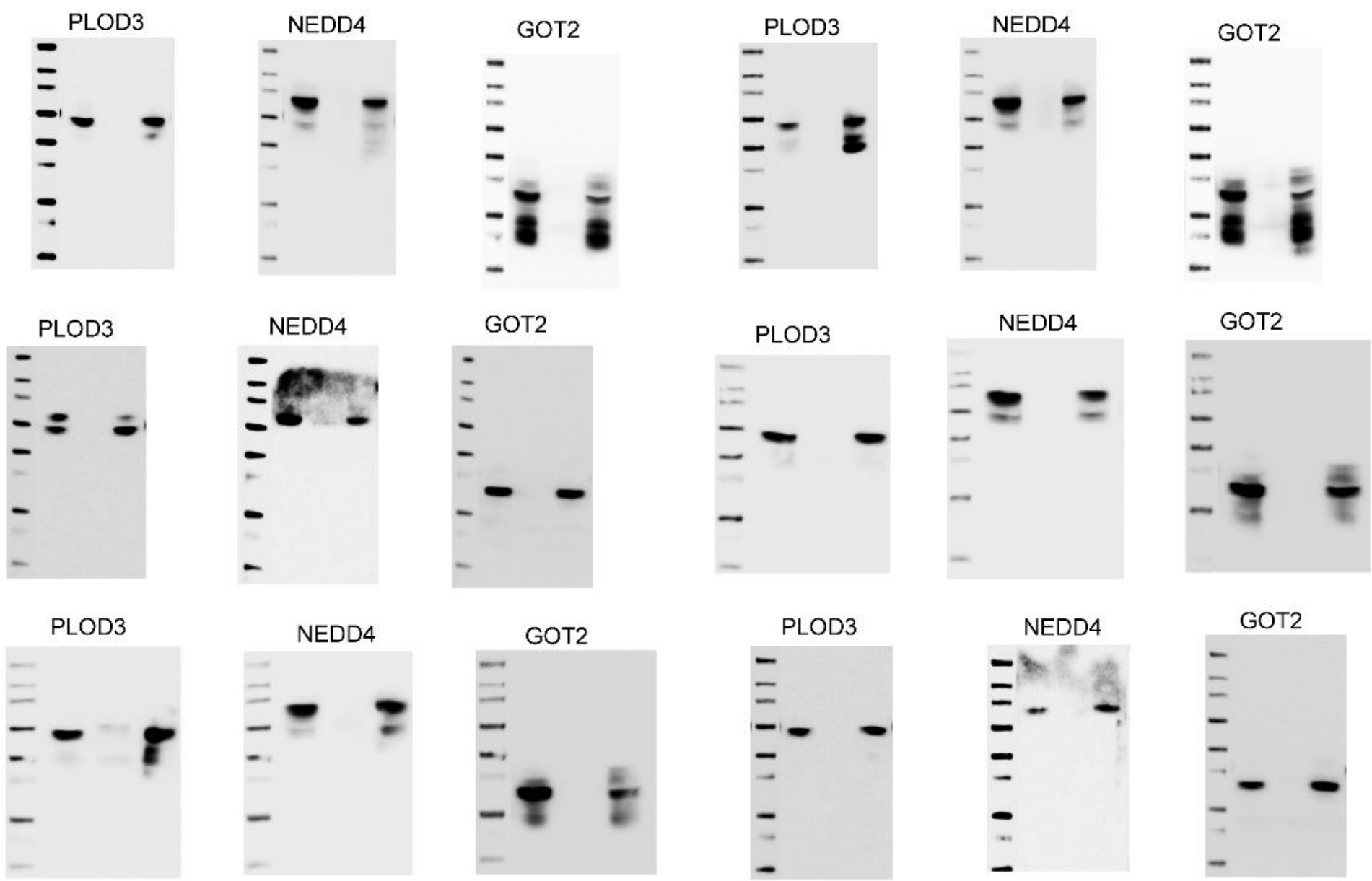

Figure 4N

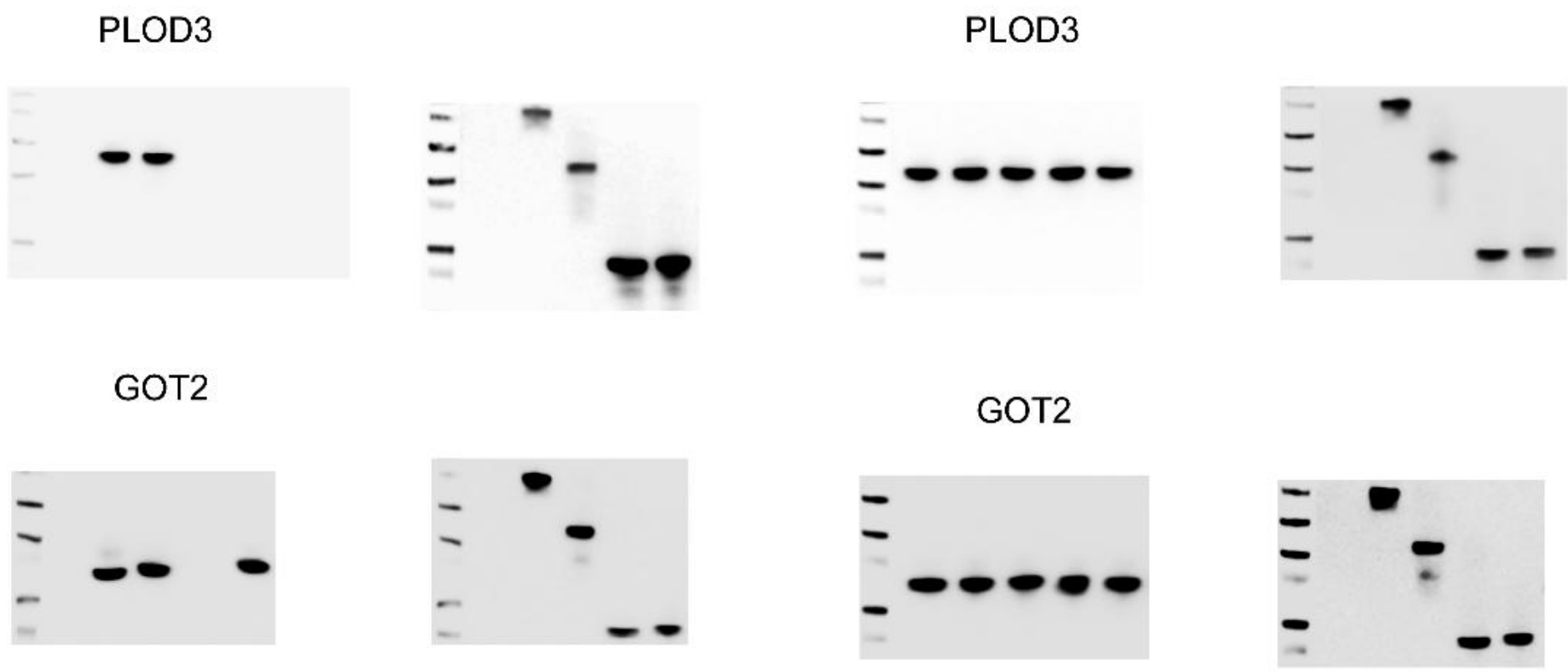

Figure 5A

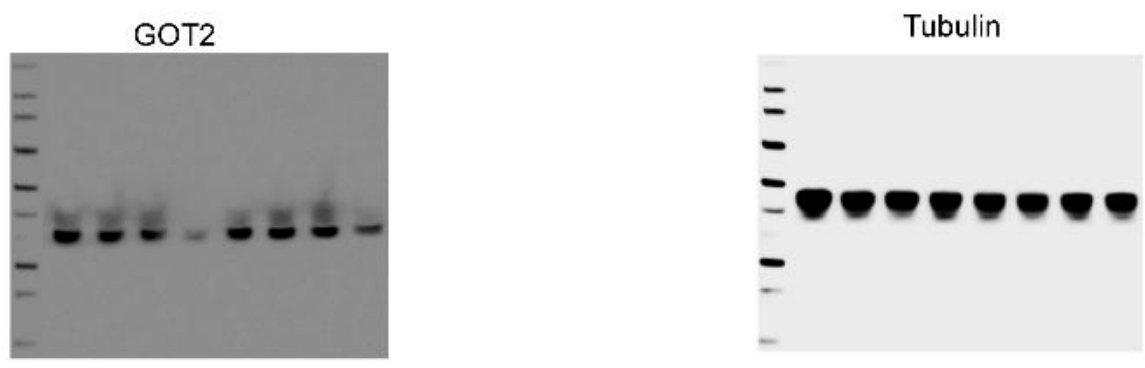

Figure 5B

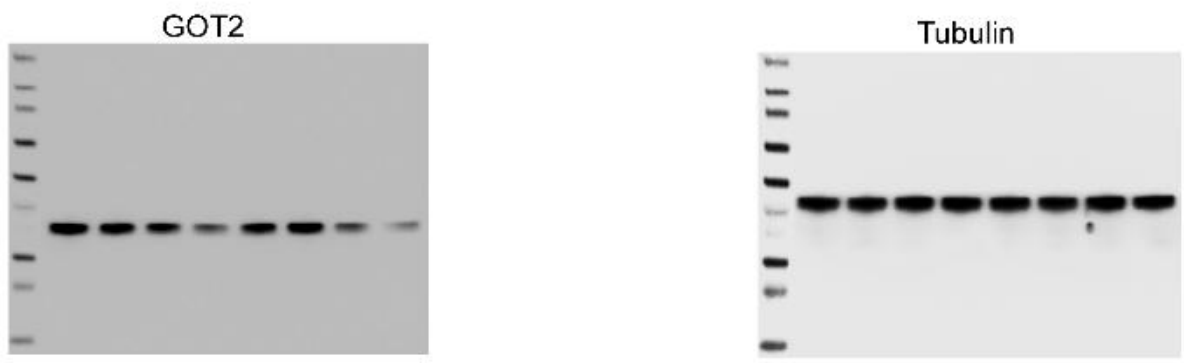

Figure 5C

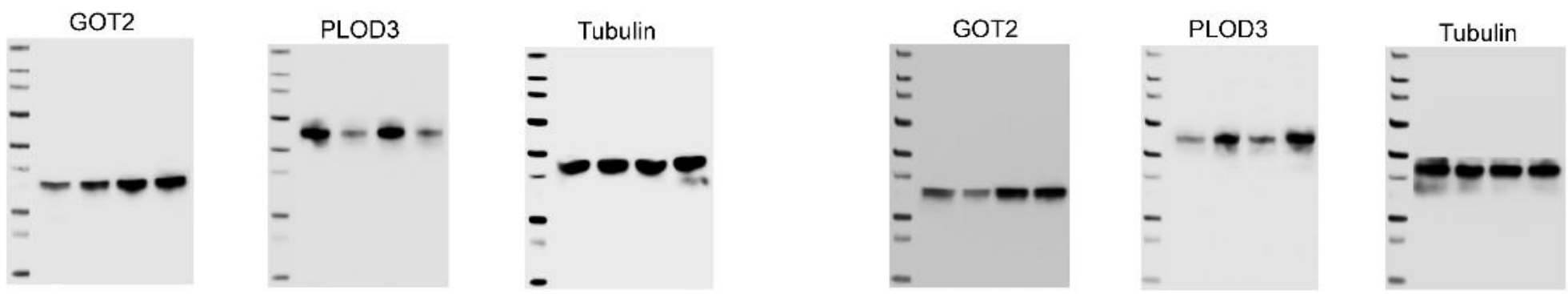

Figure 5D

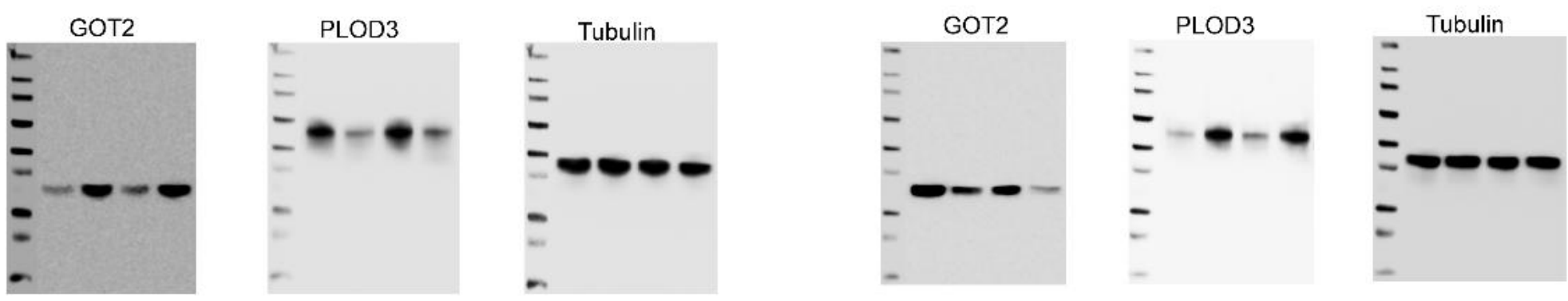

Figure 5E

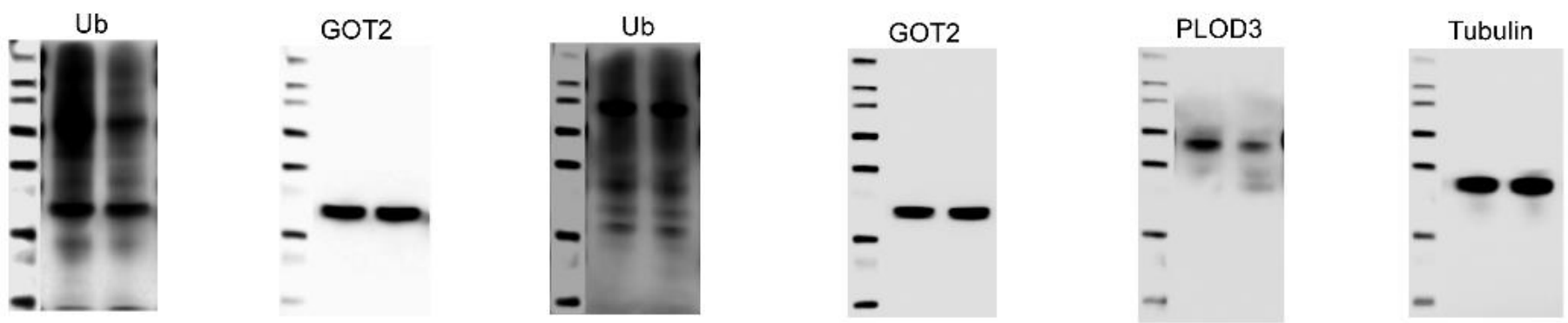

Figure 5F

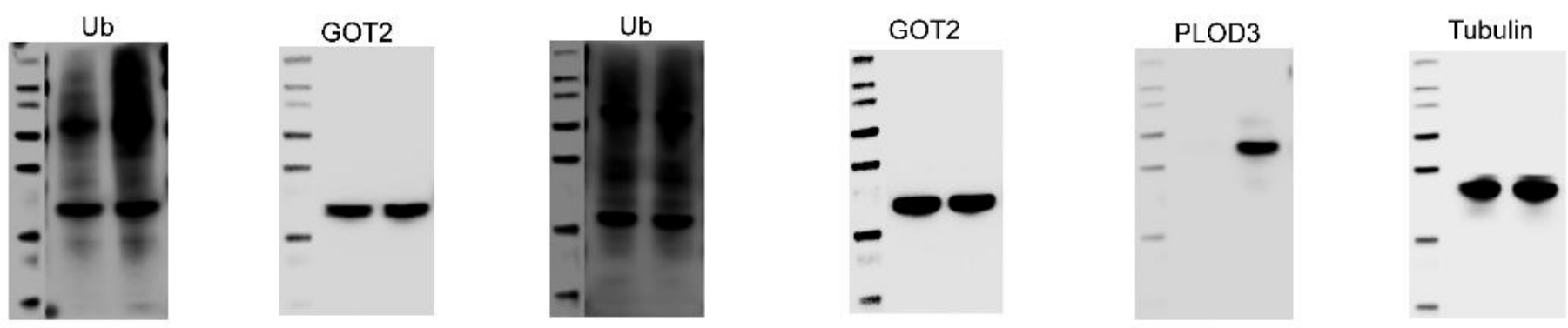

Figure 5G

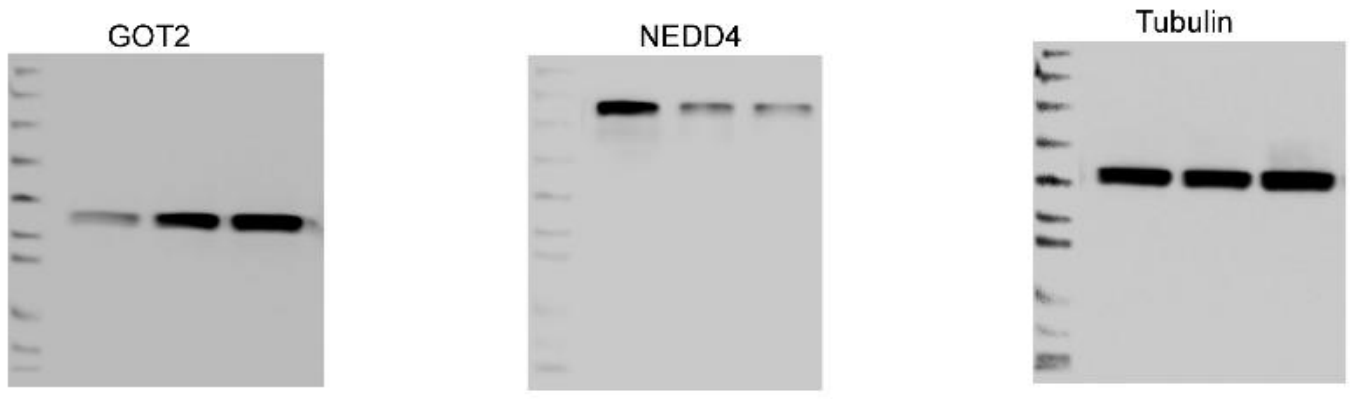

Figure 5H

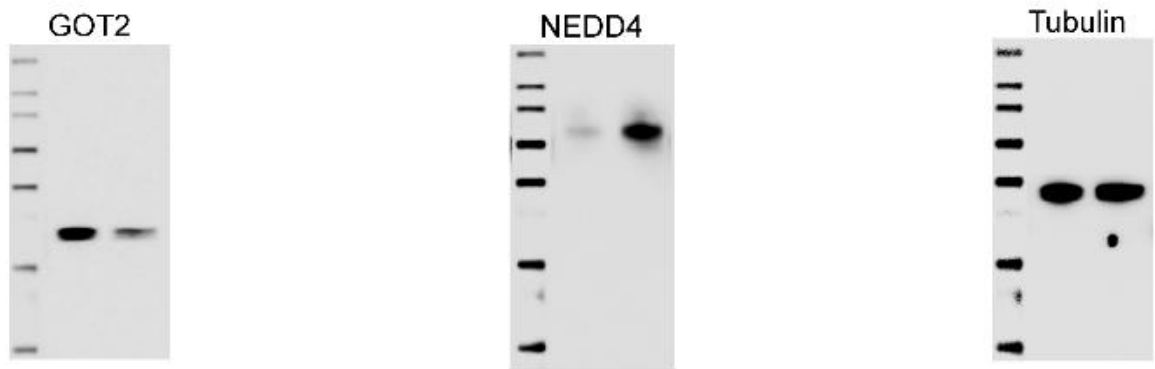

Figure 5I

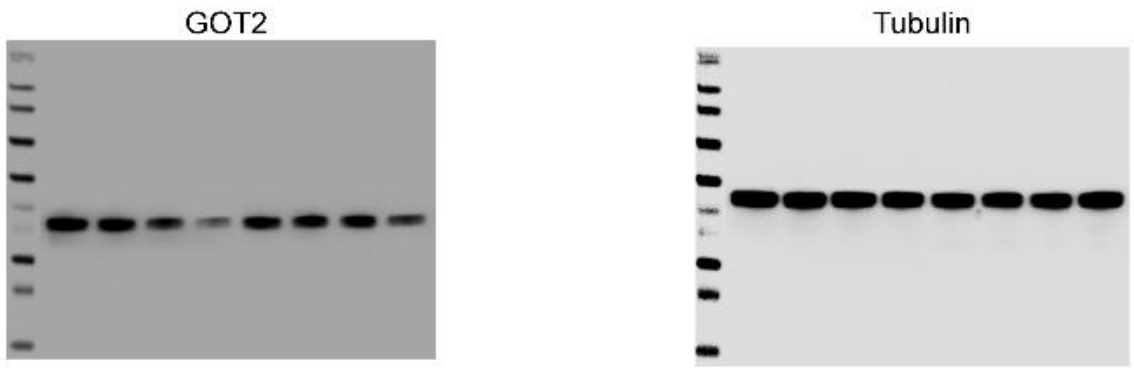

Figure 5J

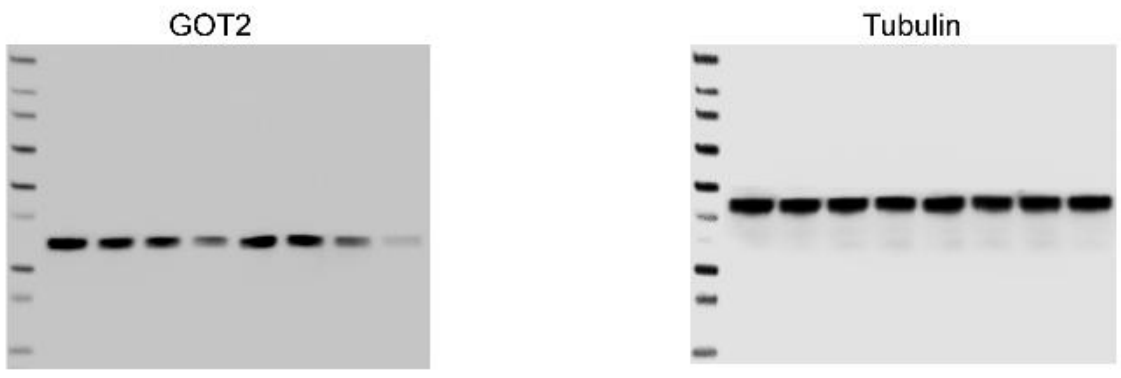

Figure 5K

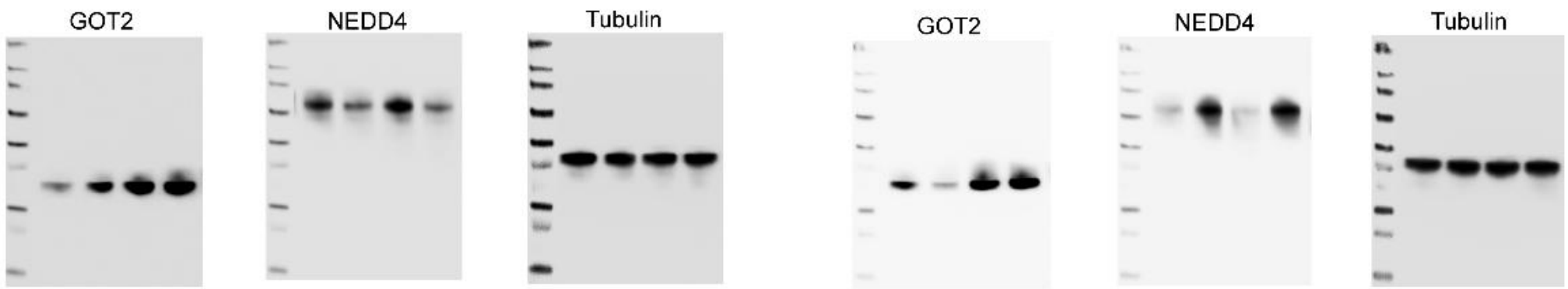

Figure 5L

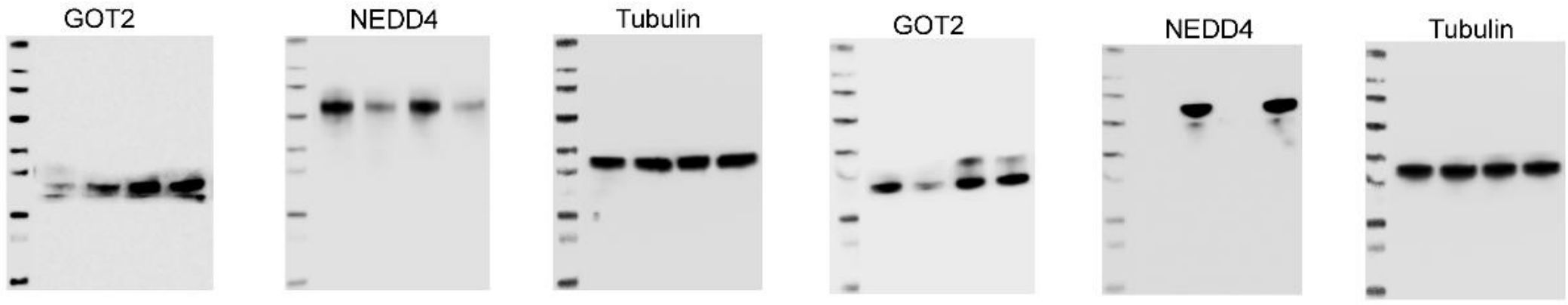

Figure 5M

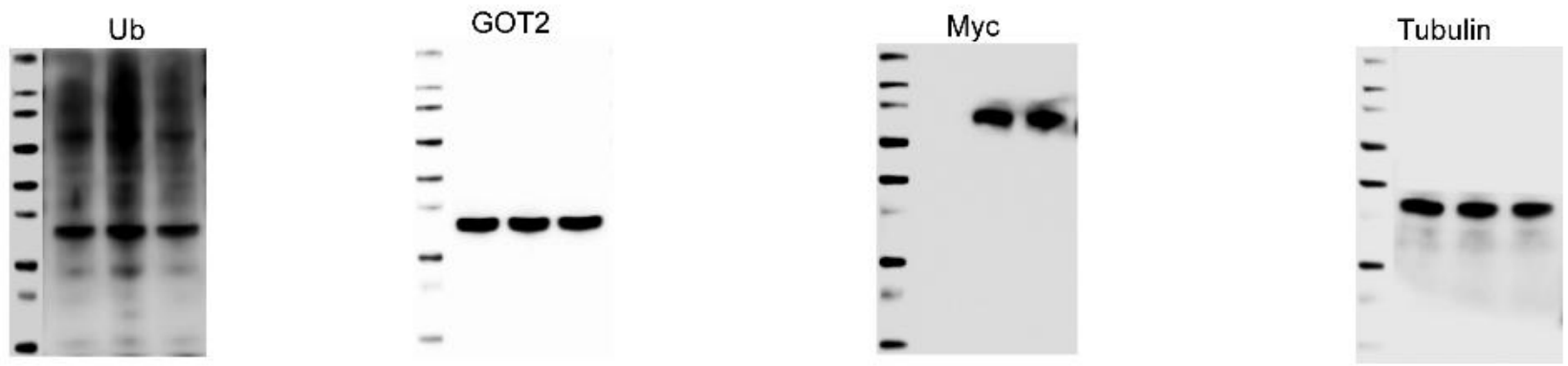

Figure 5N

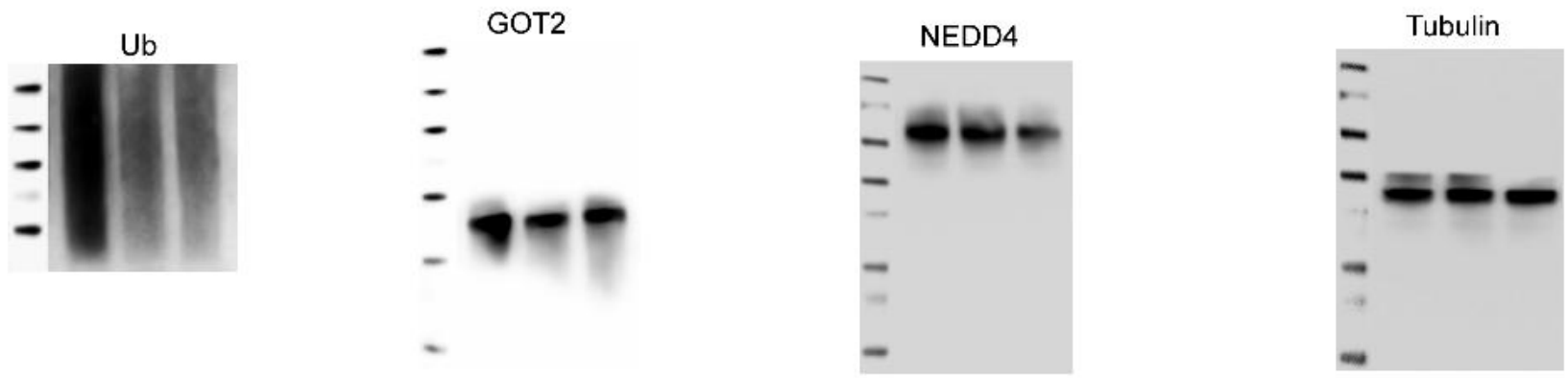

Figure 5O

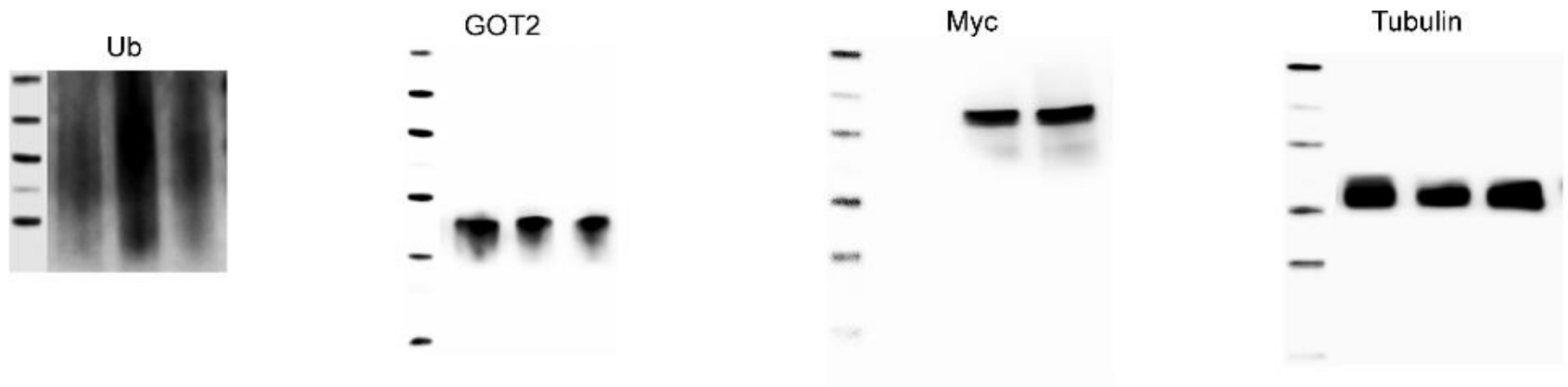

Figure 6A

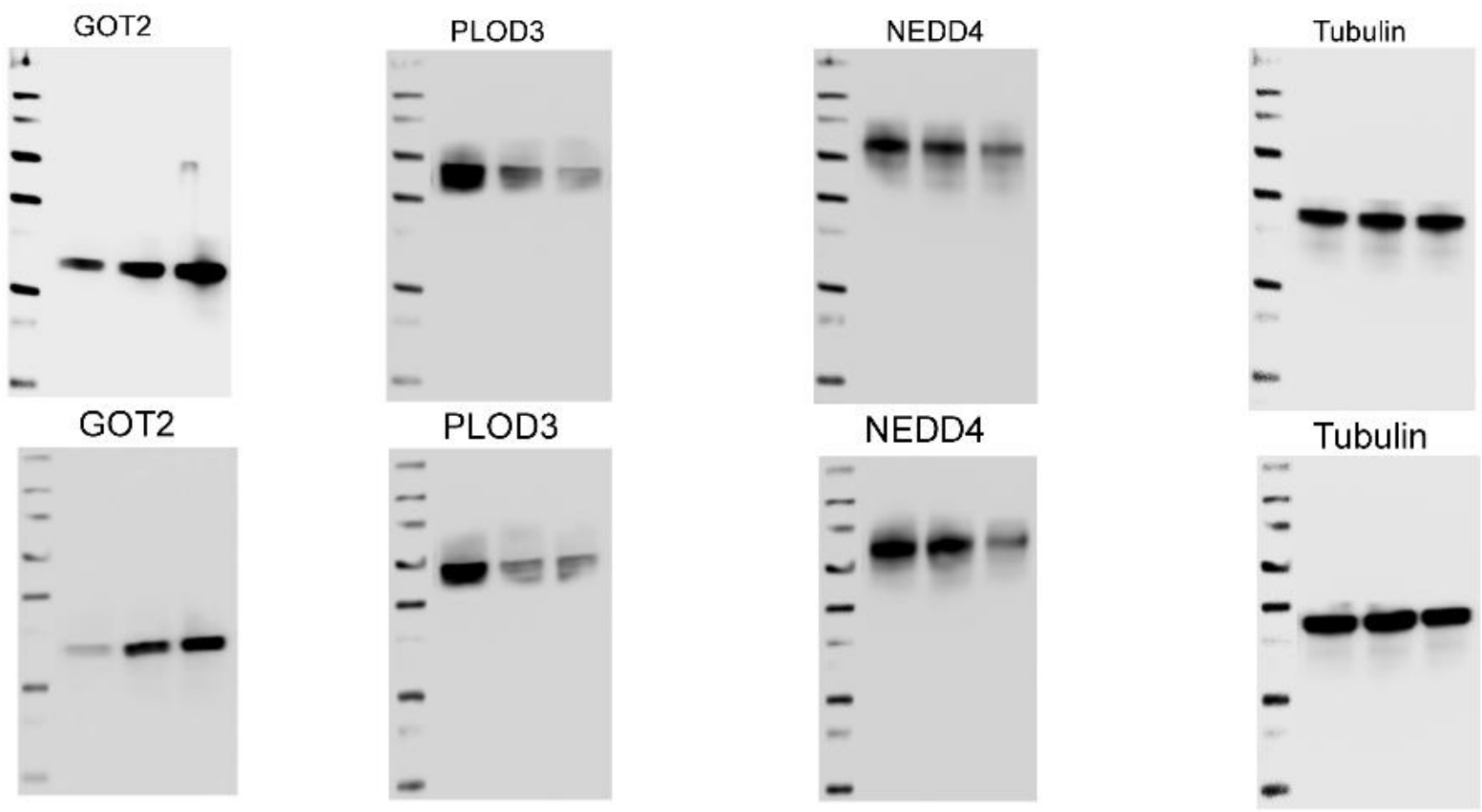

Figure 6B

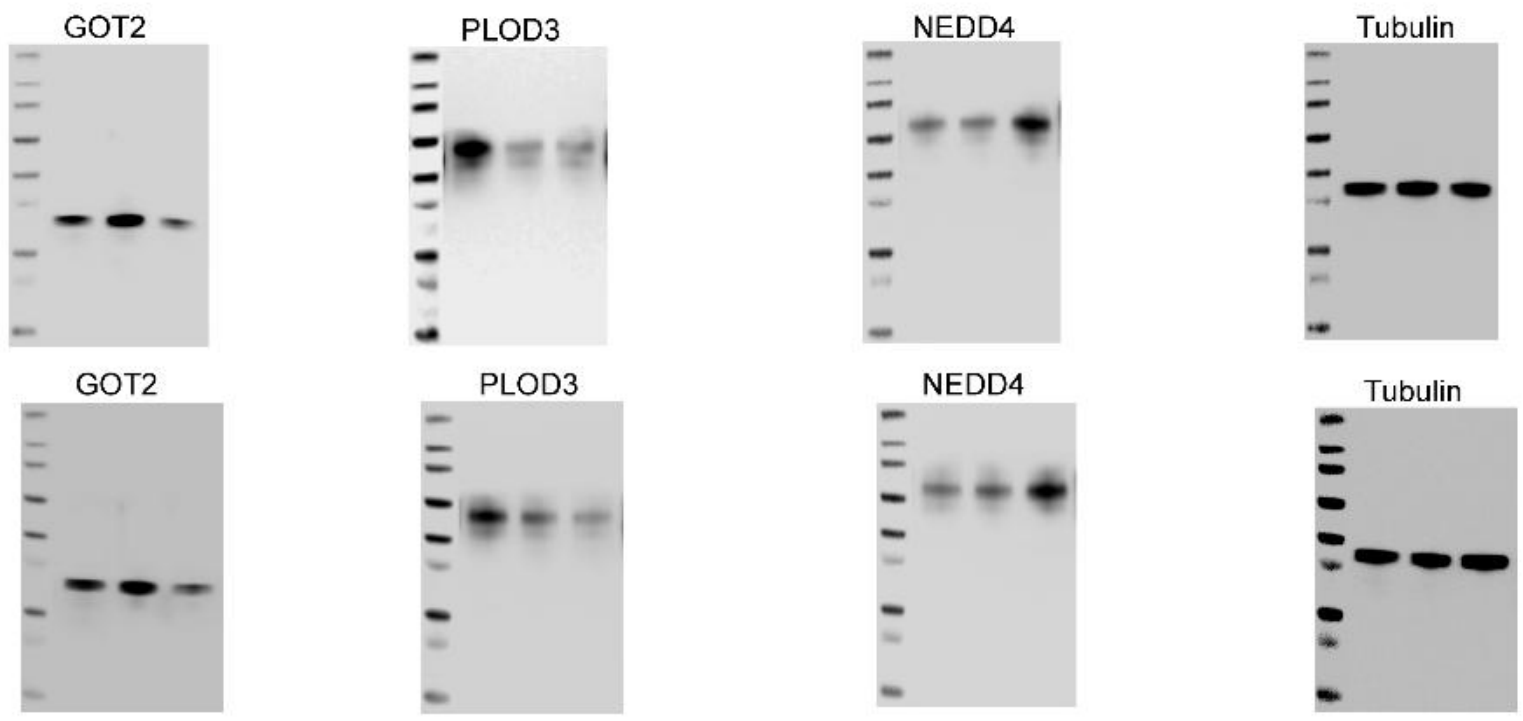

Figure 6C

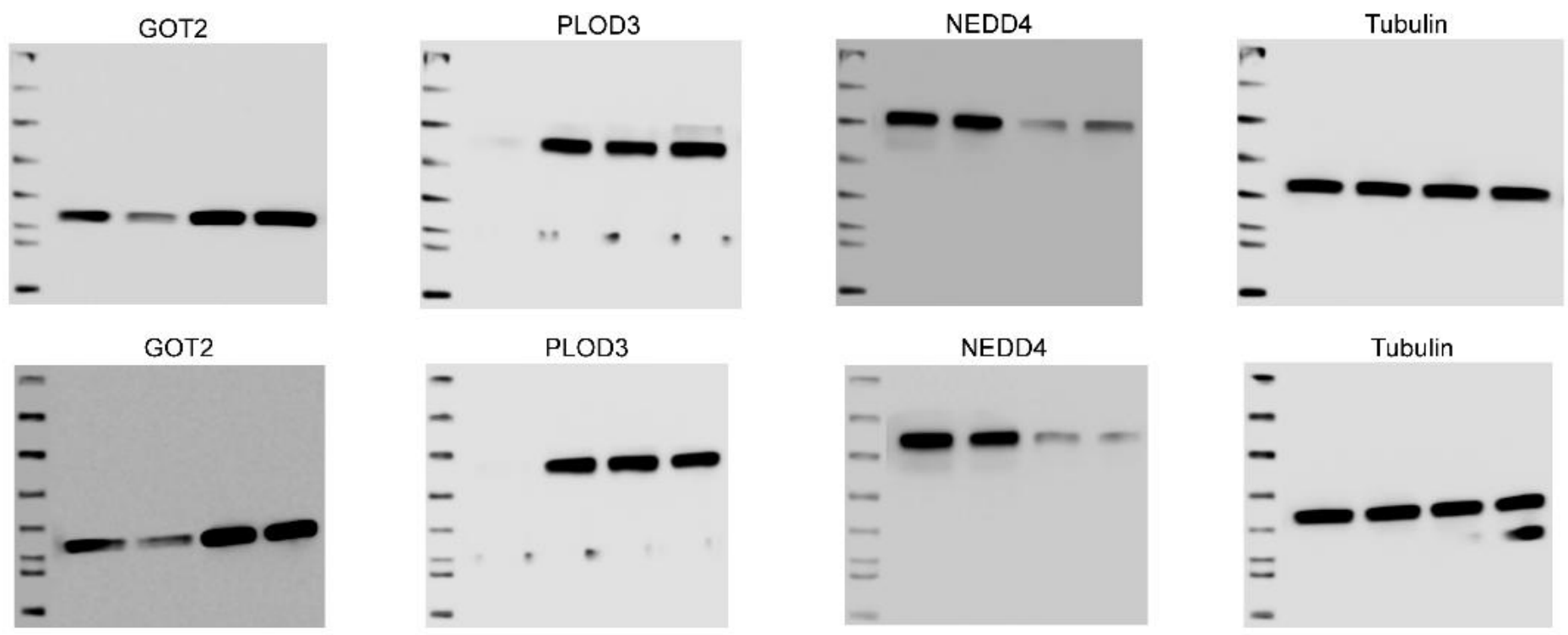

Figure 6D

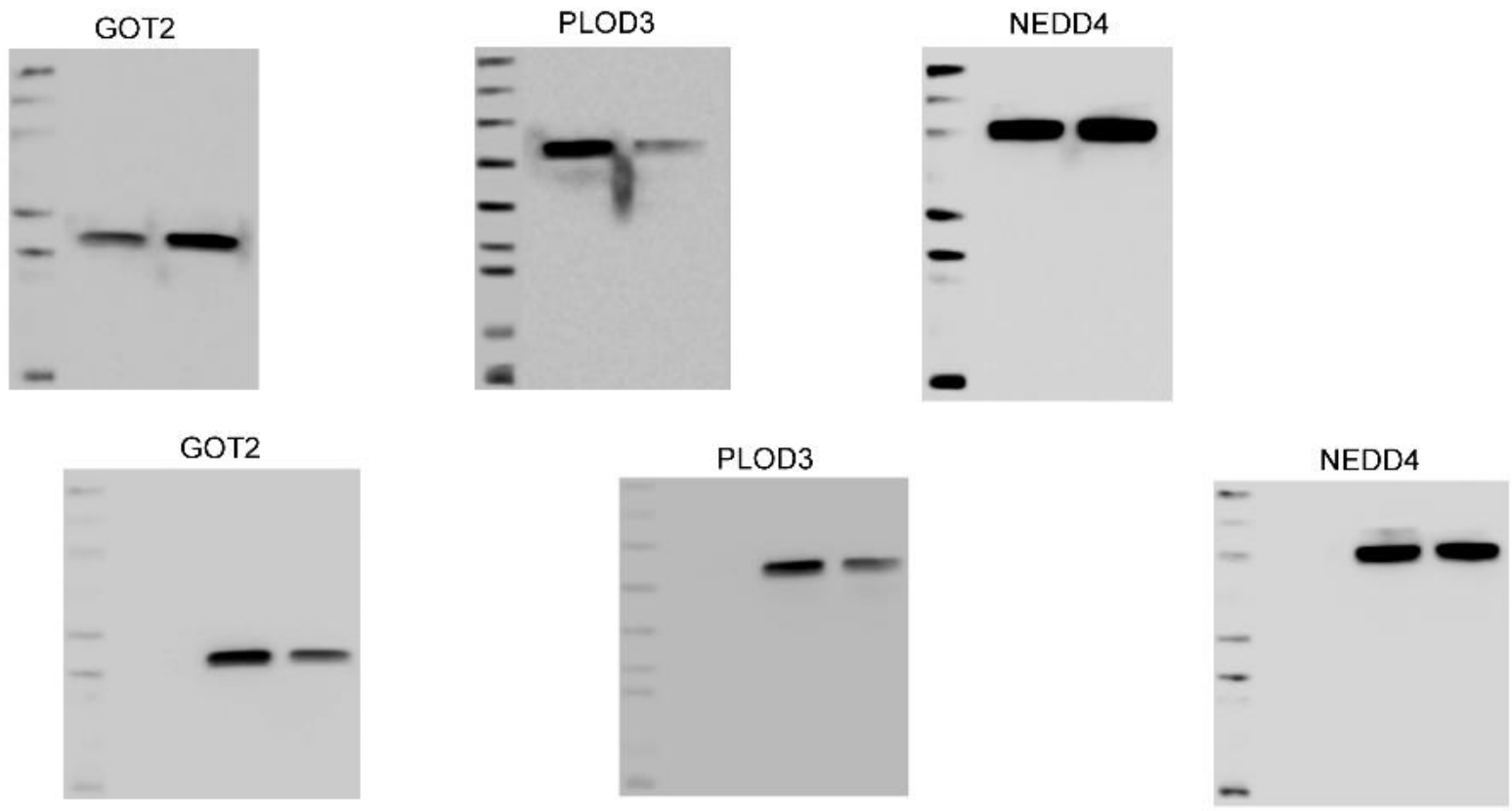

Figure 6E

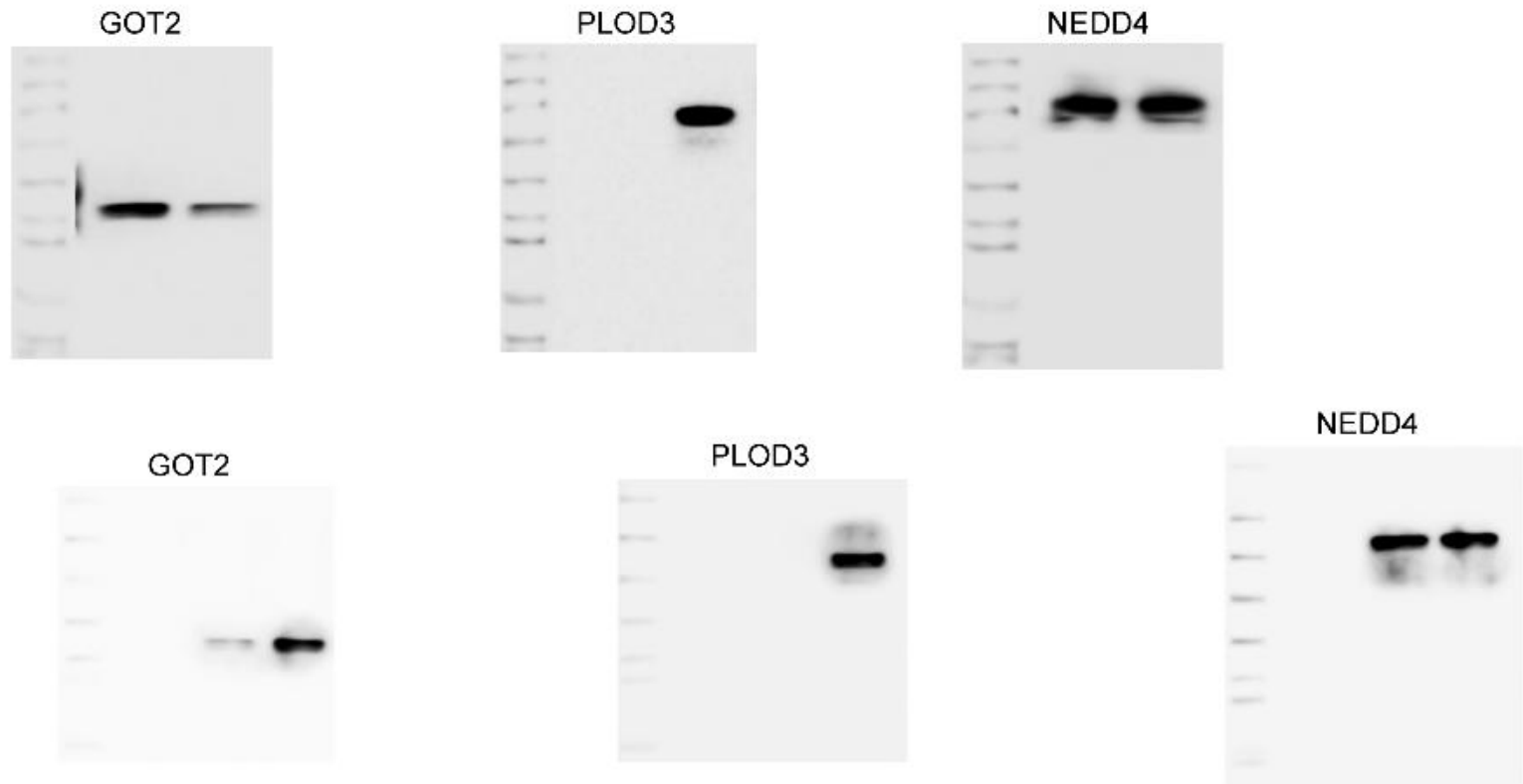

Figure 6F

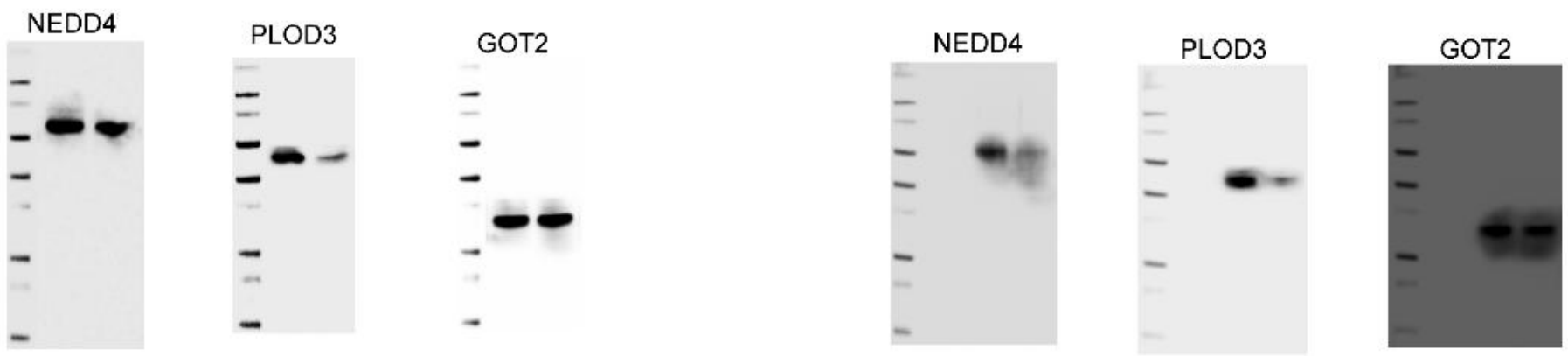

Figure 6G

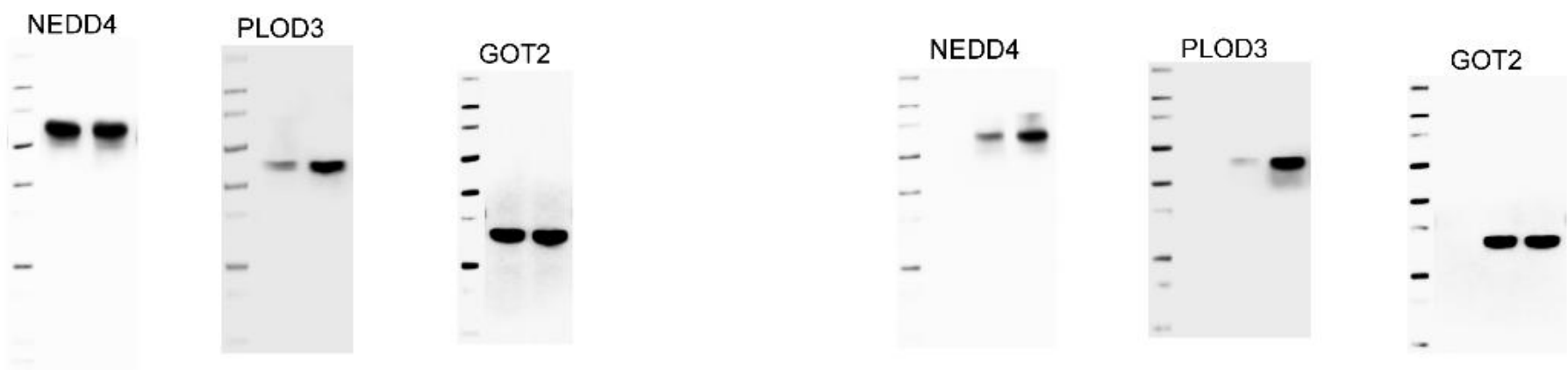

Figure 6H

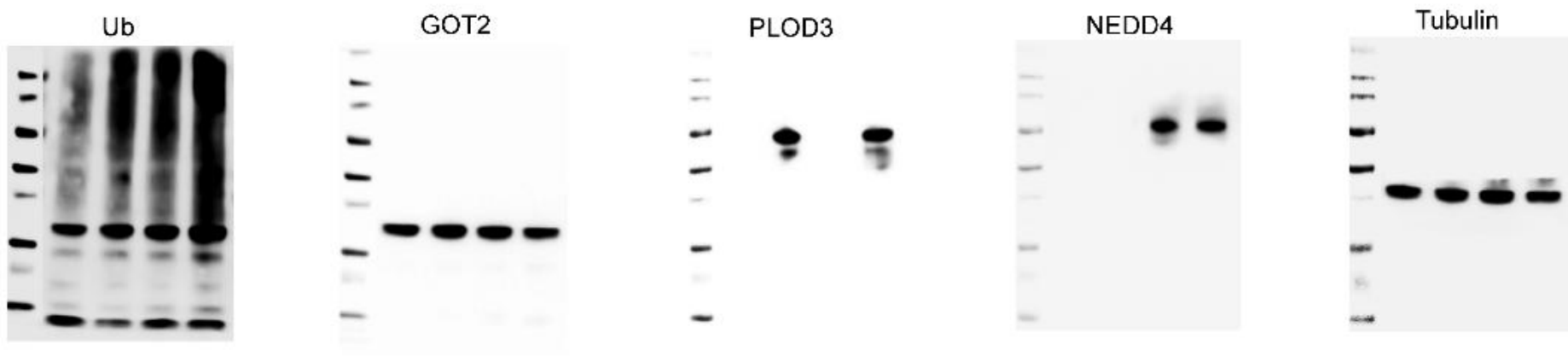

Figure 6I

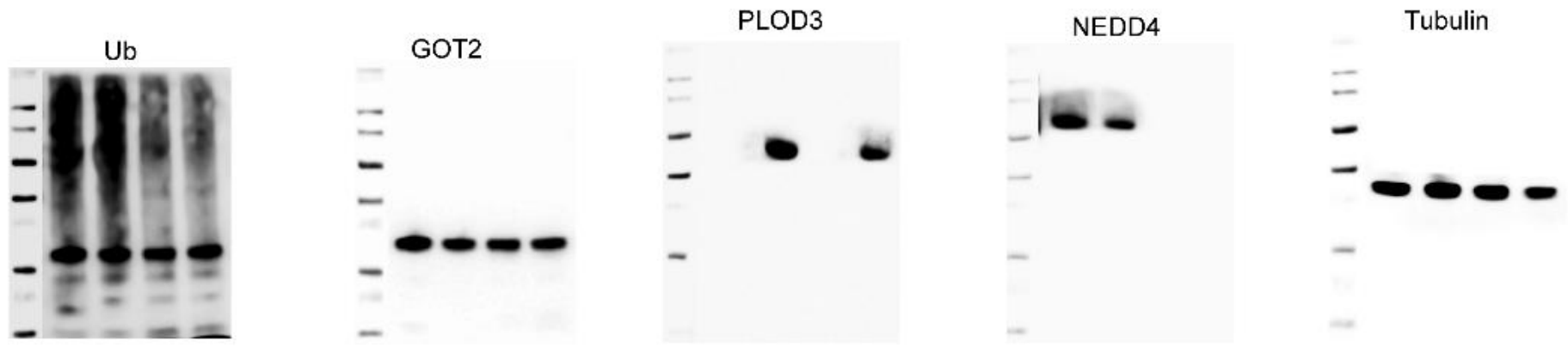

Figure 6J

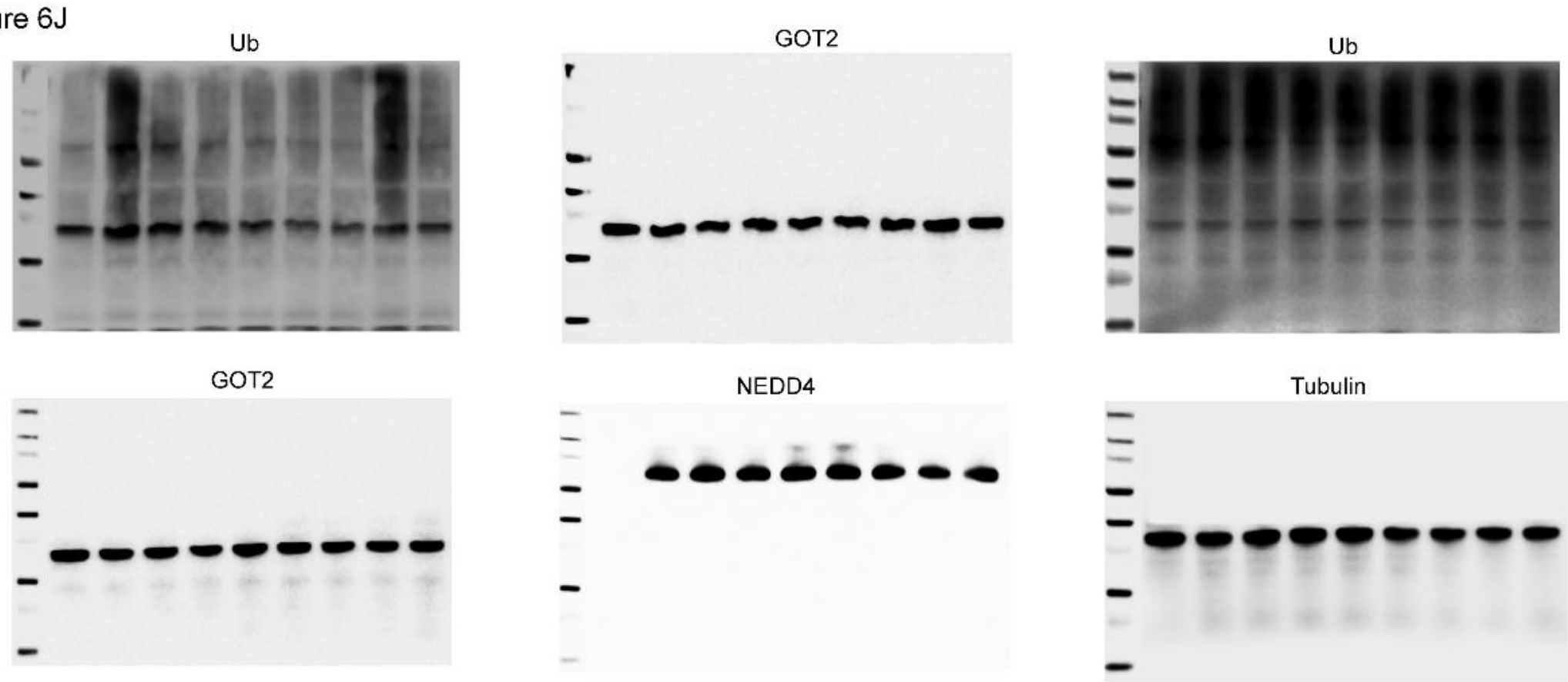

Figure 6K

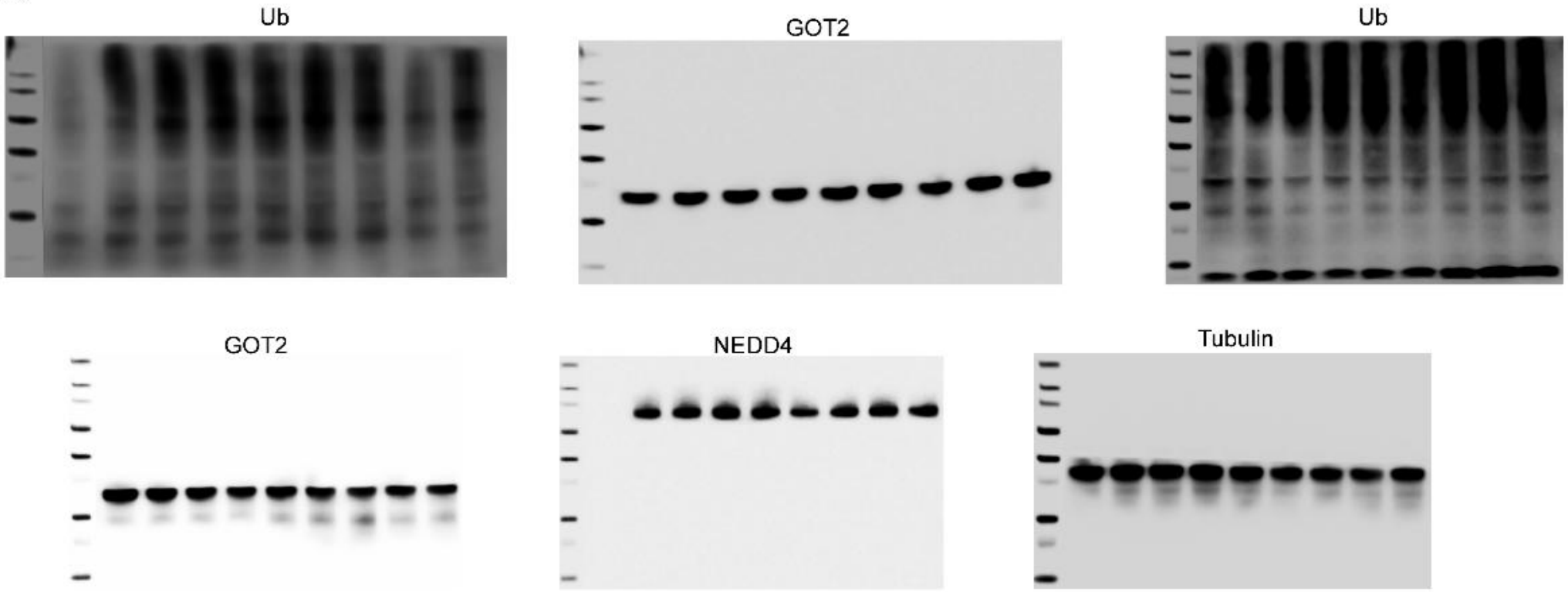

Figure 6L

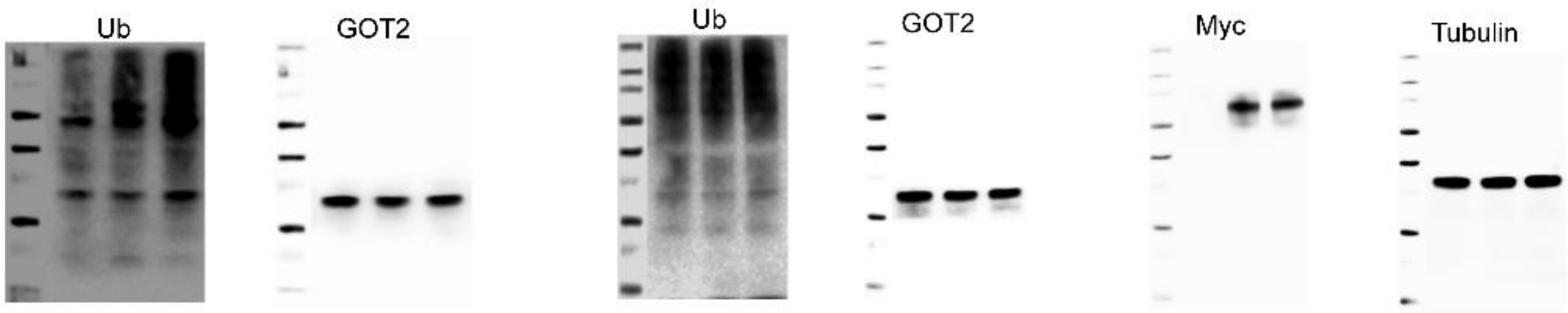

Figure 6M

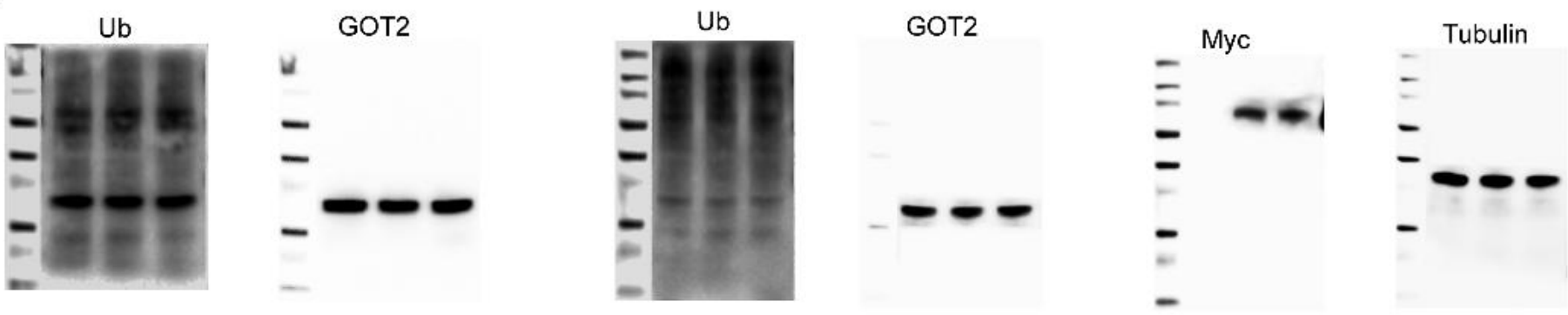

Figure 8A

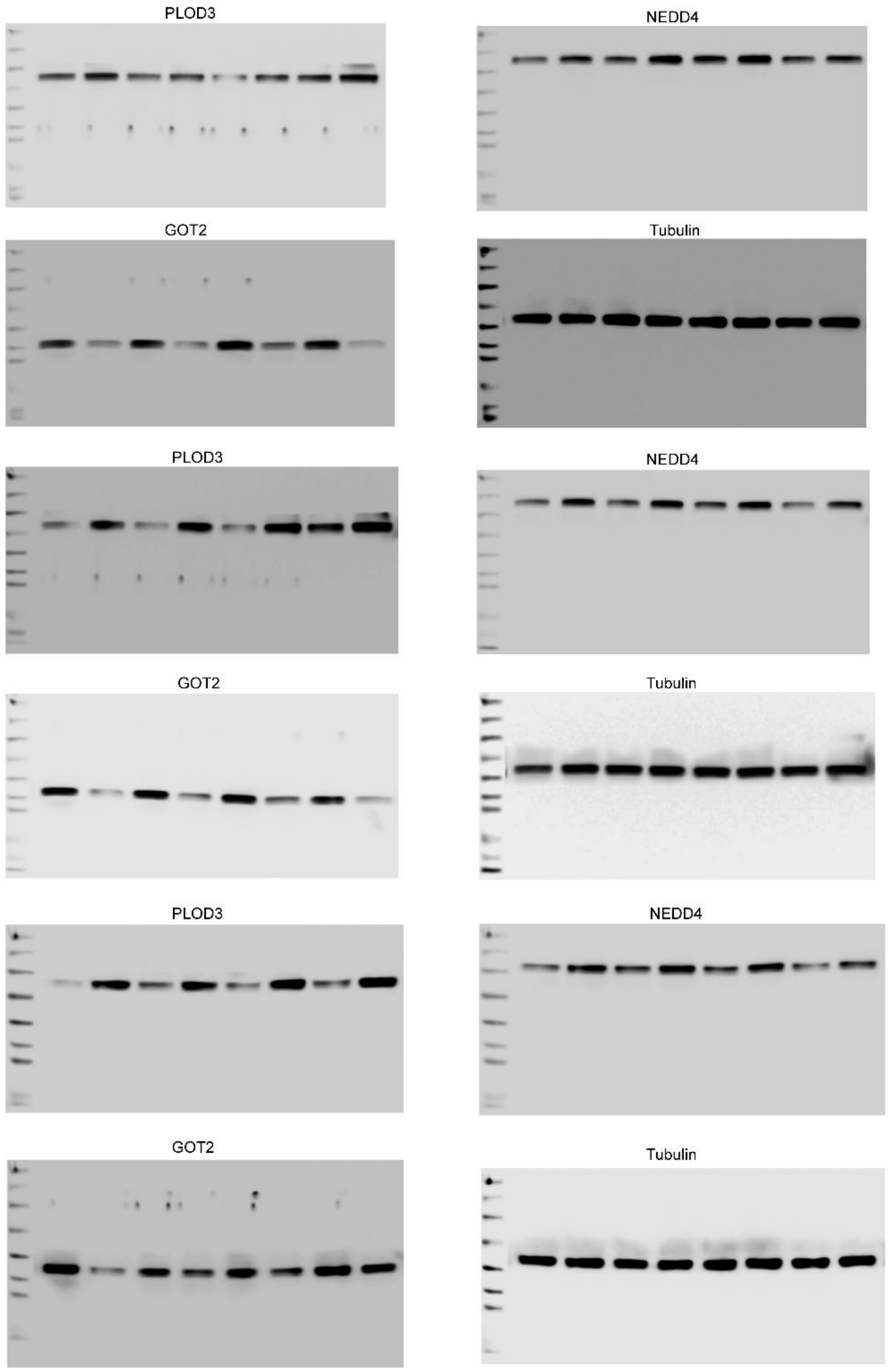
PLOD3
NEDD4
GOT2
Tubulin
PLOD3
NEDD4
GOT2
Tubulin
PLOD3
NEDD4
GOT2
Tubulin

Figure S4B

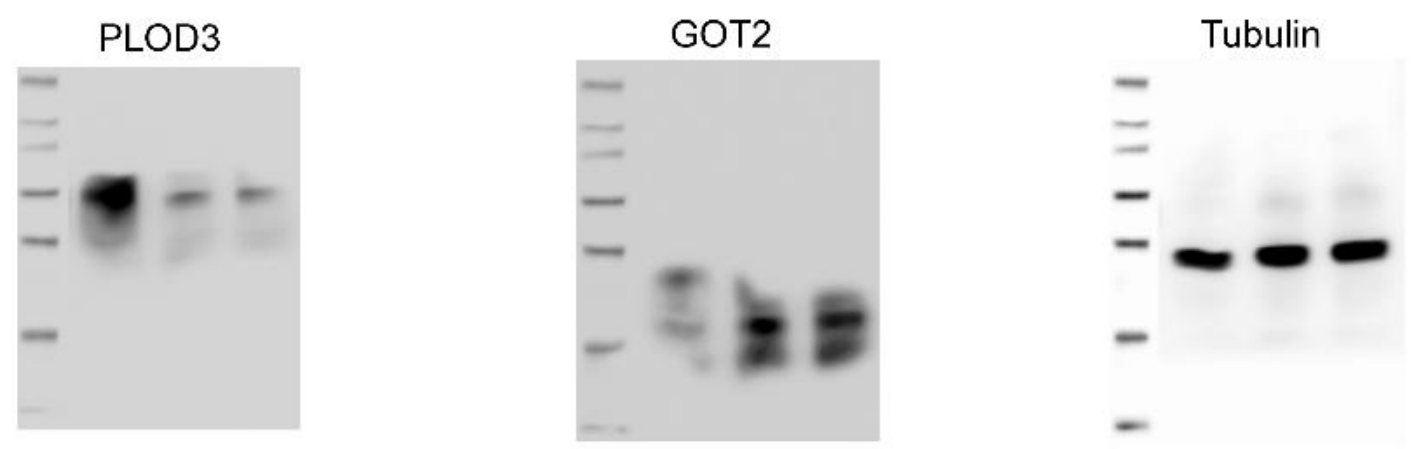

Figure S4C

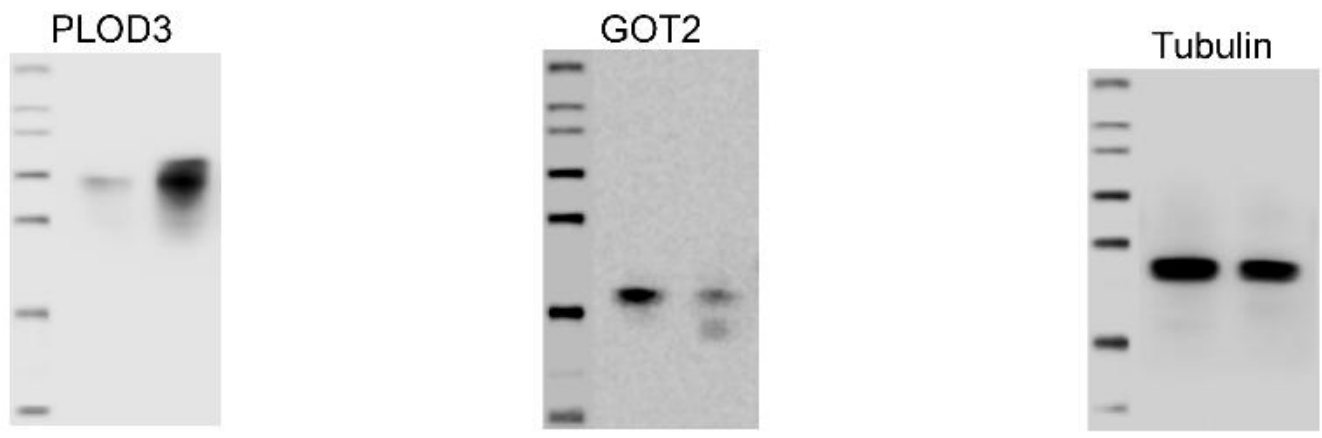

Figure S5A

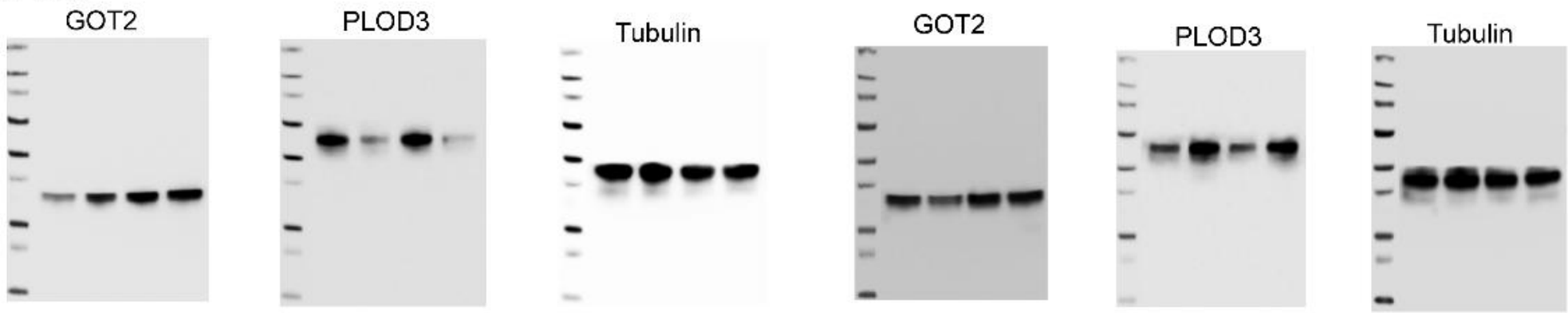

Figure S5B

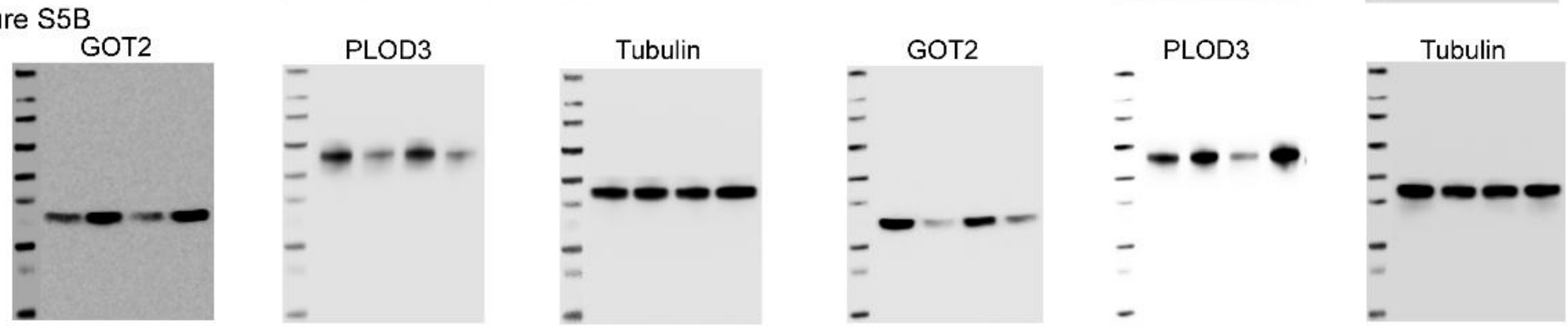

Figure S5C

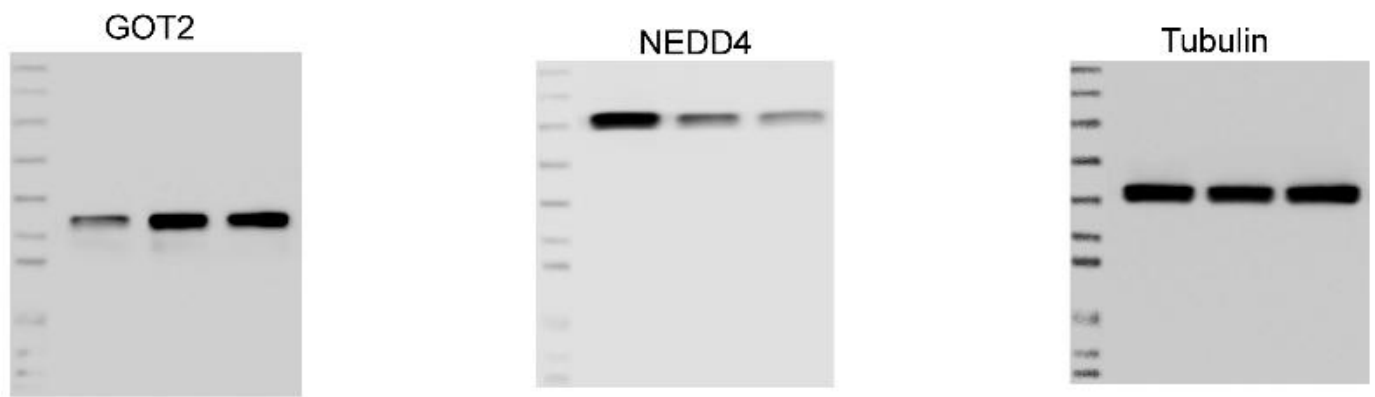

Figure S6A

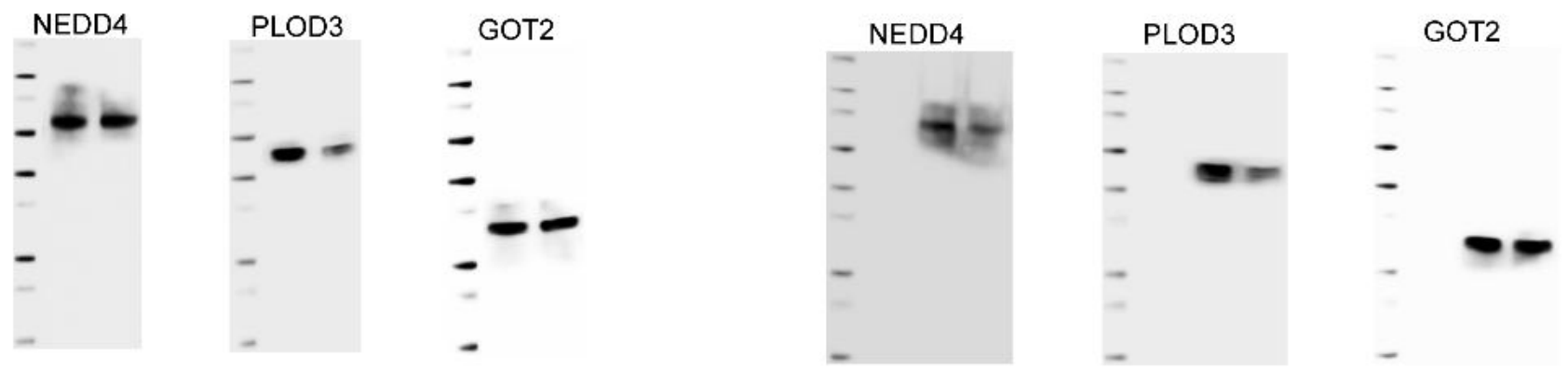

Figure S6B

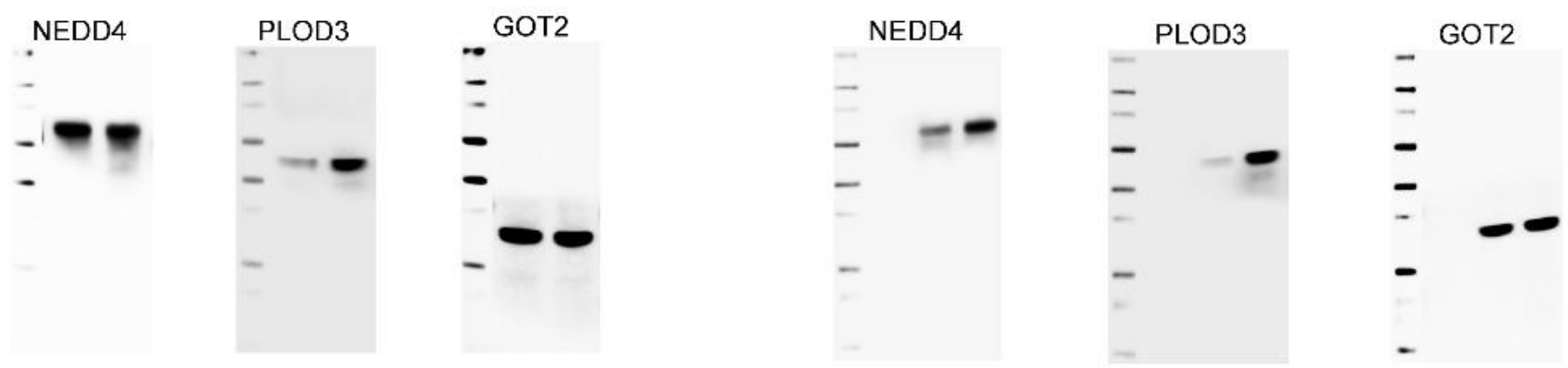

Figure S6C

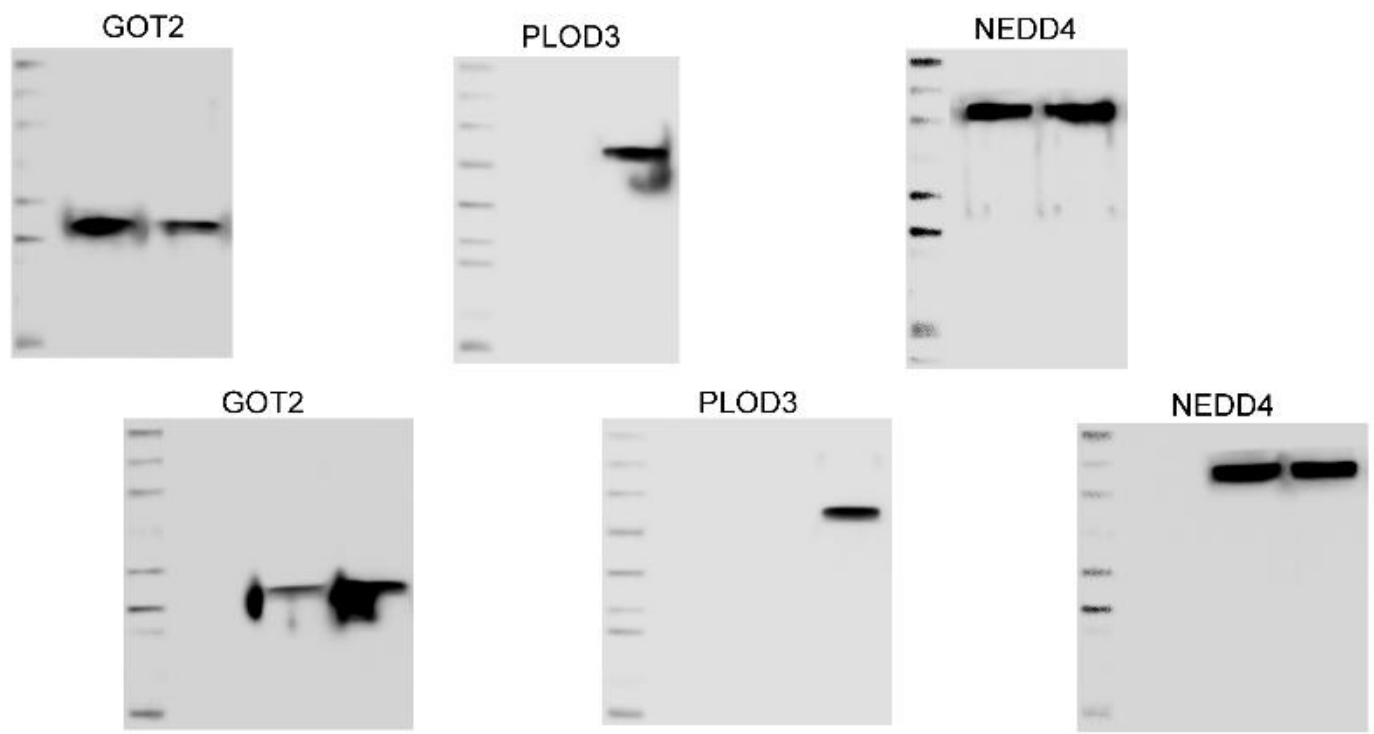

Supplement: Supplementary file 3 [file 41419_2026_8961_MOESM3_ESM.pdf]
